# Sterically controlled 5-*exo*-dig cyclization enables synthesis of non-benzenoid polycyclic aromatic hydrocarbons with intriguing (anti)aromaticity and diradical properties

**DOI:** 10.1039/d6sc00121a

**Published:** 2026-05-11

**Authors:** Liangliang Chen, Zhichun Shangguan, Tianyu Shi, Liyuan Qin, Yiyun Zeng, Qingqiu Zhu, Jin Chen, Junhong Liang, Wentao Miao, Yurong He, Xiaosong Qiu, Xunchang Wang, Deqing Zhang, Renqiang Yang

**Affiliations:** a Key Laboratory of Flexible Optoelectronic Materials and Technology (Ministry of Education), School of Optoelectronic Materials & Technology, Jianghan University Wuhan 430056 China chenliangliang@jhun.edu.cn yangrq@jhun.edu.cn; b College of Chemistry and Materials Engineering, Wenzhou University, Key Lab of Biohealth Materials and Chemistry of Wenzhou Wenzhou 325027 China; c Beijing National Laboratory for Molecular Sciences, Institute of Chemistry, Chinese Academy of Sciences Beijing 100190 China

## Abstract

Non-benzenoid polycyclic aromatic hydrocarbons (PAHs) containing antiaromatic indacene or pentalene and aromatic azulene subunits emerged as compelling materials, distinguished by their unique electronic configurations, exceptional optoelectronic characteristics, and potential applications in organic electronics. However, their controllable synthesis remains challenging due to inherent instability and stringent electronic requirements. Herein, we present a modular synthetic strategy that enables the construction of stable non-benzenoid PAHs (1, 2, and 3) featuring indacene, pentalene, and azulene motifs through a carefully designed sequence of 5-*exo*-dig cyclization (with controllable *E*/*Z*-selectivity), nucleophilic addition, Friedel–Crafts cyclization and oxidative dehydrogenation. Comprehensive structural and electronic analyses revealed that 1 and 2 exhibit global antiaromaticity and 2 displays a more pronounced open-shell diradical character than 1, while 3 maintains a global aromaticity and a closed-shell structure. Notably, compound 2 demonstrated promising p-type semiconductor behavior with a hole mobility of up to 0.083 cm^2^ V^−1^ s^−1^. Additionally, all three compounds demonstrated remarkable stability under ambient conditions, underscoring their potential for practical applications in organic electronics.

## Abstract 2

A modular synthetic strategy based on sterically controlled 5-*exo*-dig cyclization enables the construction of stable non-benzenoid PAHs featuring indacene, pentalene, and azulene motifs, exhibiting tunable (anti)aromaticity and diradical character.

## Graphical abstract

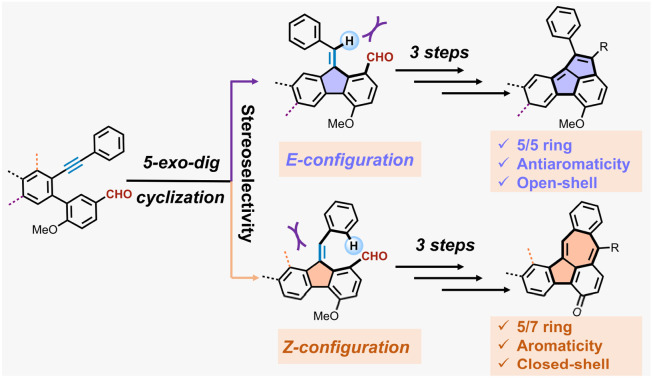

## Introduction

Over the past decade, non-benzenoid polycyclic aromatic hydrocarbons (PAHs) featuring pentagonal and heptagonal rings have emerged as a vibrant research frontier in materials chemistry.^1–9^ These structurally unique systems exhibit fundamentally different electronic properties compared to their benzenoid counterparts, owing to their characteristic structural strains, electronic character, and non-alternant π-conjugation networks.^10–19^ Particularly noteworthy are three archetypal building blocks, antiaromatic indacene (5/6/5-fused rings with 12 peripheral π-electrons), antiaromatic pentalene (5/5-fused rings with 8 π-electrons), and aromatic azulene (5/7-fused rings with 10 π-electrons). Integrating these motifs into extended π-conjugated systems enables precise modulation of electronic structures and molecular geometries.^20–22^ Antiaromatic indacene and pentalene units impart remarkable open-shell characteristics, facilitating unusual spin interactions and enhancing electron delocalization,^23–25^ while azulene moieties introduce strong dipole moments and intramolecular charge transfer capabilities, resulting in tunable absorption and emission properties.^26–32^ These distinctive features give rise to novel optoelectronic behaviors and spin-related phenomena, positioning non-benzenoid PAHs as promising candidates for advanced applications in organic electronics, singlet fission systems, and molecular magnetism.^33–37^ The ability to fine-tune these properties through rational molecular design has sparked growing interest in developing synthetic methodologies for these structurally challenging yet functionally versatile materials.

**Scheme 1:**
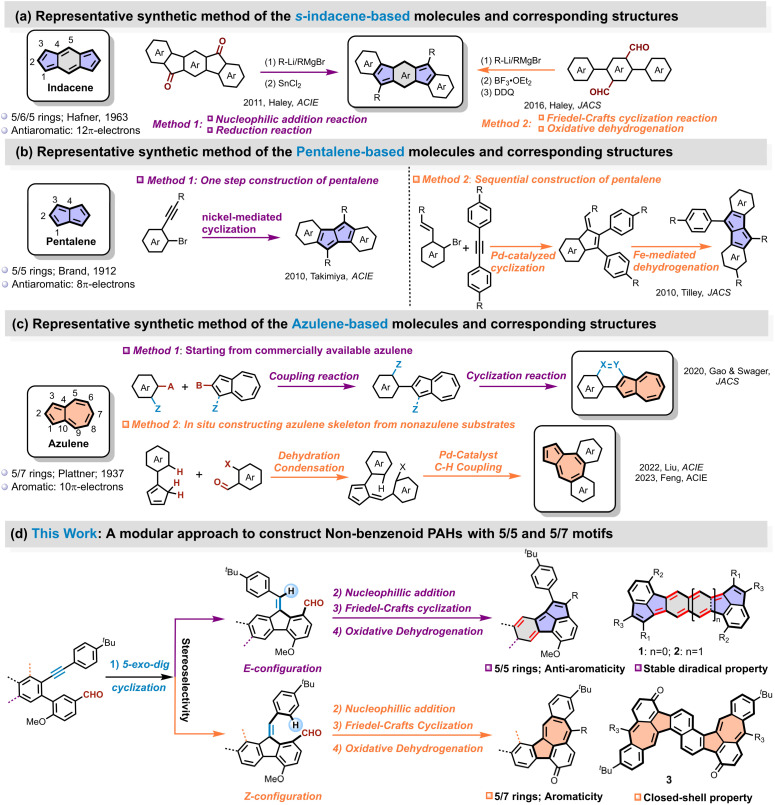
The representative synthetic strategies for (a) indacene, (b) pentalene and (c) azulene motifs-based non-benzenoid PAHs. (d) The modular synthetic approach to construct 5/5 and 5/7 motifs of this work.

**Fig. 1:**
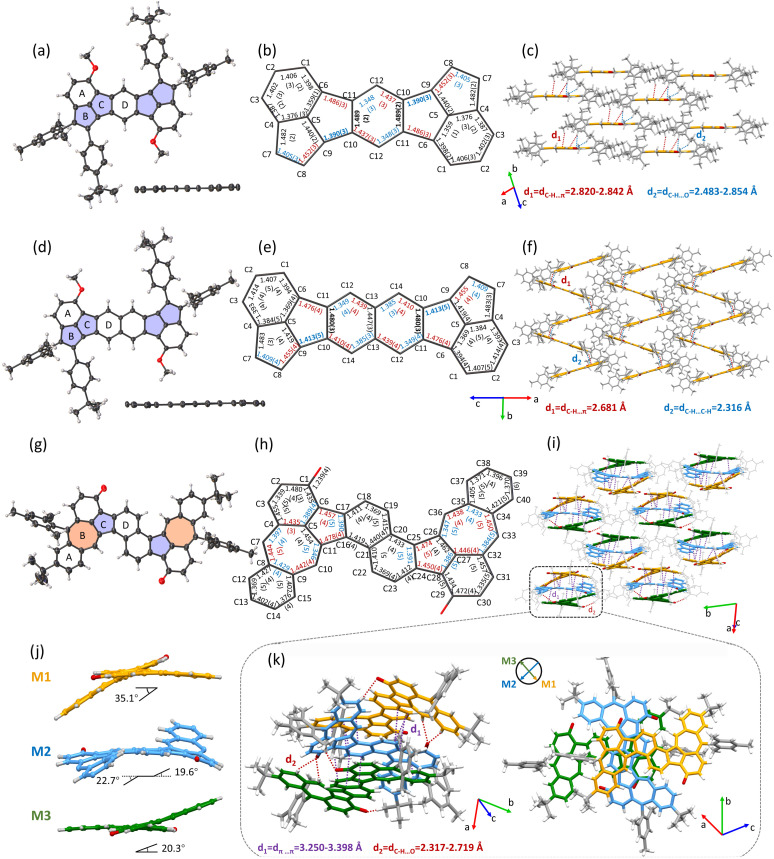
X-ray single-crystal crystallographic analysis. Molecular structure of top/side view of (a) 1, (d) 2 and (g) 3. The bond-length analysis of (b) 1, (e) 2 and (h) 3. The intermolecular packing mode and interaction of (c) 1, (f) 2 and (i) 3. (j) The three molecular twist conformation and the dihedral angle of 3 in a cell unit. (k) The intermolecular packing mode and interaction of the trimer of 3 in a cell unit.

The formidable challenge is mainly attributed to the inherent instability and complex electronic requirements for synthesizing these subunit-based molecular materials. A notable example is the first synthesis of *s*-indacene by Hafner and coworkers in 1963, which yielded an unsubstituted derivative that exhibits poor stability, being highly sensitive to oxygen and acids, and was inadequately characterized.^38,39^ Recent advances in synthetic methodologies have facilitated more precise construction of these architectures, thereby unlocking new opportunities for their practical implementation. Currently, *s*-indacene-based molecules can be prepared through two primary routes: (1) sequential nucleophilic addition and reductive elimination reactions starting from dicarbonyl (ketone) compounds,^23,40^ or (2) a combination of nucleophilic addition of aldehyde, Friedel–Crafts cyclization, and oxidative dehydrogenation reactions (Scheme 1a).^24,41^ Building on these approaches, Haley, Tobe, Chi, Müllen *etc*. successfully prepared a series of *s*-indacene derivatives exhibiting open-shell characteristics,^42–46^ where the diradical character was mainly localized at the C1 position of the *s*-indacene core through steric protection with bulky substituents (Fig. 1a).

Similarly, another notable antiaromatic subunit, pentalene, which consists of two fused five-membered rings with 8 π-electrons, is also inherently unstable except for sterically protected derivatives or annulated with other aromatic rings.^47–49^ Owing to the fused five-membered rings (5/5), pentalene exhibits a distinct electronic structure compared with the dicyclopenta-fused arenes, which were initially discovered by Garcia-Garibay and later pursued by Plunkett.^50–53^ The main synthetic routes to pentalene derivatives include: (1) a one-step Pd-catalyzed or Ni-catalyzed cyclodimerization of *ortho*-bromophenylacetylene derivatives with low yield (Scheme 1b),^54,55^ or (2) a two-step sequence involving Pd-catalyzed cyclization followed by Fe-mediated dehydrogenation.^56^ In addition, Hashmi and coworkers reported a gold-catalyzed regiospecific annulation of unsymmetrically substituted 1,2-di(arylethynyl)benzenederivatives for a geometry-controlled synthesis of linear bispentalenes.^57^ Yasuda and coworkers synthesized a dibenzo[a,f]pentalene by sequential nucleophilic addition of aldehyde, Friedel–Crafts cyclization and oxidative dehydrogenation processes, which exhibited antiaromatic and singlet biradical characters.^47,58^ Despite these rapid developments in synthetic strategies, the central or side benzene or naphthalene moiety in these molecules (Fig. 1b) remains strongly aromatic except for the pentalene units, preserving overall stability. Nevertheless, combining two antiaromatic subunits of *s*-indacene and pentalene into one molecule, simultaneously, has not been explored due to synthetic challenges. Additionally, it is also unclear how these two antiaromatic units affect each other and their impact on the overall performance of the molecule.

In comparison, azulene-containing conjugated systems benefit from relatively higher stability among these three motifs, leading to the development of diverse synthetic approaches. Traditional methods for constructing azulene units, such as those employing pyridinium salts or troponoids, face limitations when applied to larger azulene-embedded PAHs due to low reactivity and challenges in accessing appropriate benzo-fused precursors.^15^ Consequently, alternative strategies have emerged for *in situ* construction of azulene subunits within extended π-systems, including both on-surface synthesis,^59–64^ and in-solution chemistry. These encompass Scholl-type cyclization,^65–67^ intramolecular Friedel–Crafts reactions followed by aromatization,^31,68^ Pd-catalyzed alkyne annulation,^69–71^*etc.*^72–74^ However, these transformations typically require multi-step synthesis of specialized precursors and are often complicated by rearrangement or insertion side reactions, leading to unpredictable product distributions.^28^ For more controllable synthesis of azulene-based PAHs, a common strategy involves starting with commercially available azulene and performing sequential coupling and cyclization reactions to construct azulene-terminated PAHs (Scheme 1c).^75–80^ However, the azulene unit in these azulene-terminated PAHs exhibits little effect on the optoelectronic properties of the whole molecules owing to the relatively independent local electronic structure characteristic. In contrast, azulene-embedded PAHs integrate azulene into the fused ring backbone, which directly tunes the π-conjugation pathway. For example, Feng and Liu recently reported a modular approach to azulene-embedded PAHs using a cascade reaction combining Suzuki coupling and Knoevenagel condensation (Scheme 1c).^81–83^ Nevertheless, these synthetic methods generally require complex precursor preparation and are often limited to specific substrate types. Therefore, the development of simpler, more controllable, and broadly applicable synthetic strategies for preparing non-benzenoid PAHs with tunable structures and properties is highly desirable, especially for these azulene-embedded linear PAH.

Herein, we developed an innovative synthetic strategy combining 5-*exo*-dig cyclization of alkyne, nucleophilic addition of aldehyde, Friedel–Crafts cyclization at the exocyclic double-bond or substituted phenyl position and oxidative dehydrogenation processes to construct two highly stable antiaromatic PAH combining an indacene skeleton and two pentalene (5/5) motifs (1 and 2) and an S-shaped azulene (5/7)-embedded linear PAH (3). The product selectivity was governed by the *E*/*Z* configuration of key intermediates (7, 13, and 19) obtained through 5-*exo*-dig cyclization. Detailed structural characterization reveals that 1 and 2 exhibited global antiaromaticity, while 3 exhibits global aromaticity along its molecular periphery, supported by bond length analysis of crystal and theory calculation. Notably, 2 adopts a planar configuration and demonstrates significant open-shell character (diradical index *y*_0_ = 0.38) with a small singlet–triplet energy gap (Δ*E*_st_ = −4.54 kcal mol^−1^). In contrast, 1 maintains a closed-shell configuration, while 3 exhibits a twisted geometry resulting from the cove edge between naphthalene and azulene moieties, along with closed-shell characteristics. Remarkably, 2 demonstrates promising p-type transporting behavior, achieving a hole mobility up to 0.083 cm^2^ V^−1^ s^−1^ under an air atmosphere. This synthetic strategy provides a versatile and controllable approach for exploring diverse 5/5 or 5/7 rings-based non-benzenoid PAHs, from chemical structure, optoelectronic properties, to applications in organic electronics.

## Results and discussion

### Synthesis and characterization

**Scheme 2:**
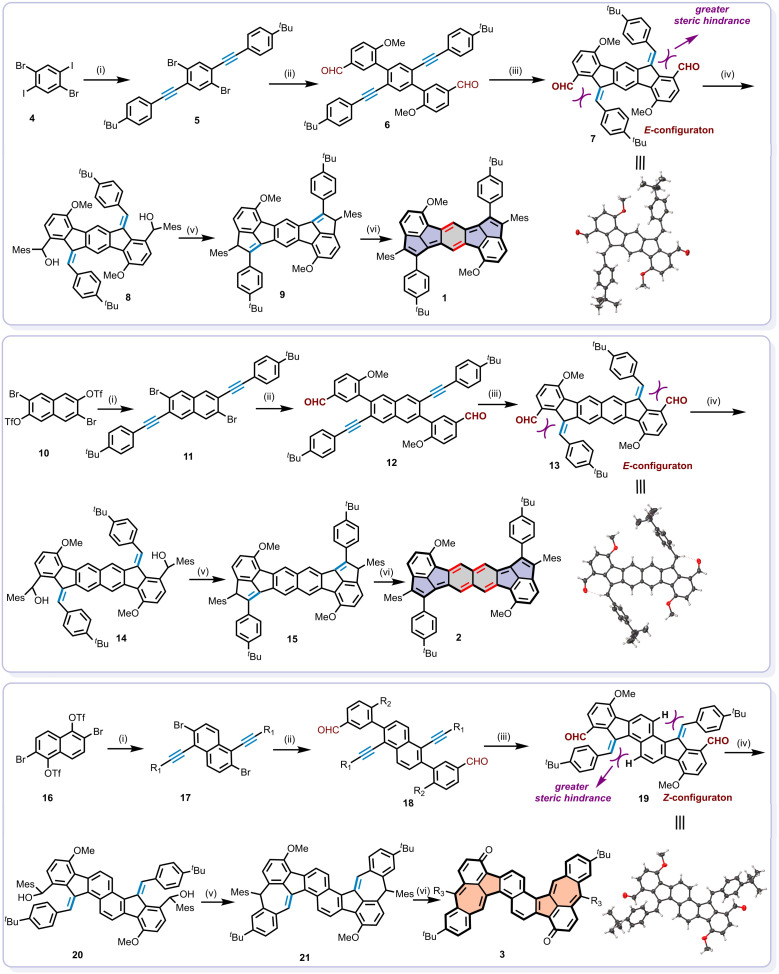
The synthetic routes of (a) 1, (b) 2 and (c) 3. (i) 4-Tert-butylphenylacetylene, Pd(PPh_3_)_2_Cl_2_, CuI, diisopropylamine, DMF; (ii) 2-methoxy-5-formylphenylboronic acid, Pd(PPh_3_)_4_, K_2_CO_3_, tolunen/EtOH/H_2_O; (iii) Pd(OAc)_2_, Sphos, tri-*n*-butylphosphine, toluene; (iv) MesMgBr, THF; (v) BF_3_·OEt_2_, DCM; (vi) DDQ, toluene.

The synthetic routes towards compounds 1, 2 and 3 are illustrated in Scheme 2. Key precursors (compounds 6, 12, and 18) were prepared through sequential Sonogashira and Suzuki coupling reactions starting from 4, 10, and 16. These precursors subsequently underwent Pd(OAc)_2_-catalyzed 5-*exo*-dig cyclization in the presence of 2-dicyclohexylphosphino-2′,6′-dimethoxybiphenyl (Sphos) and tri-*n*-butylphosphine ligands to afford intermediates 7, 13, and 19.^84–86^ Interestingly, the exocyclic double bonds of compounds 7 and 13 adopted *E*-configuration, whereas 19 exhibited a *Z*-configuration due to steric hindrance imposed by its substituents. This stereochemical assignment was confirmed through comprehensive structural characterization. As shown in Fig. S1 and S2, NOESY spectra of 7 and 13 revealed strong correlation signals between *H*_c_ and *H*_d_, *H*_g_ and *H*_e_, consistent with *E*-configuration. Moreover, the crystals of 7 and 13, suitable for single-crystal structural analysis, were successfully obtained by slowly diffusing methanol into the chloroform solution, which unambiguously confirmed their structures as assigned (Scheme 2, Fig. S7 and S8). Notably, in the case of 13, the intramolecular hydrogen bond between the aldehyde oxygen and the exocyclic double bond hydrogen was observed in the crystal structure, potentially stabilizing the *E*-configuration. Alternatively, intermediate 19 was determined to possess a *Z*-configuration, as evidenced by NOESY correlations between *H*_c_ and *H*_e_, *H*_d_ and *H*_g_ (Fig. S3) and confirmed by single-crystal analysis (Scheme 2 and Fig. S9).

Then, dihydro-precursors of 9, 15 and 21 were obtained by the treatment of 7, 13, and 19 with mesitylmagnesium bromide (MesMgBr), followed by Friedel–Crafts cyclization with BF_3_·OEt_2_ (Scheme 2). Subsequently, the oxidative dehydrogenation of 9, 15 and 21 using 2,3-dichloro-5,6-dicyano-1,4-benzoquinone (DDQ) afforded the target products in moderate yield across three steps. Compound 1 was obtained as an army green solid with a yield of 17.3%, 2 as a purple solid with a yield of 63.3% and 3 as a brown solid with a yield of 19.7%. Specifically, although 1 and 2 share nearly identical molecular structures, one of the reasons for the lower yield of compound 1 may be attributed to the poor solubility owing to the densely 2D-brick-packed crystal lattice with stronger intermolecular interactions as discussed below. The chemical structures of 1, 2 and 3 were thoroughly characterized using nuclear magnetic resonance (NMR) spectroscopy, high-resolution mass spectra (HRMS), and X-ray single-crystal analysis. Detailed characterization data and experimental procedures are provided in the synthesis and characterization section of the SI.

### Single-crystal structure analysis

The single crystals of 1 and 2 were successfully grown by slowly diffusing methanol into the chloroform/CS_2_ (1/1, v/v) solution at −4 °C. As shown in Fig. 1, both 1 and 2 maintain nearly planar geometries, with significant steric hindrance from the mesityl and *tert*-butyl phenyl substituents preventing π–π stacking interactions between molecular backbones. 1 shows a one-dimensional parallel packing and the main interactions are multiple C–H⋯π with distances of 2.820–2.842 Å and C–H⋯O with distances of 2.483–2.854 Å (Fig. 1c), while 2 shows a herringbone stacking and is also dominated by C–H⋯π interactions (with shorter distances of 2.681 Å) and C–H⋯C–H interaction (2.316 Å) (Fig. 1f). Bond length analysis depicts that the C(sp^2^)–C(sp^2^) bond lengths for the periphery of 1 and 2 exhibit typical bond-distance alternation, ranging from 1.348 Å to 1.487 Å for 1 and 1.349 Å to 1.476 Å for 2, as expected for a closed-shell quinoid structure (Fig. 1b and e). Especially, the bond lengths of C10–C11 of the central benzene ring and naphthalene ring of 1 or 2 are 1.489 and 1.480 Å, indicating the aromaticity of the central benzene ring and naphthalene ring was disrupted, consistent with quinoid structures. The small bond length alternation and shorter distance of C10–C11 of 2 than 1 agree well with the small energy barrier for the valence tautomerization between the closed-shell quinoid and open-shell diradical structure. In addition, the distances of C9–C10 bonds in 1 and 2 are 1.390 and 1.413 Å, respectively, which fall between those of known closed-shell (1.371 Å) and open-shell (1.437 Å) Mes-substituted indenofluorene analogues.^87,88^ The intermediate value indicates 1 and 2 are resonance hybrids, with contributions from both open-shell and closed-shell structures in their ground states. Moreover, the slightly longer C9–C10 bond length in 2 compared to 1 further demonstrates that 1 exhibits more pronounced closed-shell character, while 2 leans toward open-shell features.

Similarly, single crystals of 3 suitable for X-ray diffraction analysis were obtained through slow diffusion of methanol into a chloroform solution at −4 °C. The molecular structure displays a distinctive twisted S-shaped conformation (Fig. 1j), resulting from steric repulsion between the naphthalene and azulene moieties at the cove region. Interestingly, the crystal packing exhibits an intriguing trimeric superstructure composed of repeating M1, M2, and M3 units. M1 and M3 adopt head-to-tail stacking with torsion angles of 35.1° and 20.3°, respectively, whereas M2 inserts nearly vertically between M1 and M3, with torsion angles of 22.7° (19.6°) (Fig. 1j). The main intermolecular interactions between adjacent molecules are π–π interactions with distances of 3.250–3.398 Å and C–H⋯O interactions with distances of 2.317–2.719 Å (Fig. 1i and k). Bond length analysis of 3 revealed that the C–C bond length of the periphery along the S-shaped skeleton exhibited an averaged characterization, despite the presence of localized bond length alternation in certain 5/7 ring segments with the longest bond length up to 1.478 Å (C11–C16) and the shortest bond length down to 1.346 Å (C10–C11), as shown in Fig. 1h, which are less averaged than that of the parent distinct azulene unit (1.387–1.427 Å).^89^ These results indicate that the azulene motif in this molecule is not independent, which exhibits a significant influence on molecular configuration, stacking, intermolecular interactions and electronic properties.

### Aromaticity and electronic structure

**Fig. 2:**
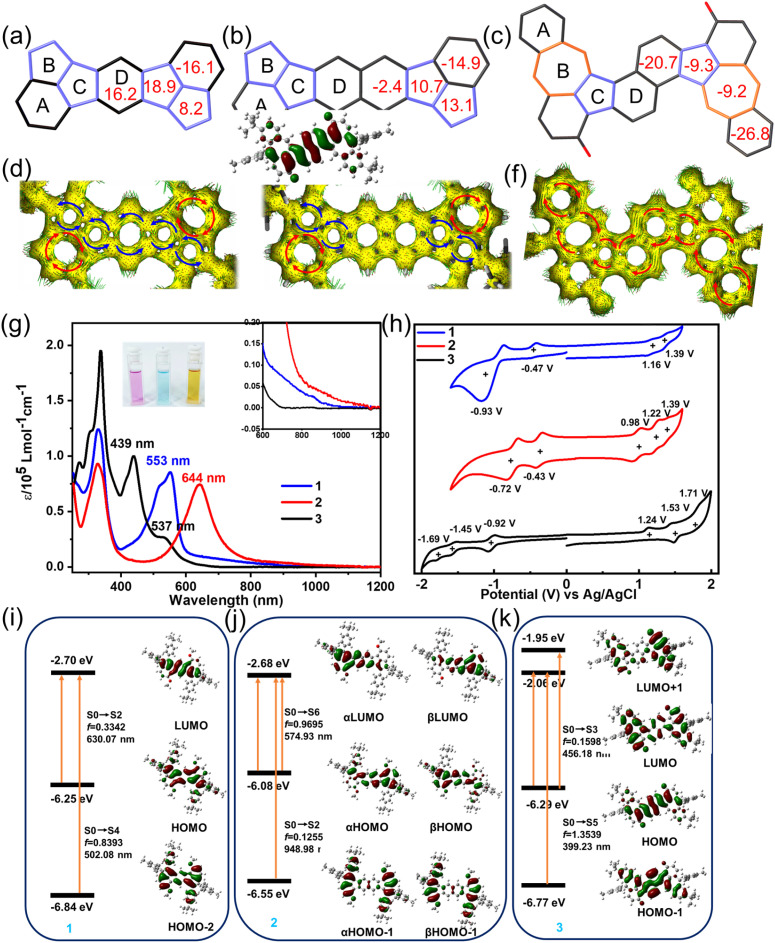
The calculated NICS(1)_*zz*_ values at 1 Å of *Z* axis of (a) 1, (b) 2 and (c) 3. The calculated ACID plots of (d) 1, (e) 2 and (f) 3. The clockwise ring current represents aromaticity and the counter-clockwise ring current represents antiaromaticity. (g) The UV-vis absorption spectra of 1, 2 and 3 (10^−5^ mol L^−1^ in DCM solution). (h) Cyclic voltammograms (CV) curves of 1, 2 and 3. The calculated absorption transitions of (i) 1, (j) 2 and (k) 3 based on TD-DFT calculations. (i) and (k) were based on M06-2X/6-311G(d,p) level of theory with closed-shell single state. (j) Was based on UM06-2X/6-311G(d,p) level of theory with open-shell singlet state.

To gain deeper insights into the electronic structures and aromaticity of 1, 2 and 3, nucleus-independent chemical shift (NICS) calculations were performed at the (U)B3LYP/6-31G(d,p) level of theory. The obtained NICS(1)_*zz*_ values of 1 are −16.1, 8.2, 18.9 and 16.2 ppm for rings A, B, C and D (Fig. 2a and S13), respectively, indicating that rings B, C, and D in 1 are strongly antiaromatic. Similarly, NICS(1)_*zz*_ values of −14.9, 13.1, 10.7 and −2.4 ppm for rings A, B, C and D were observed in 2 (Fig. 2b and S13), suggesting rings B and C in 2 are also antiaromatic, while ring D is weak aromaticity. The reduced antiaromaticity of 2 compared to 1 correlates with its enhanced open-shell character. In contrast, the NICS values of B, C and D rings is smaller than the individual *s*-indacene (54.7, 45.0 and 54.7 ppm) and pentalene (62.4 and 62.4 ppm) motifs (see Fig. S17), indicating the B, C and D rings in 1 and 2 are hybridizing structures, which weakens the *s*-indacene and pentalene motifs and thus improves the stability of these two molecules. To further explore the global antiaromaticity of compounds 1 and 2, we also calculated the bis(pentalene) derivatives without the A ring. As shown in Fig. S18, the NICS values of two five-membered rings are 38.8 and 37.6 ppm for bis(pentalene)-fuse benzene (compound A) and 31.9 and 29.2 ppm for bis(pentalene)-fuse naphthene (compound B), while the central benzene or naphthene are weak antiaromaticity or aromaticity (7.4 ppm for A and −2.1 ppm for B), indicating that the two pentalene motifs in these molecules were dependent and the antiaromatic electronic property is more localized owing to the central aromatic rings.^55^ In contrast, the aromaticity of the central benzene or naphthalene rings of 1 and 2 was broken, showing quinoidal structure and antiaromaticity characteristics. Consequently, 1 and 2 exhibit global antiaromatic character. Conversely, the rings A and D of 3 exhibit strong aromatic features with NICS(1)_*zz*_ values of −26.8 and −20.7 ppm for rings A and D and the rings B and C of 5/7 motif are weak aromaticity with NICS(1)_*zz*_ values of −9.2 and −9.3 ppm (Fig. 2c), indicating 5/7 motif in 3 is not independent because of the good delocalized characteristic upon the whole skeleton, aligning well with the bond analysis. Additionally, these results are consistent with the anisotropy of the induced current density (ACID) and isotropic chemical shielding surface (ICSS) analysis results. As illustrated in Fig. 2d and e, the calculated ACID plot shows counter-clockwise ring current along the periphery except for ring A of 1 and 2, corresponding to the antiaromaticity of indacene and pentalene. On the contrary, diamagnetic ring currents are found along the periphery of 3 (Fig. 2f), indicating global aromaticity along the periphery. These results agree well with the bond length analysis in the crystals and the ICSS calculation (Fig. S12).

The UV-vis absorption spectra of 1, 2 and 3 in dichloromethane (DCM) solution at a concentration of 10^−5^ mol L^−1^ are shown in Fig. 2g. The colors of 1, 2 and 3 in DCM are purple, blue and claybank, respectively, as highlighted in the inset of Fig. 2g. 1 and 2 display similar absorption patterns with two main absorption peaks based on their likely electronic structures. The DCM solution of 1 exhibits a prominent long-wavelength absorption peak at 553 nm, accompanied by a weak absorption tail extending up to 907 nm. Time-dependent (TD) DFT calculations reveal that the absorption band at 553 nm with a shoulder peak at 521 nm is mainly attributed to HOMO−2 → LUMO (S_0_ → S_4_) electronic transition. The weak-tail absorption is attributed to symmetry-forbidden HOMO → LUMO electronic transition with an oscillator strength of 0.3342 (Fig. 2i, S22a and Table S11), following its antiaromatic character. For 2, the absorption spectrum exhibits a significant bathochromic shift compared to 1. The long-wavelength absorption peak appears at 644 nm, red-shifted by 91 nm, which is mainly ascribed to α(β)HOMO → α(β)LUMO (S_0_ → S_6_) based on an alternative functional of UM06-2X. The long-tail absorption also extends to 1071 nm, which is attributed to α(β)HOMO → α(β)LUMO and α(β)HOMO−1 → α(β)LUMO (S_0_ → S_2_) with an oscillator strength of 0.12550 (Fig. 2j, S22b and Table S12). For compound 3, the absorption peak at 439 nm is mainly attributed to the HOMO → LUMO and HOMO → LUMO+1 (S_0_ → S_3_) electronic transition and the absorption band at 338 nm with a shoulder absorption originates from HOMO−1 → LUMO (S_0_ → S_5_) electronic transitions (Fig. 2k, S22d and Table S14). Accordingly, the optical energy gaps (*E*^opt^_g_) for 1, 2 and 3 were determined to be 1.36, 1.15, and 2.06 eV, respectively, from the onset of their UV-vis absorption spectra. Furthermore, time-dependent UV-vis measurements were performed under ambient conditions to investigate the stability of 1, 2 and 3 (see Fig. S23 and S24). The results revealed that 1 and 3 exhibited exceptional stability, with no significant changes observed in their absorption spectra in DCM solution within 28 days. In contrast, 2 showed gradual degradation when exposed to ambient conditions, with a fitted half-life of approximately 32 days for a 10^−5^ M DCM solution. This difference in stability can be attributed to the more pronounced open-shell character of 2, which renders it more reactive under ambient conditions. In addition, three compounds can be stored as crystalline solids under ambient conditions without any degradation. These results highlight the remarkable stability of these non-benzenoid PAHs, particularly for 1 and 3, which maintain their structural and optical integrity even in solution over extended periods. Thermogravimetry analysis (TGA, Fig. S10) demonstrated that 2 displays a notably lower onset decomposition temperature (128 °C) than 1 (144 °C), which is attributed to the more obvious open-shell electronic character of 2. In contrast, compound 3 exhibits a higher thermal decomposition temperature over 370 °C, which is rationalized by the global aromaticity of 3.

To further explore the typical diradical properties, the photoluminescence and photothermal conversion were measured. As shown in Fig. S25, no significant NIR luminescence was detected with a very weak emission peak at 783 nm for 2. Similarly, compound 1 also shows weak luminescence with the maximum emission peak at 740 nm. The weak luminescence may be attributed to the efficient non-radiative decay pathways of two compounds. So, we further evaluated the photothermal conversion of the solid samples (*ca.* 7 mg) under irradiation with an 808 nm laser at different power levels. The temperature increases obviously with increasing the light power (Fig. S26). Notably, the heating rate of compound 2 is significantly higher than that of compound 1, which is consistent with its longer absorption wavelength and more pronounced open-shell diradical character. Upon irradiation at a power of 0.8 W cm^−2^ for approximately 5 s, the temperature of compound 1 reached over 180 °C, while that of compound 2 reached up to 260 °C, demonstrating excellent photothermal conversion properties.

The electrochemical behaviors of 1, 2 and 3 were investigated by cyclic voltammetry (CV) in anhydrous DCM solution with ferrocene/ferrocenium (Fc/Fc^+^) as an external standard, revealing distinct redox behaviors for each compound (Fig. 2h). 1 exhibited two oxidation waves at *E*^ox^_1/2_ = 1.16 and 1.39 V *versus* Fc/Fc^+^, along with two reduction waves at *E*^re^_1/2_ = −0.47 and −0.93 V, while 2 showed more complex redox activity with three oxidation peaks at 0.98, 1.22, and 1.39 V and two reduction peaks at −0.43 and −0.72 V. In contrast, 3 displayed oxidation potentials at 1.24, 1.53, and 1.71 V and reduction potentials at −0.92, −1.45, and −1.69 V, reflecting its different electronic structure. From the first redox couples, we estimated the highest occupied molecular orbital (HOMO)/lowest unoccupied molecular orbital (LUMO) energy levels at −5.48/−3.85 eV for 1, −5.30/−3.89 eV for 2, and −5.56/−3.40 eV for 3, yielding electrochemical band gaps (*E*^el^_g_) of 1.63, 1.41, and 2.16 eV, respectively (Table S15). Considering the reversible oxidation and reduction peaks of 1 and 2, we performed chemical oxidation titration experiments with NO·SbF_6_. As shown in Fig. S27, the absorption intensity gradually decreases with the addition of the NO·SbF_6_. No new absorption peaks belonging to 1(2)^+˙^ or 1(2)^2+^ species appeared, indicating that 1 and 2 decomposed under the oxidant condition.

### Diradical and charge transport characteristics

**Fig. 3:**
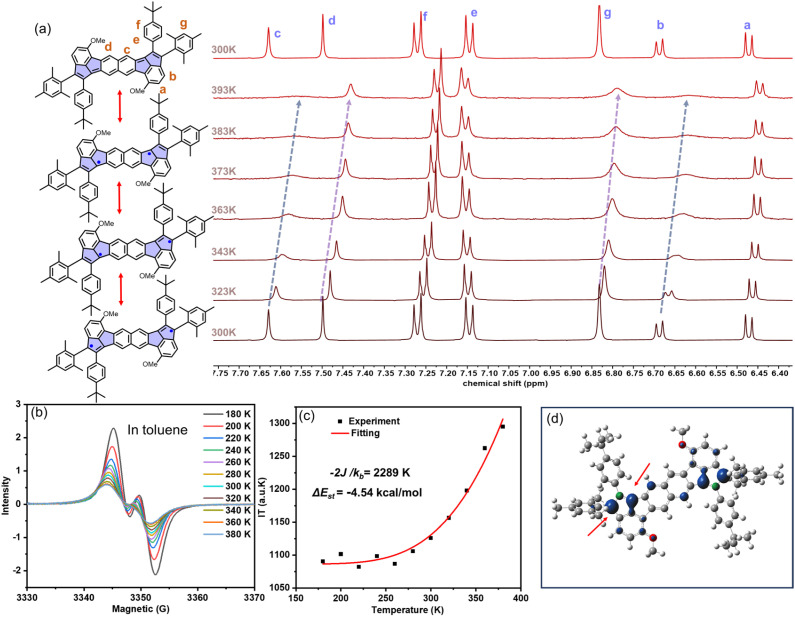
(a) The resonance structures of 2 with open-shell diradical and close-shell quinoid structure (left) and the VT ^1^H NMR of 2 from 300 to 393 K in C_2_D_2_Cl_4_ (right). (b) Temperature dependence of the ESR signals of 2 in toluene solution. (c) The integrated signal multiplied by temperature and fitted with the Bleaney–Bowers equation of 2. (d) Spin density surfaces calculated by DFT UB3LYP-D3(BJ)/6-31G(d,p) in the open-shell triplet state. The isovalue is 0.005.

The chemical and electronic structures of 1, 2 and 3 were further investigated by variable-temperature (VT) ^1^H NMR measurements. For 2, the proton signals in C_2_D_2_Cl_4_ exhibited significant broadening with the temperature increasing from 300 to 393 K, particularly for the protons b, c, d and g located on the backbone (see Fig. 3a and S29). Upon cooling back to 300 K, the signals fully recovered to their original intensity. However, when further cooled to 243 K, the intensity of the signals gradually decreased, which was attributed to the reduced solubility of 2 at lower temperatures (Fig. S30). This reversible thermal broadening strongly indicates population of a triplet state at elevated temperatures, consistent with the significant open-shell diradical character of 2. Meanwhile, both 1 and 3 maintained sharp NMR signals throughout the same temperature range (300–393 K) (Fig. S28 and S31), demonstrating their closed-shell nature with negligible thermally accessible diradical states. We also tested the VT-NMR of 1 and 2 in C_6_D_4_Cl_2_ (298–393 K) and the corresponding results are shown in Fig. S32 and S33, which are consistent with the VT-NMR results by using C_2_D_2_Cl_4_. Consequently, the proton signals of compound 2, exhibiting significant broadening than that of 1, can be attributed to the stronger open-shell property rather than the solvent effect.

Complementary electron-spin resonance (ESR) and superconducting quantum interference device (SQUID) measurements provided definitive evidence for the distinct open-shell characteristics of two antiaromatic compounds of 1 and 2. While 1 showed a negligible solution-phase ESR signal but weak solid-state signals attributed to its small band gap (Fig. S34a),^90^2 exhibited strong isotropic signals (*g* = 2.002) in both toluene solution and the solid state, unambiguously confirming its paramagnetic nature and carbon-centered radical character (Fig. S34b). Additionally, VT-ESR studies (180–380 K in toluene) demonstrated increasing signal intensity with decreasing temperature for 2 (Fig. 3b and S35–S36), with Bleaney–Bowers analysis (the signal (*I*) × *T versus T*) yielding Δ*E*_st_ = −4.54 kcal mol^−1^ for 2 (Fig. 3c).^91–94^ DFT calculations at the UCAM-B3LYP/6-31G(d) level reveal 2 displays an open-shell singlet ground state, with a *y*_0_ of 0.38, while 1, in contrast, exhibits a negligible diradical character with a closed-shell state (Table S16), consistent with the more pronounced diradical character of 2. The hybridizing structure of *s*-indacene and pentalene allows the spin densities in 2 to be primarily localized both on C7 and C9 of the cyclopenta-rings, which is conducive to improving *y*_0_ and stability (see Fig. 3d and S38). This electronic structure property is completely different from the reported individual *s*-indacene or pentalene structures, indicating the hybridization of the antiaromatic motifs is an effective strategy to modulate the whole aromaticity and diradical as well as molecular stability. The spin-density distribution also corresponds to the significant changes observed in the VT-NMR signals of b, c, and g hydrogens (Fig. 3a). Additionally, SQUID magnetometry further corroborated these findings, showing a continuous increase in *χ*_m_*T* for 2 from 2–300 K, indicative of progressive thermal population of triplet states, whereas 1 displayed only weak, discontinuous magnetic responses characteristic owing to its close-shell structure (Fig. S37).

**Fig. 4:**
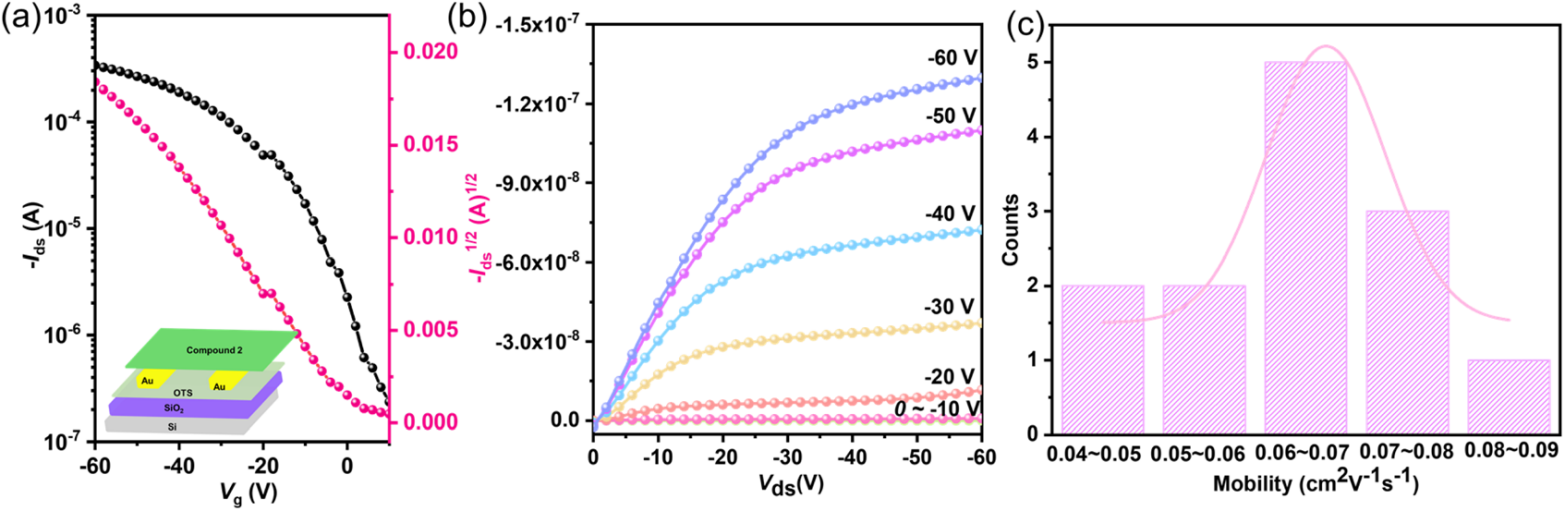
(a) Transfer characteristics of OFETs based on 2 (the inset is the BGBC OFET device structure). (b) Typical output curve of OFETs based on 2. The width (*W*) and length (*L*) of the conductive channel are 1440 and 5 µm, respectively. (c) The mobility distribution of 13 FET devices based on 2.

The charge transport characteristics of 1, 2, and 3 were evaluated through bottom-gate bottom-contact (BGBC) field-effect transistor (FET) devices fabricated under ambient conditions, with detailed device architecture illustrated in Fig. 4a and fabrication procedures provided in the SI. While 1 showed negligible charge transport behavior, 2 demonstrated well-defined p-type semiconductor characteristics, as evidenced by the transfer and output curves (Fig. 4a and b). Analysis of the saturation regime transfer characteristics revealed impressive hole mobilities for 2, with maximum and average values reaching 0.083 and 0.064 cm^2^ V^−1^ s^−1^, respectively, accompanied by an on/off current ratio exceeding 10^3^ (Fig. 4c and Table S17). These values are comparable to the best-reported values for organic diradical small molecules.^95,96^ The good carrier-transporting properties may be attributed to the good planarity, tight molecular packing, better diradical stability, and suitable frontier orbital energy levels. However, charge carrier mobility decreased dramatically with increasing annealing temperature, from 0.083 cm^2^ V^−1^ s^−1^ at rt to 0.0025 cm^2^ V^−1^ s^−1^ at 50 °C and 0.000064 cm^2^ V^−1^ s^−1^ at 80 °C (Fig. S39 and Table S17), which may be attributed to the instability caused by the thermally accessible triplet diradical.

## Conclusions

In summary, we have developed a modular synthetic strategy based on a sterically controlled 5-*exo*-dig cyclization that enables the construction of stable PAHs featuring indacene, pentalene, and azulene motifs. This approach yielded three distinct compounds with finely tunable electronic structures. Compounds 1 and 2 combine antiaromatic *s*-indacene and pentalene units within a single molecule, exhibiting global antiaromaticity, with 2 displaying a pronounced open-shell diradical character, with a *y*_0_ of 0.38 and a small Δ*E*_st_ of −4.54 kcal mol^−1^ compared to 1, while compound 3 adopts a twisted S-shaped geometry with global aromaticity and a closed-shell configuration. Notably, 2 demonstrates promising p-type semiconductor behavior with a hole mobility up to 0.083 cm^2^ V^−1^ s^−1^, and all three compounds exhibit remarkable ambient stability. This work provides a versatile and controllable approach to access structurally diverse non-benzenoid PAHs containing indacene, pentalene, or azulene motifs with tunable (anti)aromaticity and diradical character for organic electronics applications.

## Author contributions

Liangliang Chen: conceptualization, investigation, project administration, writing – original draft. Zhichun Shangguan: calculation, writing – review & editing. Tianyu Shi: investigation, synthesis. Liyuan Qin: single-crystal analysis. Yiyun Zeng, Qingqiu Zhu, Jin Chen, Junhong Liang, Wentao Miao, Yurong He, Xiaosong Qiu and Xunchang Wang: review & editing. Deqing Zhang: supervision. Renqiang Yang: funding acquisition, supervision, writing – review & editing.

## Conflicts of interest

There are no conflicts to declare.

## Supplementary Material

SC-OLF-D6SC00121A-s001

SC-OLF-D6SC00121A-s002

## Data Availability

CCDC 2478865–2478870 contain the supplementary crystallographic data for this paper.^97*a*–*f*^ All experimental procedures and data related to this study can be found in the supplementary information (SI). Supplementary information: experimental details, synthesis, NMR spectra, HRMS, computational details, crystallographic information, and additional characterization data. See DOI: https://doi.org/10.1039/d6sc00121a.
