# Correction: Sterically controlled 5-*exo*-dig cyclization enables synthesis of non-benzenoid polycyclic aromatic hydrocarbons with intriguing (anti)aromaticity and diradical properties

**DOI:** 10.1039/d6sc90125e

**Published:** 2026-06-19

**Authors:** Liangliang Chen, Zhichun Shangguan, Tianyu Shi, Liyuan Qin, Yiyun Zeng, Qingqiu Zhu, Jin Chen, Junhong Liang, Wentao Miao, Yurong He, Xiaosong Qiu, Xunchang Wang, Deqing Zhang, Renqiang Yang

**Affiliations:** a Key Laboratory of Flexible Optoelectronic Materials and Technology (Ministry of Education), School of Optoelectronic Materials & Technology, Jianghan University Wuhan 430056 China chenliangliang@jhun.edu.cn yangrq@jhun.edu.cn; b College of Chemistry and Materials Engineering, Wenzhou University, Key Lab of Biohealth Materials and Chemistry of Wenzhou Wenzhou 325027 China; c Beijing National Laboratory for Molecular Sciences, Institute of Chemistry, Chinese Academy of Sciences Beijing 100190 China

## Abstract

Correction for ‘Sterically controlled 5-*exo*-dig cyclization enables synthesis of non-benzenoid polycyclic aromatic hydrocarbons with intriguing (anti)aromaticity and diradical properties’ by Liangliang Chen *et al.*, *Chem. Sci.*, 2026, https://doi.org/10.1039/d6sc00121a.

The authors regret that due to an error in figure assembly part of Fig. 2b was obscured in the final version of the manuscript. The corrected Fig. 2 is shown below. This does not alter the interpretations or conclusions of the paper.

**Fig. 1:**
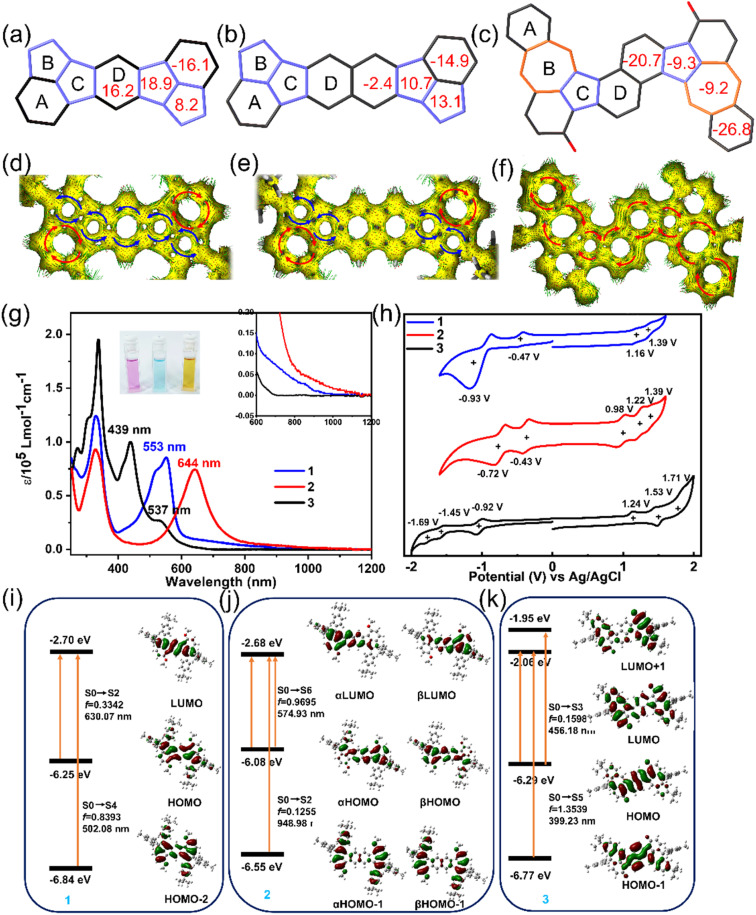
The calculated NICS(1)_*zz*_ values at 1 Å of *Z* axis of (a) **1**, (b) **2** and (c) **3**. The calculated ACID plots of (d) **1**, (e) **2** and (f) **3**. The clockwise ring current represents aromaticity and the counter-clockwise ring current represents antiaromaticity. (g) The UV-vis absorption spectra of **1**, **2** and **3** (10^−5^ mol L^−1^ in DCM solution). (h) Cyclic voltammograms (CV) curves of **1**, **2** and **3**. The calculated absorption transitions of (i) **1**, (j) **2** and (k) **3** based on TD-DFT calculations. (i) and (k) were based on M06-2X/6-311G(d,p) level of theory with closed-shell single state. (j) was based on UM06-2X/6-311G(d,p) level of theory with open-shell singlet state.

The Royal Society of Chemistry apologises for these errors and any consequent inconvenience to authors and readers.

