# Supplementary material for: Sterically controlled 5-exo-dig cyclization enables synthesis of non-benzenoid polycyclic aromatic hydrocarbons with intriguing (anti)aromaticity and diradical properties
Source: Chem Sci. 2026 May 11;17(25):12346–57. doi: 10.1039/d6sc00121a (PMC13158692; doi:10.1039/d6sc00121a)
Supplement: SC-017-D6SC00121A-s001 [file SC-017-D6SC00121A-s001.pdf]

Supplementary Information (SI) for Chemical Science.
This journal is © The Royal Society of Chemistry 2026

Sterically Controlled 5-exo-dig Cyclization Enables Synthesis of Non-benzenoid Polycyclic Aromatic Hydrocarbons with Intriguing (Anti)aromaticity and Diradical

Liangliang Chen,[†,*,1] Zhichun Shangguan,[†,2] Tianyu Shi,[†,3] Liyuan Qin,[†,3] Yiyun Zeng,[1] Qinqiu Zhu,[[1] Jin Chen,[1] Junhong Liang,[1] Wentao Miao,[1] Yurong He,[1], Xiaosong Qiu,[1] Xunchang Wang,[1] Deqing Zhang,[3] Renqiang Yang*,[1]

1 Key Laboratory of Flexible Optoelectronic Materials and Technology (Ministry of Education), School of Optoelectronic Materials & Technology, Jianghan University, Wuhan, 430056, China

2 College of Chemistry and Materials Engineering, Wenzhou University, Key Lab of Biohealth Materials and Chemistry of Wenzhou, Wenzhou, 325027, China.

3 Beijing National Laboratory for Molecular Sciences, Institute of Chemistry, Chinese Academy of Sciences, Beijing, 100190, China.

[†] These authors contribute equally to this work.

CONTENTS

1. Materials and characterization techniques 2
2. Synthesis and characterization 4
3. The ^{1}H-^{1}H NOESY of compounds....20
4. Crystallographic data 23
5. Thermogravimetric analysis (TGA) analysis 32
6. Infrared spectra analysis....32
7. Calculations on electronic properties 33
8. The stabilities of 1, 2 and 3 52
9. The photoluminescence and photothermal conversion of compound 1 and 2 53
10. The oxidation titration experiments 54
11. The variable-temperature ^{1}H NMR....55
12. The magnetic properties....58
13. OFETs fabrication and characterization....61
14. Atomic Cartesian coordinates of the optimized geometry 63
15. ^{1}H NMR and ^{13}C NMR spectra 73
16. HRMS spectra 88
17. Reference....95

1. Materials and characterization techniques

All reagents and starting materials were purchased from commercial suppliers and used without further purification unless otherwise specified.

^{1}H NMR and ^{13}C NMR spectra were recorded on AVANCE III 400 MHz, AVANCE III 500 MHz 600MHz and Bruker NEO 700 WB spectrometers. High-resolution mass spectral (HRMS) data were collected on either 9.4T Solarix Mass instrument or APEX II Mass instrument. Elemental analyses (EA) were performed on a Carlo-Erba-1106 instrument. Thermogravimetric analysis (TGA) analysis was carried on a PerkinElmer TGA 8000 Thermogravimetric Analyzer instrument under N_2 at a heating rate of 10 °C/min from 50 °C to 600 °C. UV-vis-NIR absorption spectra were collected on a HITACHI UH4150 UV-vis spectrophotometer. Cyclic voltammograms (CV) and differential pulse voltammograms (DPV) measurements were carried out in a conventional three-electrode cell using a glassy carbon as the working electrode, Pt as the counter electrode, and Ag/AgCl (saturated KCl) as the reference electrode on a computer-controlled CHI660C instrument at ambient temperature; n-Bu_4NPF_6 (0.1 M in dichloromethane) was used as the supporting electrolyte. For calibration, the redox potential of ferrocene/ferrocenium (Fc/Fc^{+}) was measured under the same conditions. HOMO and LUMO energies of these conjugated polymers were estimated with the following equations: HOMO = - (E_{ox}^{onset} -$E_{Fc}^{1/2}$ + 4.8) eV, LUMO = -(E_{red}^{onset} -$E_{Fc}^{1/2}$ + 4.8) eV. The single-crystal X-ray diffraction was carried out on a Rigaku Synergy-R diffractometer. XRD measurements were conducted on a PANalytical Empyrean with X-ray wavelength of 1.54 Å. The electron-spin resonance (ESR) spectra of solution and solid were conducted on Bruker E 500. The superconducting quantum interference device (SQUID) magnetometry was measured on MPMS3. The FET device performances were evaluated on a Keithley 4200 SCS semiconductor parameter analyzer.

Density functional theory (DFT) calculations were employed with the Gaussian 09 package.[S1] The geometry optimization and frequency analysis were calculated at the (U)B3LYP-D3(BJ)/6-31G(d,p) level of theory.[S2-S4] The vertical electronic

excitation energies were calculated using time-dependent density functional theory (TDDFT)[S5], and 30 lowest-lying singlet excited states were considered at (U)B3LYP-D3(BJ)/6-31G(d,p) level of theory. Nucleus-independent chemical shifts (NICS)[S6], the anisotropy of current-induced density (ACID)[S7], and the iso-chemical shielding surfaces (ICSS)[S8] were calculated at the (U)B3LYP/6-31G(d,p) level of theory, with the assistance of the Multiwfn program[S9]. The diradical character (y_0) was calculated according to following equation[S10]:

$$y_0 = 1 - \frac{2T}{1 + T^2}$$

where T is defined as:

$$T = \frac{n_{HOMO} - n_{LUMO}}{2}$$

Here, n_{HOMO} and n_{LUMO} represent the occupation numbers of the HOMO and LUMO orbitals, respectively.

2. Synthesis and characterization

2.1 *Synthesis and characterization of compound **1***

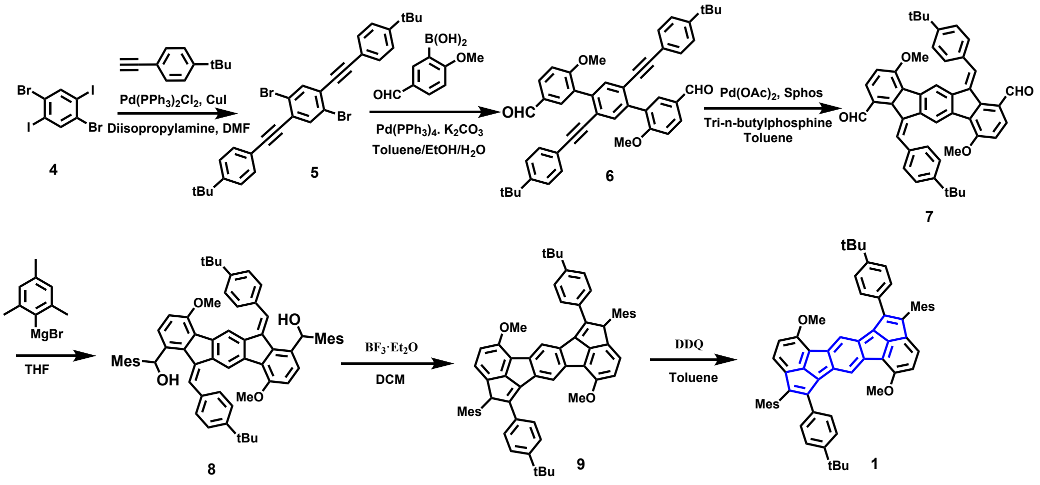

Scheme S1 The synthetic routes of compound **1**

*Synthesis of compound **5***

To a 100 mL two-necked flask equipped with a magnetic stir bar, 1,4-Dibromo-2,5-diiodobenzene (compound **4**, 3 g, 6.15 mmol), $Pd(PPh_3)_2Cl_2$ (440 mg, 0.62 mmol) and CuI (240 mg, 1.24 mmol) were added. The mixture was degassed and charged with nitrogen for three times and then 1-(tert-butyl)-4-ethynylbenzene (1.943 g, 12.3 mmol), diisopropylamine (20 mL) and DMF (20 mL) were added subsequently. The mixture was stirred at room temperature overnight. The reaction was quenched with hydrochloric acid solution (2 M, 60 mL), and the mixture was extracted with dichloromethane (100 mL×3) and washed with hydrochloric acid solution (2 M, 60 mL×3). The organic layer was dried by anhydrous Na_2SO_4 and concentrated under reduced pressure. The crude product was then purified by flash column chromatography using petroleum ether (b.p. 60-90 ^{o}C) as eluent to give compound **5** as a white solid (2.7 g, 80.1% yield).

^{1}H NMR (400 MHz, $CDCl_3$, 298 K) δ (ppm) 7.77 (s, 2H), 7.51 (d, J = 8.4 Hz, 4H), 7.39 (d, J = 8.4 Hz, 4H), 1.33 (s, 18H).

^{13}C NMR (101 MHz, $CDCl_3$, 298 K) δ (ppm) 152.6, 135.9, 131.6, 126.5, 125.5, 123.7, 119.3, 96.9, 86.4, 34.9, 31.2.

HRMS (MALDI- FTICR) m/z: $[M]^+$ calcd for $C_{30}H_{28}Br_2$ 546.0552; found 546.0557; err (ppm) -1.0.

*Synthesis of compound **6***

To a 250 mL three-necked flask equipped with a magnetic stir bar, compound **5** (1.5 g, 2.75 mmol), 2-Methoxy-5-formylphenylboronic acid (1.98 g, 11mmol), Na_2CO_3 (1.75 g, 16.5 mmol) and $Pd(PPh_3)_4$ (318 mg, 0.275 mmol) were added. The mixture was degassed and charged with nitrogen for three times. Then toluene (80 mL), ethanol (15 mL) and water (15 mL) were added into the flask. The mixture was heated at 110 ^{o}C (oil bath) for 15 h. After cooling to room temperature, the solvent was removed by rotary evaporation. The crude product was then purified by flash column chromatography using petroleum ether (b.p. 60-90 ^{o}C) and dichloromethane (2/1, v/v) as eluent to give compound **6** as a light-yellow solid. (1.58 g, 87.3% yield).

^{1}H NMR (400 MHz, $CDCl_3$, 298 K) δ (ppm) 9.97 (s, 2H), 8.01-7.96 (m, 4H), 7.65 (s, 2H), 7.27 (d, *J* = 8.5 Hz, 4H), 7.15 (d, *J* = 9.1 Hz, 2H), 7.11 (d, *J* = 8.5 Hz, 4H), 3.92 (s, 6H), 1.28 (s, 18H).

^{13}C NMR (101 MHz, $CDCl_3$) δ 190.8, 162.1, 151.7, 138.9, 133.7, 133.5, 131.5, 131.1, 129.7, 129.5, 125.3, 123.1, 120.0, 111.0, 94.0, 88.0, 56.1, 34.8, 31.1.

HRMS (MALDI- FTICR) m/z: $[M]^+$ calcd for $C_{46}H_{42}O_4$ 658.3077; found 658.3076; err (ppm) -0.2.

Synthesis of compound 7

To a Pyrex tube equipped with a magnetic stir bar, compound **6** (50 mg×25, 1.82 mmol), $Pd(OAc)_2$ (2.2 mg×24, 0.23 mmol), 2-dicyclohexylphosphino-2',6'-dimethoxybiphenyl (Sphos, 6.8 mg×24, 0.41 mmol), Tri-n-butylphosphine (20 μL×24, 1.92 mmol) and toluene (2 mL×24) were added sequentially. Then through a freeze-pump-thaw cycle, the tube was charged with nitrogen for three times again. After rising to room temperature, the mixture was heated at 120 °C for 24 h. After cooling to room temperature, a large amount of precipitation was generated and filtered off. The precipitation was washed with MeOH. The residue was collected and dried under vacuum to afford compound **7** (985 mg, 82.1%), which was directly used for the next step without further purification.

^{1}H NMR (400 MHz, $CDCl_3$, 298 K) δ (ppm) 10.37 (s, 2H), 8.70 (s, 2H), 8.54 (s, 2H), 7.79 (d, *J* = 8.5 Hz, 2H), 7.72 -7.66 (m, 4H), 7.57-7.52 (m, 4H), 6.92 (d, *J* = 8.6 Hz, 2H), 3.83 (s, 6H), 1.43 (s, 18H).

^{13}C NMR (176 MHz, $CDCl_3$, 298 K) δ (ppm) 190.8, 159.2, 151.4, 143.2, 137.2, 137.1, 136.3, 136.0, 134.1, 133.8, 130.0, 128.6, 126.8, 125.4, 119.0, 109.4, 55.5, 34.9, 31.5.

HRMS (MALDI- FTICR) m/z: $[M]^+$ calcd for $C_{46}H_{42}O_4$ 658.3077; found 658.3080; err (ppm) 0.4.

*Synthesis of compound **9***

First step: To a 100 mL three-necked flask equipped with a magnetic stir bar, compound **7** (250 mg, 0.38 mmol) and THF (30 mL) were added. The mixture was degassed and charged with nitrogen for three times and stirred at 0 °C (ice-water bath) for 5 min. Then, 2-mesitylmagnesium bromide (1 M solution in THF, 3.8 mL, 3.8 mmol) was added to the mixture at 0 °C. After dripping completely, the mixture was warmed to room temperature for 2 h. Then, H_2O was added to quench the reaction. The residual was extracted by ethyl acetate three times (50 mL×3), and washed with saturated sodium chloride aqueous solution (50 mL×3). The organic layer was dried by anhydrous Na_2SO_4. The crude product of compound **8** was obtained by the removal of solvent and used directly for the next step.

HRMS (MALDI- FTICR) m/z: $[M]^+$ calcd for $C_{64}H_{66}O_4$ 898.4955; found 898.4960; err (ppm) -0.5.

Second step: A 100 mL three-necked flask containing the obtained crude product was under vacuum for 15 min. Then, 20 mL of dry dichloromethane (DCM) was added, and 0.6 mL of $BF_3{\cdot}OEt_2$ was added dropwise through the syringe (turned dark immediately). The mixture was stirred for 5 h at room temperature. Then, 5 mL of saturated sodium carbonate (Na_2CO_3) solution was added to quench the reaction. The mixture was extracted by DCM (50 mL×3), and washed with saturated sodium chloride aqueous solution (50 mL×3). The organic layer was dried with anhydrous Na_2SO_4. The solvent was removed under reduced pressure and the residual, containing a small amount DCM solvent, was added to MeOH to precipitate the solid. The precipitation was filtered and the filter residue was washed with MeOH. After drying under the vacuum, the obtained orange crude product of compound **9** was directly used for the next reaction.

HRMS (MALDI- FTICR) m/z: $[M]^+$ calcd for $C_{64}H_{62}O_2$ 862.4744; found 862.4745; err (ppm) 0.1.

*Synthesis of compound **1***

To a 100 mL three-necked flask equipped with a magnetic stir bar, compound **9** (crude product obtained from the above-mentioned two steps) and 20 mL dry toluene were added. The mixture was degassed and charged with nitrogen for three times and stirred at 0 °C (ice-water bath) for 3 min. Then, 2,3-dichloro-5,6-dicyano-1,4-benzoquinone (DDQ) (250 mg, 1.1 mmol) in 5 mL dry toluene was added to the mixture and the mixture was stirred under N_2 at room temperature for 1 h (the color of the mixture turned to red-brown). Then H_2O was added to quench the reaction. The mixture was extracted by DCM (50 mL×3), and washed with saturated sodium chloride aqueous solution (50 mL×3). The organic layer was dried by anhydrous Na_2SO_4. The crude product was further purified by a short silica gel column (PE/DCM = 1/1, v/v). Compound **1** was obtained as an army green solid (57 mg, 17.3% yield in three steps).

^{1}H NMR (700 MHz, $C_2D_2Cl_4$, 298 K) δ (ppm) 7.55 (s, 2H), 7.24 (d, *J* = 8.5 Hz, 4H), 7.14 (d, *J* = 8.4 Hz, 4H), 6.84 (s, 4H), 6.63 (d, *J* = 7.6 Hz, 2H), 6.44 (d, *J* = 7.7 Hz, 2H), 3.79 (s, 6H), 2.28 (s, 6H), 2.02 (s, 12H), 1.26 (s, 18H).

^{13}C NMR (176 MHz, $C_2D_2Cl_4$, 298 K) δ (ppm) 155.5, 153.7, 153.3, 149.9, 147.5, 145.4, 137.3, 136.3, 133.7, 132.9, 132.4, 132.1, 128.5, 128.0, 127.3, 125.8, 125.3, 121.8, 116.5, 110.6, 56.0, 34.6, 31.5, 21.5, 20.5.

HRMS (MALDI- FTICR) m/z: $[M]^+$ calcd for $C_{64}H_{60}O_2$ 860.4587; found 860.4584; err (ppm) -0.4.

IR (ATR) 3018 cm^{-1} ($\nu_{C(sp^2)\text{-}H}$), 2923, 2850 cm^{-1} ($\nu_{C(sp^3)\text{-}H}$).

2.2 *Synthesis and characterization of compound* **2**

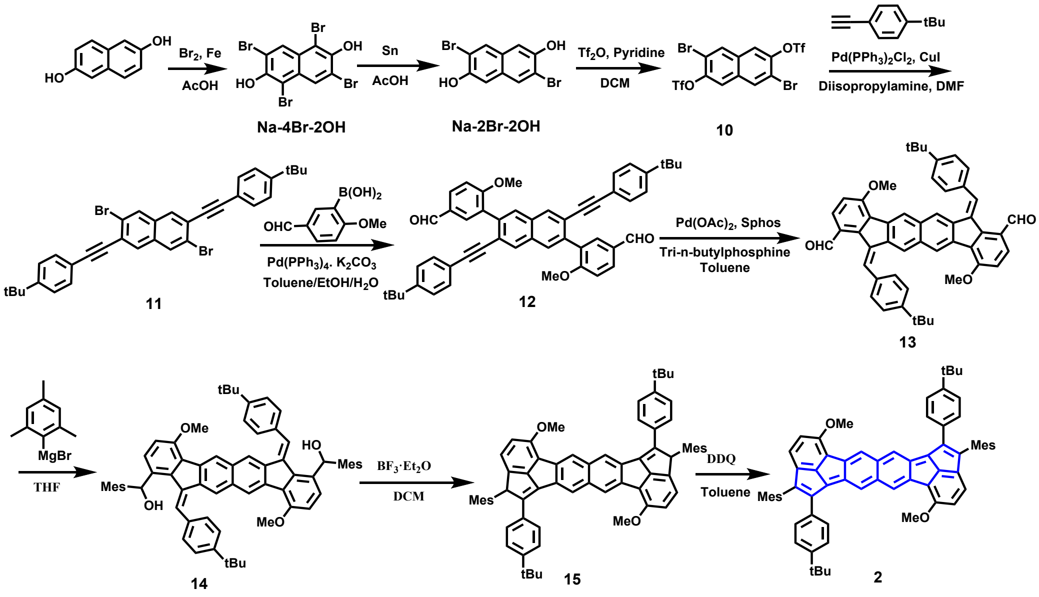

Scheme S2 The synthetic routes of compound **2**

Synthesis of compound ***Na-4Br-2OH***

In a 500 mL three-necked flask equipped with a magnetic stir bar, 2,6-dihydroxynaphthalene (10 g, 62.4 mmol) was dissolved in 250 mL of acetic acid. Elemental bromine (59.8 g, 374 mmol) was then slowly added and the dark orange solution was refluxed for 24 h. Then, iron dust (401.4 mg, 7.16 mmol) was added slowly. The mixture was refluxed for 92 h. After cooling to room temperature, 400 mL of water was added and the precipitate was collected by filtration, washed with acetone and dried under reduced pressure to afford compound ***Na-4Br-2OH*** as a gray solid (24 g, 80.9 % yield), which was directly used for the next step without further purification.

HRMS (MALDI- ESI) m/z: $[M]^+$ calcd for $C_{10}H_4O_2Br_4$ 475.6898; found 475.6897; err (ppm) 0.27.

Synthesis of compound ***Na-2Br-2OH***

In a 250 mL three-necked flask equipped with a magnetic stir bar, compound ***Na-4Br-2OH*** (5.0 g, 10.6 mmol) and tin (2.5 g, 21.2 mmol) were added. The mixture was degassed and charged with nitrogen for three times and AcOH (60 mL) was added. The mixture was heated at 120 ^oC for 48 h. After cooling to room temperature, the mixture

was filtered off. The filtrate was then poured into 300 mL H_2O and a large amount of precipitation was generated. The precipitation was washed with H_2O. The residue was collected and dried under vacuum to afford compound ***Na-2Br-2OH*** (1.83 g, 54.8% yield), which was directly used for the next step without further purification.

^{1}H NMR (400 MHz, DMSO-d_6, 298 K) δ (ppm) 10.34 (s, 2H), 7.99 (s, 2H), 7.18 (s, 2H).

^{13}C NMR (101 MHz, DMSO-d_6, 298 K) δ (ppm) 150.3, 130.3, 129.2, 114.1, 109.5.

HRMS (MALDI- ESI) m/z: $[M]^+$ calcd for $C_{10}H_6O_2Br_2$ 317.8709; found 317.8706; err (ppm) -0.74.

Synthesis of compound ***10***

To a 250 mL three-necked flask equipped with a magnetic stir bar, compound ***Na-2Br-2OH*** (5 g, 15.87 mmol), CH_2Cl_2 (250 mL) and pyridine (25 mL) were added. The mixture was degassed and charged with nitrogen for three times and then cooled to 0 ^{o}C (ice-water bath) for 3 min. Then, Trifluoromethanesulfonic anhydride (13.4 g, 47.6 mmol) was added slowly to the mixture under ice-water bath. After that, it was warmed to room temperature overnight. The reaction was quenched with hydrochloric acid solution (1 M, 200 mL), and the mixture was extracted with dichloromethane (200 mL×3) and washed with hydrochloric acid solution (1 M, 200 mL×3). The organic layer was dried with anhydrous Na_2SO_4 and concentrated under reduced pressure. The crude product was then purified by flash column chromatography using petroleum ether (b.p. 60-90 ^{o}C) and dichloromethane (8/1, v/v) as eluent to give compound **10** as a white solid (4.18 g, 45.5% yield).

^{1}H NMR (400 MHz, $CDCl_3$, 298 K) δ (ppm) 8.23 (s, 2H), 7.80 (s, 2H).

^{13}C NMR (101 MHz, $CDCl_3$, 298 K) δ (ppm) 145.7, 133.7, 131.8, 119.8, 118.7 (q, J = 322 Hz), 116.1.

HRMS (MALDI- ESI) m/z: $[M]^+$ calcd for $C_{12}H_4O_6Br_2F_6S_2$ 581.7694; found 581.7697; err (ppm) 0.71.

Synthesis of compound ***11***

To a 100 mL three-necked flask equipped with a magnetic stir bar, compound **10** (3 g, 5.18 mmol), $Pd(PPh_3)_2Cl_2$ (363 mg, 0.518 mmol) and CuI (198 mg, 1.036 mmol). The mixture was degassed and charged with nitrogen for three times and then 1-(tert-butyl)-4-ethynylbenzene (1.637 g, 10.36 mmol), diisopropylamine (30 mL) and DMF (30 mL) were added subsequently. The mixture was stirred at room temperature overnight. The reaction was quenched with hydrochloric acid solution (1 M, 100 mL), and the mixture was extracted with dichloromethane (100 mL×3) and washed with hydrochloric acid solution (1 M, 100 mL×3). The organic layer was dried with anhydrous Na_2SO_4 and concentrated under reduced pressure. The crude product was then purified by flash column chromatography using petroleum ether (b.p. 60-90 ^oC) and dichloromethane (10/1, v/v) as eluent to give compound **11** as a light-yellow solid (2.358 g, 76.4% yield).

^1H NMR (400 MHz, $CDCl_3$, 298 K) δ (ppm) 8.04 (s, 2H), 7.93 (s, 2H), 7.56 (d, J = 8.1 Hz, 4H), 7.41 (d, J = 8.4 Hz, 4H), 1.34 (s, 18H).

^{13}C NMR (101 MHz, $CDCl_3$, 298 K) δ (ppm) 152.4, 131.8, 131.6, 131.3, 130.5, 125.5, 124.6, 123.3, 119.7, 95.4, 87.5, 34.9, 31.2.

HRMS (MALDI- FTICR) m/z: $[M]^+$ calcd for $C_{34}H_{30}Br_2$ 596.0717; found 596.0708; err (ppm) -1.5.

Synthesis of compound ***12***

To a 100 mL three-necked flask equipped with a magnetic stir bar, compound **11** (2 g, 3.35 mmol), 2-methoxy-5-formylphenylboronic acid (1.81 g, 10 mmol), K_2CO_3 (2.311 g, 16.75 mmol) and $Pd(PPh_3)_4$ (194 mg, 0.167 mmol) were added. The mixture was degassed and charged with nitrogen for three times. Then toluene (80 mL), ethanol (15 mL) and water (15 mL) were added to the flask. The mixture was heated at 110 ^oC (oil bath) overnight. After cooling to room temperature, the solvent was removed by rotary evaporation. The crude product was then purified by flash column chromatography using petroleum ether (b.p. 60-90 ^oC) and dichloromethane (2/1, v/v)

to dichloromethane and ethyl acetate (20:1, v/v) as eluent to give a light-yellow solid **12** (1.227 g, 51.7% yield).

^{1}H NMR (400 MHz, $CDCl_3$, 298 K) δ (ppm) 9.99 (s, 2H), 8.11 (s, 2H), 8.02 (dt, *J* = 8.5, 1.7 Hz, 2H), 7.97 (t, *J* = 1.6 Hz, 2H), 7.83 (s, 2H), 7.28 (d, *J* = 7.6 Hz, 4H), 7.17 (d, *J* = 8.5 Hz, 2H), 7.12 (d, *J* = 8.6 Hz, 4H), 3.88 (s, 6H), 1.29 (s, 18H).

^{13}C NMR (101 MHz, $CDCl_3$, 298 K) δ (ppm) 191.0, 162.5, 151.6, 137.6, 133.4, 131.7, 131.5, 131.1, 130.6, 129.6, 128.4, 125.4, 122.6, 120.1, 110.9, 93.0, 88.4, 56.1, 34.8, 31.1.

HRMS (MALDI- FTICR) m/z: $[M]^+$ calcd for $C_{50}H_{44}O_4$ 708.3234; found 708.3239; err (ppm) 0.7.

*Synthesis of compound **13***

To a Pyrex tube equipped with a magnetic stir bar, compound **12** (50 mg × 22, 1.55 mmol), Pd(OAc)2 (2.2 mg × 22, 0.21 mmol), 2-dicyclohexylphosphino-2',6'-dimethoxybiphenyl (Sphos, 6.8 mg × 22, 0.36 μmol), Tri-n-butylphosphine (20 μL × 22, 1.76 mmol) and toluene (2 mL × 22) were added sequentially. Then through a freeze-pump-thaw cycle, the tube was charged with nitrogen for three times again. After raising to room temperature, the mixture was heated at 120 °C for 12 h. After cooling to room temperature, a large amount of precipitation was generated and filtered off. The precipitation was washed with MeOH. The residue was collected and dried under vacuum to afford compound **13** (952 mg, 86.5%), which was directly used for the next step without further purification.

^{1}H NMR (400 MHz, $CDCl_3$, 298 K) δ (ppm) 10.47 (s, 2H), 8.47 (s, 2H), 8.15 (s, 2H), 7.89 (d, *J* = 8.5 Hz, 2H), 7.79 (s, 2H), 7.57 (s, 8H), 7.04 (d, *J* = 8.6 Hz, 2H), 4.08 (s, 6H), 1.49 (s, 18H).

^{13}C NMR (176 MHz, $CDCl_3$, 298 K) δ (ppm) 190.7, 160.0, 151.6, 143.8, 136.5, 136.3, 136.0, 135.0, 134.7, 134.1, 132.4, 128.9, 128.5, 127.1, 126.4, 125.7, 123.7, 123.1, 109.7, 55.7, 34.9, 31.5.

HRMS (MALDI- FTICR) m/z: $[M]^+$ calcd for $C_{50}H_{44}O_4$ 708.3234; found 708.3233; err (ppm) -0.1.

Synthesis of compound ***15***

First step: To a 100 mL three-necked flask equipped with a magnetic stir bar, compound **13** (380 mg, 0.53 mmol) and THF (80 mL) were added. The mixture was degassed and charged with nitrogen for three times and stirred at 0 °C (ice-water bath) for 5 min. Then, 2-mesitylmagnesium bromide (1 M solution in THF, 5.3 ml, 5.3 mmol) was added to the mixture at 0 °C. After dripping completely, the mixture was warm to room temperature overnight. H_2O was added to quench the reaction. The residual was extracted by DCM and washed with saturated sodium chloride aqueous solution. The organic layer was dried with anhydrous Na_2SO_4. The crude product of compound **14** was obtained by removal of solvent and used to conduct the next reaction as a yellow solid.

HRMS (MALDI- FTICR) m/z: $[M]^+$ calcd for $C_{68}H_{68}O_4$ 948.5112; found 948.5115; err (ppm) -0.4.

Second step: A 100 mL three-necked flask containing the obtained crude product (**14**) was under vacuum for 15 min, then 40 mL of dry DCM was added, and 0.8 mL of $BF_3 \cdot OEt_2$ was added dropwise through the syringe (turned to dark brown immediately). The mixture was stirred for 30 min at room temperature. Then 5 mL of saturated sodium carbonate (Na_2CO_3) solution was added to quench the reaction, the color of the mixture turned green. The mixture was extracted by DCM, and washed with saturated sodium chloride aqueous solution. The organic layer was dried with anhydrous Na_2SO_4. The solvent was removed under reduced pressure and the residue was directly used for the next reaction (compound **15**).

HRMS (MALDI- FTICR) m/z: $[M]^+$ calcd for $C_{68}H_{64}O_2$ 912.4900; found 912.4899; err (ppm) -0.1.

Synthesis of compound ***2***

To a 100 mL Schlenk flask equipped with a magnetic stir bar, compound **15** (crude product obtained from the above-mentioned second step) and 30 mL dry toluene were added. Then, through a freeze-pump-thaw cycle, the tube was charged with nitrogen for three times again. After the mixture had thawed, 2,3-dichloro-5,6-dicyano-1,4-benzoquinone (DDQ) (430 mg, 1.89 mmol) in 4 mL dry toluene was added to the mixture and the mixture was stirred under N_2 at room temperature for 2 h (the color of the mixture turned to blue-green). After the reaction finished, the mixture was poured into hexane (300 mL) and a large amount of precipitation was generated and filtered off. The precipitation was washed with MeOH. The crude product was further purified by a short silica gel column (DCM/toluene = 4/1, v/v). Compound **2** was obtained as a purple solid (305 mg, 63.3% yield in three steps).

^{1}H NMR (700 MHz, $C_2D_2Cl_4$, 298 K) δ (ppm) 7.75 (s, 2H), 7.62 (s, 2H), 7.39 (d, *J* = 8.5 Hz, 4H), 7.26 (d, *J* = 8.3 Hz, 4H), 6.95 (s, 4H), 6.81 (d, *J* = 7.5 Hz, 2H), 6.59 (d, *J* = 7.7 Hz, 2H), 4.03 (s, 6H), 2.39 (s, 6H), 2.13 (s, 12H), 1.39 (s, 18H).

^{13}C NMR (176 MHz, $C_2D_2Cl_4$, 298 K) δ (ppm) 154.7, 154.6, 152.3, 149.8, 144.2, 143.7, 137.2, 136.4, 134.4, 133.0, 132.7, 132.5, 130.8, 129.6, 128.5, 127.7, 127.3, 125.4, 122.6, 120.4, 116.7, 110.1, 56.3, 34.7, 31.5, 21.5, 20.6.

HRMS (MALDI- FTICR) m/z: $[M]^+$ calcd for $C_{68}H_{62}O_2$ 910.4744; found 910.4743; err (ppm) -0.3.

IR (ATR) 3018 cm^{-1} ($\nu_{C(sp^2)\text{-}H}$), 2985, 2880 cm^{-1} ($\nu_{C(sp^3)\text{-}H}$).

2.3 *Synthesis and characterization of compound **3***

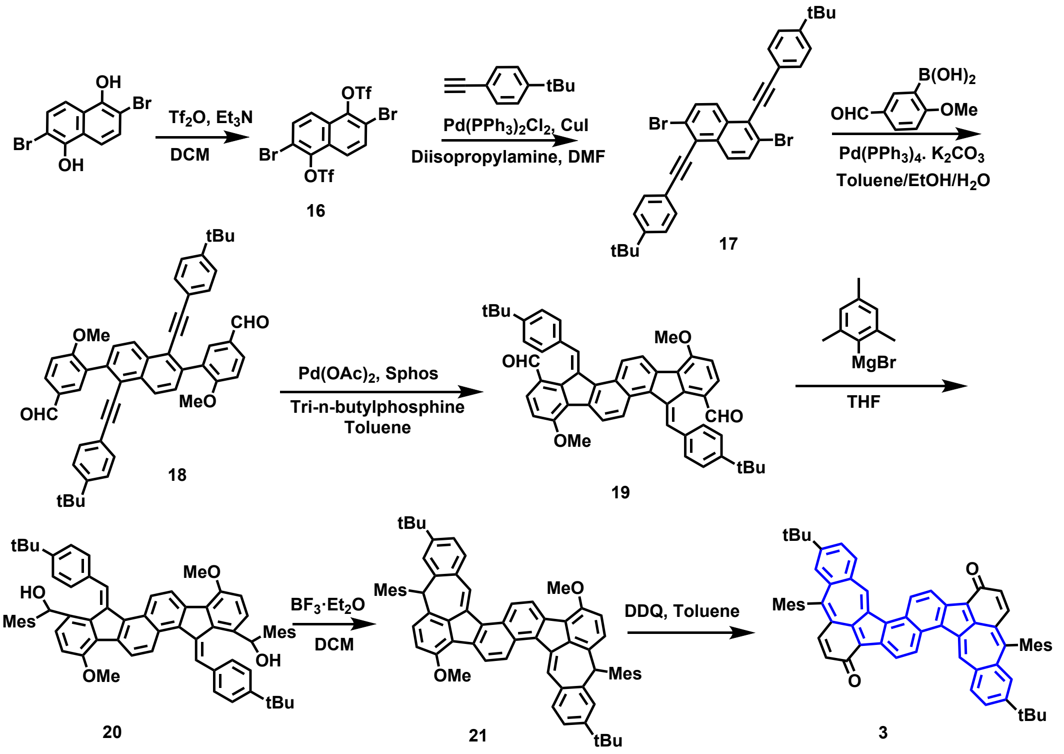

Scheme S3 The synthetic routes of compound **3**

*Synthesis of compound **16***

In a 250 mL three-necked flask equipped with a magnetic stir bar, 2,6-dibromo-1,5-dihydroxynaphthalene (10 g, 31.4 mmol), CH_2Cl_2 (100 mL) and pyridine (10 mL) were added. The mixture was degassed and charged with nitrogen for three times and then cooled to 0 °C (ice-water bath) for 3 min. Then, Trifluoromethanesulfonic anhydride (26.6 g, 94.3 mmol) was added slowly to the mixture under an ice-water bath. After that, it was warmed to room temperature for 12 h. The reaction was quenched with hydrochloric acid solution (1 M, 100 mL), and the mixture was extracted with dichloromethane (100 mL× 3) and washed with water (100 mL× 3). The organic layer was dried with anhydrous Na_2SO_4 and concentrated under reduced pressure. The crude product was then purified by flash column chromatography using petroleum ether (b.p. 60-90 °C) and dichloromethane (10:1, v/v) as eluent to give a white solid **16** (11.904 g, 65.5% yield).

^{1}H NMR (400 MHz, $CDCl_3$, 298 K) δ (ppm): 8.02 (d, J = 9.0 Hz, 2H), 7.88 (d, J = 9.1 Hz, 2H).

^{13}C NMR (101 MHz, $CDCl_3$, 298 K) δ (ppm): 142.5, 133.1, 128.6, 122.9, 122.9, 118.6 (q, 12.8 Hz), 116.6.

*Synthesis of compound **17***

In a 250 mL three-necked flask equipped with a magnetic stir bar, compound **16** (5.2 g, 8.9 mmol), $Pd(PPh_3)_2Cl_2$ (627 mg, 0.9 mmol) and CuI (343 mg, 1.8 mmol). The mixture was degassed and charged with nitrogen for three times and then 1-(tert-butyl)-4-ethynylbenzene (2.8 g, 17.9 mmol), diisopropylamine (20 mL) and DMF (50 mL) were added subsequently. The mixture was stirred at room temperature overnight. The reaction was quenched with hydrochloric acid solution (3 M, 50 mL), and the mixture was extracted with dichloromethane (100 mL×3) and washed with hydrochloric acid solution (3 M, 50 mL×3). The organic layer was dried by anhydrous Na_2SO_4 and concentrated under reduced pressure. The crude product was then purified by flash column chromatography using petroleum ether (b.p. 60-90 °C) as eluent to give a light-yellow solid **17** (4.6 g, 85.3% yield).

^1H NMR (400 MHz, $CDCl_3$, 298 K) δ (ppm) 8.22 (d, J = 8.9 Hz, 2H), 7.75 (d, J = 8.9 Hz, 2H), 7.65-7.60 (m, 4H), 7.47-7.42 (m, 4H), 1.36 (s, 18H).

^{13}C NMR (101 MHz, $CDCl_3$, 298 K) δ (ppm) 152.5, 132.9, 131.6, 131.6, 131.3, 127.6, 125.6, 125.5, 123.2, 119.7, 100.2, 85.4, 34.9, 31.2.

HRMS (MALDI- FTICR) m/z: $[M]^+$ calcd for $C_{34}H_{30}Br_2$ 596.0708; found 596.0716; err (ppm) 1.3.

*Synthesis of compound **18***

In a 250 mL three-necked flask equipped with a magnetic stir bar, compound **17** (3 g, 5 mmol), 2-Methoxy-5-formylphenylboronic acid (2.7 g, 15 mmol), K_2CO_3 (3.5 g, 25 mmol) and $Pd(PPh_3)_4$ (290 mg, 0.25 mmol) were added. The mixture was degassed and charged with nitrogen for three times. Then toluene (120 mL), ethanol (20 mL) and water (20 mL) were added into the flask. The mixture was heated at 110 °C (oil bath) overnight. After cooling to room temperature, the solvent was removed by rotary evaporation. The crude product was then purified by flash column

chromatography using petroleum ether (b.p. 60-90 °C) and dichloromethane (2/1, v/v) as eluent to give a light-yellow solid **18** (1.6 g, 44.1% yield).

^{1}H NMR (400 MHz, $CDCl_3$, 298 K) δ (ppm) 10.00 (s, 2H), 8.57 (d, J = 8.5 Hz, 2H), 8.09 (d, J = 2.2 Hz, 2H), 8.01 (dd, J = 8.5, 2.1 Hz, 2H), 7.64 (d, J = 8.5 Hz, 2H), 7.34-7.31(m, 4H), 7.26-7.22 (m, 4H), 7.18 (d, J = 8.6 Hz, 2H), 3.90 (s, 6H), 1.31 (s, 18H).

^{13}C NMR (101 MHz, $CDCl_3$, 298 K) δ (ppm) 191.0, 162.2, 151.8, 138.8, 134.1, 132.6, 131.5, 131.1, 130.8, 129.5, 128.9, 126.6, 125.4, 120.9, 120.2, 111.1, 98.4, 86.1, 56.1, 34.8, 31.2.

HRMS (MALDI- FTICR) m/z: $[M]^+$ calcd for $C_{50}H_{44}O_4$ 708.3234; found 708.3231; err (ppm) -0.4.

*Synthesis of compound **19***

To a Pyrex tube equipped with a magnetic stir bar, compound **18** (50 mg × 20, 1.41 mmol), $Pd(OAc)_2$ (2.2 mg × 20, 0.19 mmol), 2-dicyclohexylphosphino-2',6'-dimethoxybiphenyl (Sphos, 6.8 mg × 20, 0.32 mmol), Tri-n-butylphosphine (20 μL × 20, 1.6 mmol) and toluene (2 mL × 20) were added sequentially. Then, through a freeze-pump-thaw cycle, the tube was charged with nitrogen for three times again. After raising to room temperature, the mixture was heated at 120 °C for 12 h. After cooling to room temperature, a large amount of precipitation was generated and filtered off. The precipitation was washed with MeOH. The residue was collected and dried under vacuum to afford compound **19** (967 mg, 96.7%), which was directly used for the next step without further purification.

^{1}H NMR (400 MHz, $CDCl_3$, 298 K) δ (ppm) 9.74 (s, 2H), 8.63 (d, J = 8.8 Hz, 2H), 8.47 (d, J = 8.7 Hz, 2H), 8.23 (s, 2H), 7.70 (d, J = 8.6 Hz, 2H), 7.37 (s, 8H), 7.02 (d, J = 8.6 Hz, 2H), 4.15 (s, 6H), 1.33 (s, 18H).

^{13}C NMR (101 MHz, $CDCl_3$, 298 K) δ (ppm) 188.3, 159.2, 152.1, 142.2, 138.5, 138.2, 137.3, 133.9, 129.4, 128.7, 127.7, 127.3, 126.3, 125.1, 124.8, 123.2, 110.9, 55.8, 34.8, 31.2.

HRMS (MALDI- FTICR) m/z: $[M]^+$ calcd for $C_{50}H_{44}O_4$ 708.3234; found 708.3232; err (ppm) -0.2.

*Synthesis of compound **3***

First step: To a 50 mL three-necked flask equipped with a magnetic stir bar, compound 9 (200 mg, 0.28 mmol) and THF (25 mL)were added. The mixture was degassed and charged with nitrogen for three times and stirred at 0 °C (ice-water bath) for 5 min. Then, 2-Mesitylmagnesium bromide (1 M solution in THF, 4.5 mL, 4.5 mmol) was added to the mixture at 0 °C. After dripping completely, the mixture was warm to room temperature overnight. Then, H_2O was added to quench the reaction. The residual was extracted by DCM, and washed with saturated sodium chloride aqueous solution. The organic layer was dried with anhydrous Na_2SO_4. The crude product of compound **20** was obtained by removal of solvent and used to conduct the next reaction as a yellow solid.

HRMS (MALDI- FTICR) m/z: $[M]^+$ calcd for $C_{68}H_{68}O_4$ 948.5112; found 948.5118; err (ppm) 0.7.

Second step: A 100 mL three-necked flask containing the obtained crude product was under vacuum for 15 min, then 25 mL of dry DCM was added, 0.4 mL of $BF_3 \cdot OEt_2$ was added dropwise through syringe (turned to dark brown immediately) under 0 °C (ice-water bath). Then, the mixture was kept stirred for 4 h at 50 °C. Then 2 mL of saturated sodium carbonate (Na_2CO_3) solution was added to quench the reaction. The mixture was extracted by DCM, and washed with saturated sodium chloride aqueous solution. The organic layer was dried with anhydrous Na_2SO_4. The solvent was removed under reduced pressure and the residue was directly used for the next reaction (compound **21**).

HRMS (MALDI- FTICR) m/z: $[M]^+$ calcd for $C_{68}H_{64}O_2$ 912.4900; found 912.4899; err (ppm) -0.2.

Third step: To a 50 mL Schlenk flask equipped with a magnetic stir bar, compound **21** (crude product obtained from the second step) and 15 mL dry toluene were added.

Then, through a freeze-pump-thaw cycle, the tube was charged with nitrogen for three times. After the mixture had thawed, 2,3-dichloro-5,6-dicyano-1,4-benzoquinone (DDQ) (71.8 mg, 0.32 mmol) in 2 mL dry toluene was added to the mixture and the mixture was stirred under N_2 at room temperature for 0.5 h. Then, H_2O (5 mL) and Et_3N (2 mL) were added to quench the reaction. The residual was extracted by DCM, and washed with saturated sodium chloride aqueous solution. The organic layer was dried with anhydrous Na_2SO_4 and the solvent was removed by rotary evaporation. The crude product was then purified by flash column chromatography using dichloromethane and methanol (1/0~20/1, v/v) as eluent to give a brown solid of **3** (19 mg, 19.7% yield).

^{1}H NMR (700 MHz, $CDCl_3$, 298 K) δ (ppm) 9.85 (s, 2H), 9.32 (d, J = 8.6 Hz, 2H), 9.23 (d, J = 8.5 Hz, 2H), 8.46 (d, J = 8.2 Hz, 2H), 8.02 (dd, J = 8.1, 1.9 Hz, 2H), 7.89 (d, J = 1.9 Hz, 2H), 7.54 (d, J = 10.0 Hz, 2H), 7.14 (s, 4H), 6.93 (d, J = 10.0 Hz, 2H), 2.50 (s, 6H), 1.88 (s, 12H), 1.25 (s, 18H).

^{13}C NMR (176 MHz, $CDCl_3$, 298 K) δ (ppm) 154.4, 141.6, 140.3, 139.2, 138.2, 138.0, 137.9, 137.6, 137.4, 136.1, 135.9, 134.2, 133.4, 130.3, 130.2, 129.1, 129.0, 128.9, 128.6, 126.7, 126.0, 123.7, 35.3, 30.9, 21.3, 20.1.

HRMS (MALDI- FTICR) m/z: $[M]^+$ calcd for $C_{66}H_{56}O_2$ 880.4274; found 880.4269; err (ppm) 0.6.

IR (ATR) 3021 cm^{-1} ($\nu_{C(sp^2)\text{-}H}$), 2927, 2853 cm^{-1} ($\nu_{C(sp^3)\text{-}H}$), 1613 cm^{-1} ($\nu_{C=O}$).

*2.4 The proposed mechanism of demethylation for the synthesis of **3** after treating **21** with DDQ*[11]

As shown in Scheme S4, first, the electron-rich methoxy-substituted substrate (**21**) donates an electron to the electrophilic quinone oxidant (DDQ), forming a charge-transfer complex (**3-1**). This is followed by the abstraction of a benzylic proton (highlighted in red) by the carbonyl oxygen of DDQ and forming the oxonium ion intermediate (**3-2**). Subsequent hydrolysis, mediated by water, cleaves the C-O bond, converting the quinone into a phenol derivative and forming **3-3**. Then, the single carbonyl-substituted compound of **3-4** was yielded by releasing the methoxy group as methanol (MeOH). After the second oxidative cyclization event occurs, we can obtain the final product of **3**.

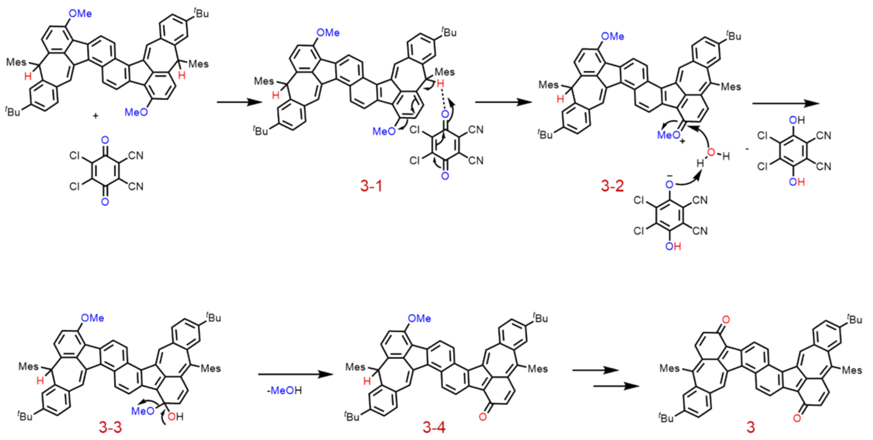

Scheme S4 The proposed mechanism of demethylation for the synthesis of **3** after treating **21** with DDQ.

3. The ^1H-^1H NOESY of compounds

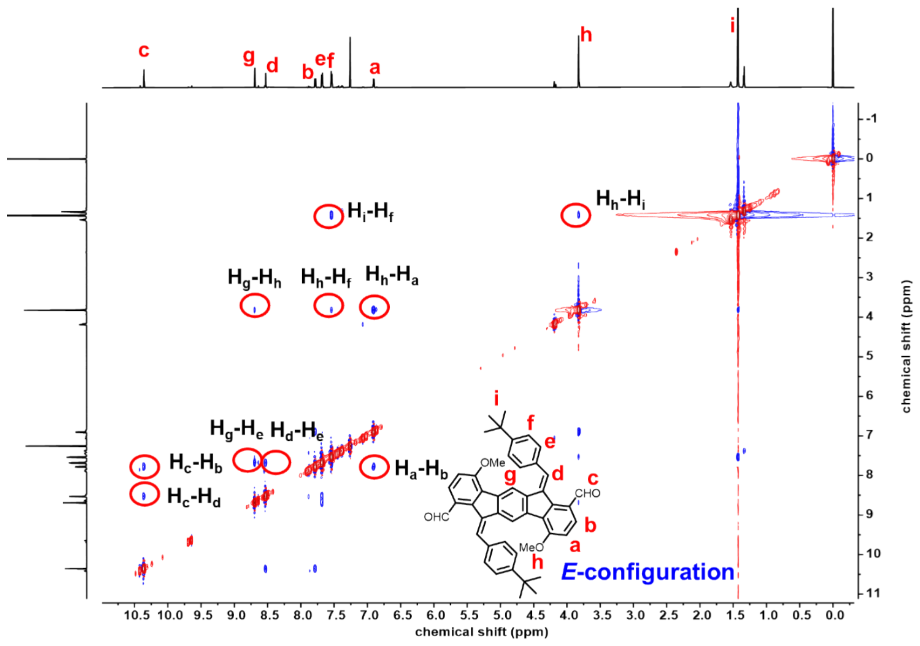

Figure S1 The NOESY of compound **7** (700 MHz, $C_2D_2Cl_4$, 298 K).

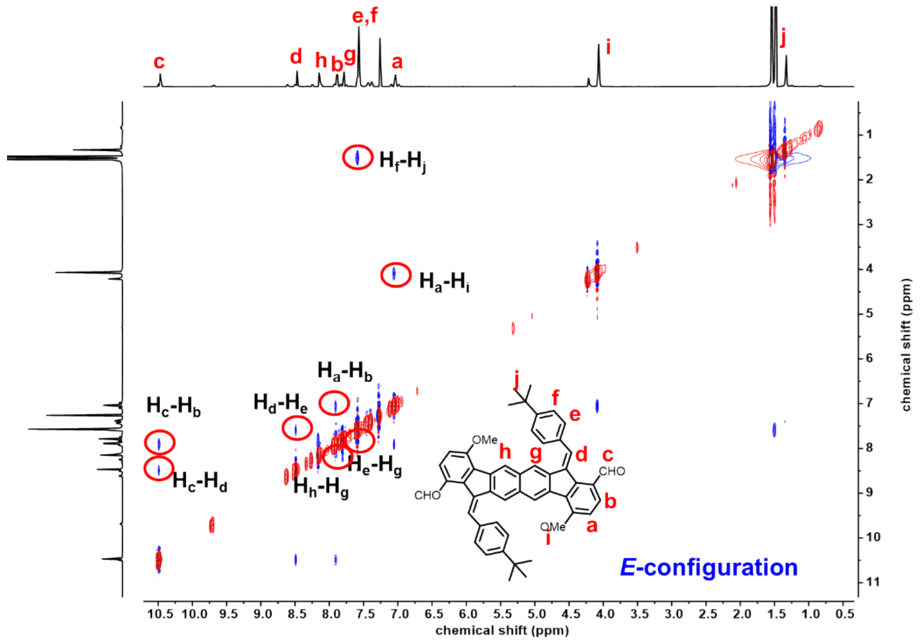

Figure S2 The NOESY of compound **13** (700 MHz, $C_2D_2Cl_4$, 298 K).

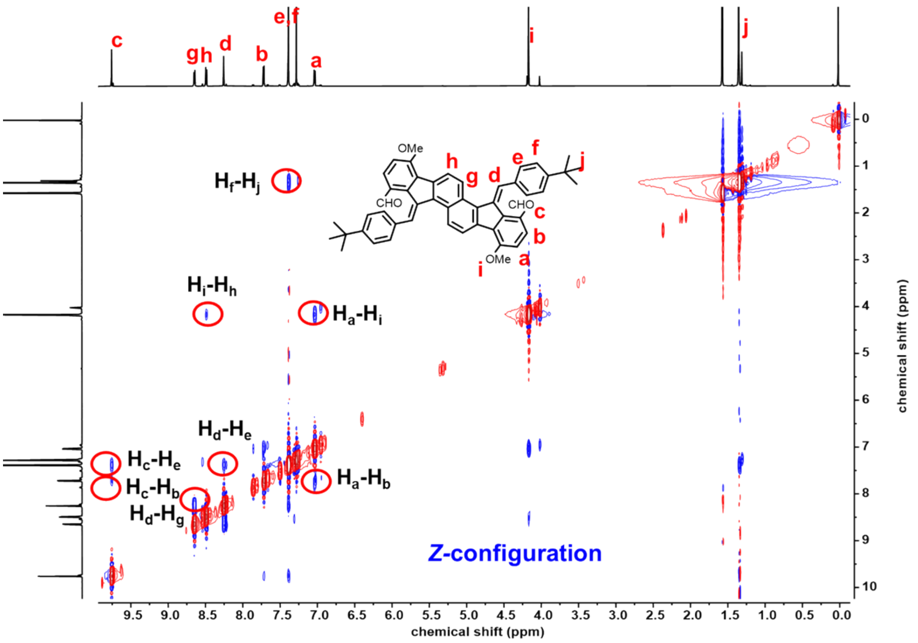

Figure S3 The NOESY of compound **19** (700 MHz, $C_2D_2Cl_4$, 298 K).

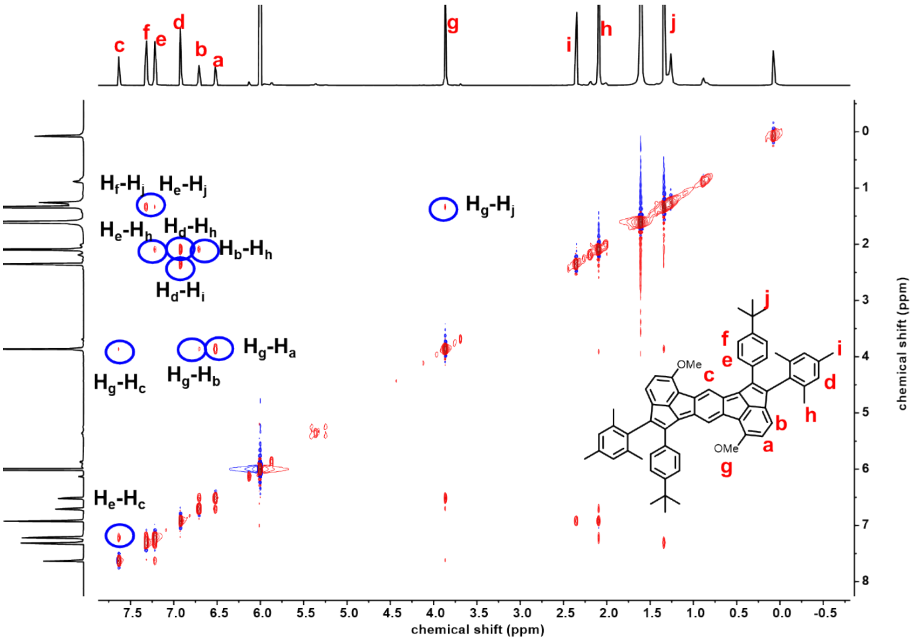

Figure S4 The NOESY of compound **1** (700 MHz, $C_2D_2Cl_4$, 298 K).

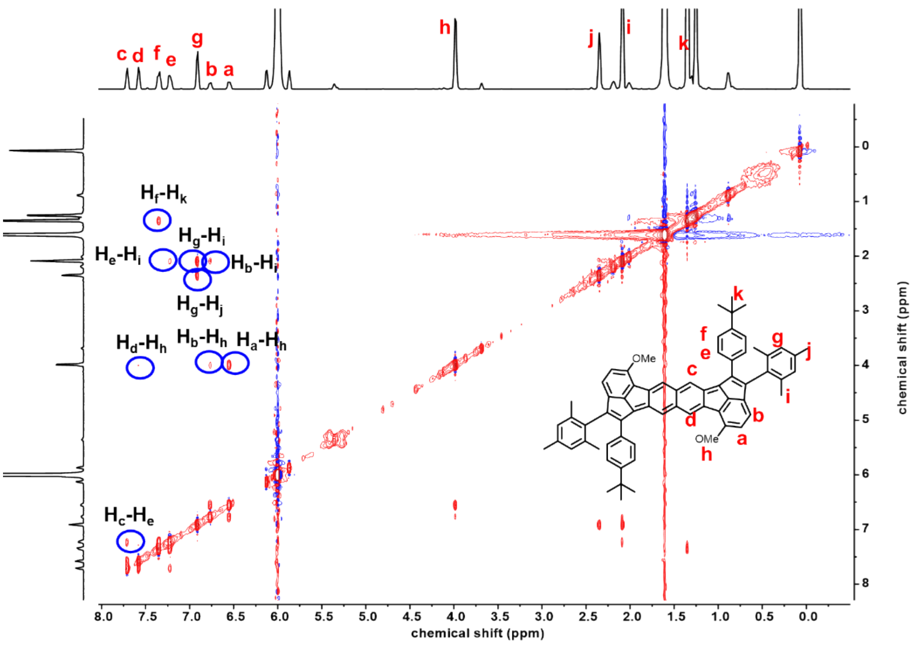

Figure S5 The NOESY of compound **2** (700 MHz, $C_2D_2Cl_4$, 298 K).

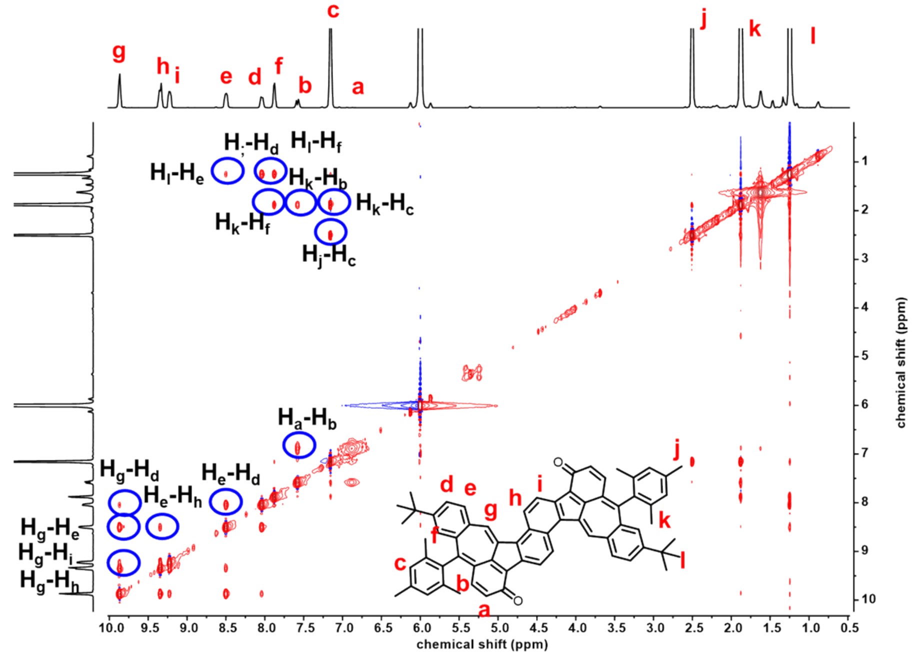

Figure S6 The NOESY of compound **3** (700 MHz, $C_2D_2Cl_4$, 298 K).

4. Crystallographic data

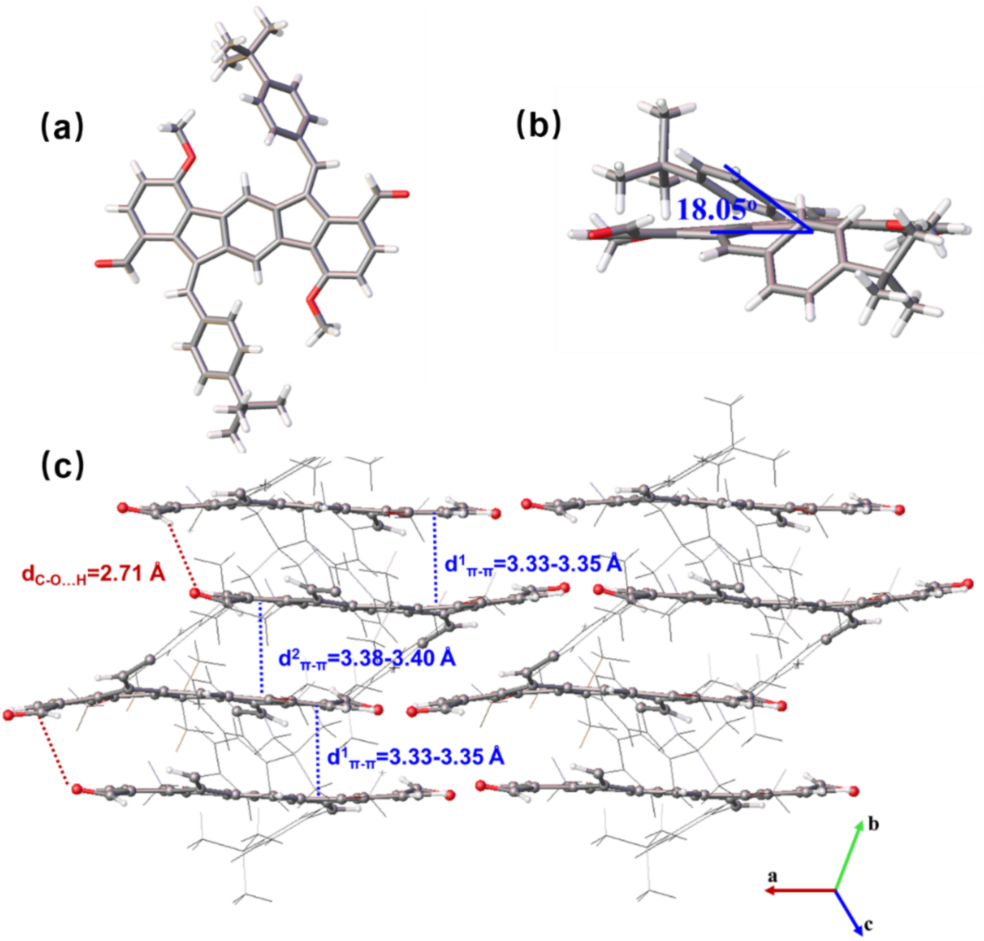

Figure S7 X-ray single-crystal crystallographic analysis. Structure of (a) top view, (b) side view and (c) intermolecular packing mode and interaction of intermediate **7**. Displacement ellipsoids are drawn at 50% probability level. The solvent molecule of $CHCl_3$ was omitted for clarity.

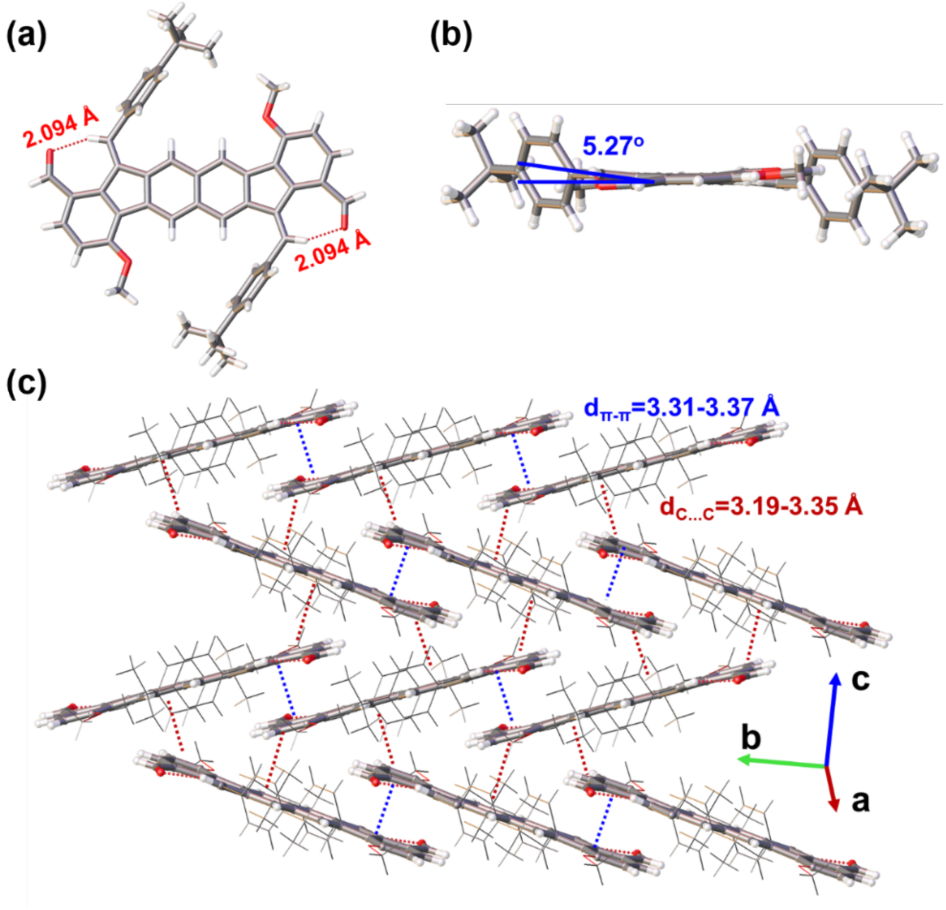

Figure S8 X-ray single-crystal crystallographic analysis. Structure of (a) top view, (b) side view and (c) intermolecular packing mode and interaction of intermediate **13**. Displacement ellipsoids are drawn at 50% probability level.

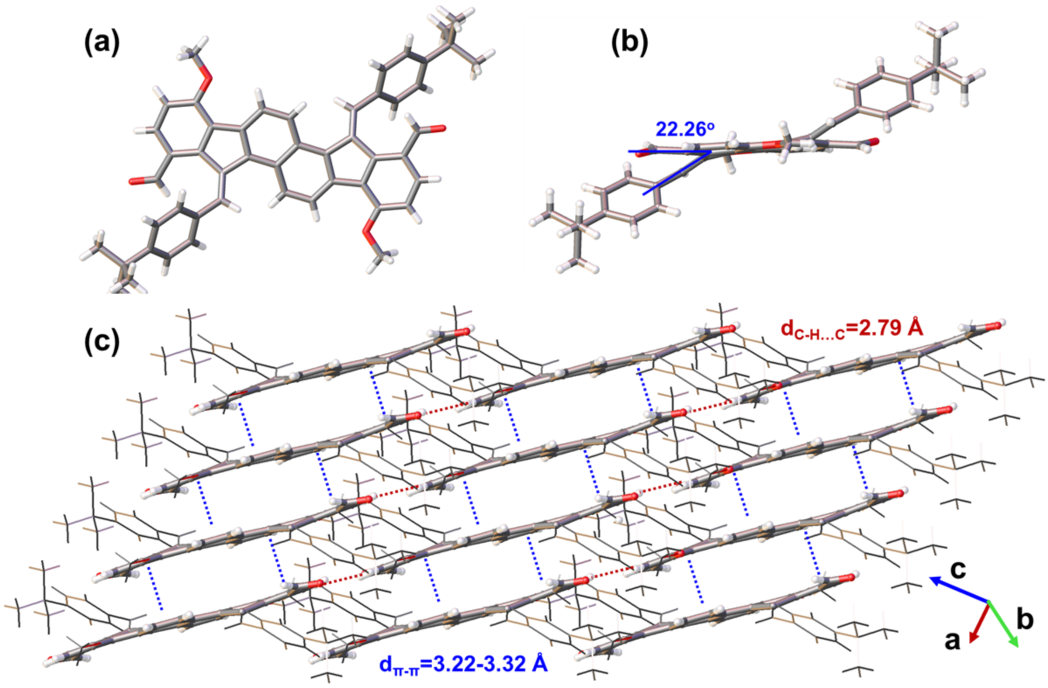

Figure S9 X-ray single-crystal crystallographic analysis. Structure of (a) top view, (b) side view and (c) intermolecular packing mode and interaction of intermediate **19**. Displacement ellipsoids are drawn at 50% probability level.

Table S1 Crystallographic data of compound **7**.

Compound	**7**
CCDC deposition NO.	2478865
Empirical formula	$C_{94}H_{86}Cl_6O_8$
Formula weight	1556.32
Temperature/K	170 (1)
Crystal system	triclinic
Space group	P-1
a/Å	14.54950(10)
b/Å	15.2378(2)
c/Å	19.4658(2)
α/°	97.6160(10)
β/°	100.2140(10)

γ/°	108.7880(10)
Volume/Å^3	3936.74(8)
Z	2
ρ_{calc}g/cm^3	1.313
μ/mm^{-1}	2.458
F(000)	1632.0
Crystal size/mm^3	0.33× 0.23 × 0.15
Radiation	CuKα (λ = 1.54184)
2Θ range for data collection/°	4.712 to 151.864
Index ranges	-18 ≤ h ≤ 16, -18≤ k ≤ 19, -24 ≤ l ≤ 23
Reflections collected	56938
Independent reflections	15942 [R_{int} = 0.0285, R_{sigma} = 0.0291]
Data/restraints/parameters	15942/0/989
Goodness-of-fit on F^2	1.071
Final R indexes [I>=2σ (I)]	R_1 = 0.0649, wR_2 = 0.1821
Final R indexes [all data]	R_1 = 0.0739, wR_2 = 0.1906
Largest diff. peak/hole / e Å^{-3}	1.50/-1.19

Table S2 Crystallographic data of compound **13**.

Compound	**13**
CCDC deposition NO.	2478866
Empirical formula	$C_{25}H_{22}O_2$
Formula weight	354.42
Temperature/K	170.00 (10)
Crystal system	monoclinic
Space group	P2$_1$/c
a/Å	16.3766(3)
b/Å	10.38959(18)
c/Å	11.4772(3)
α/°	90
β/°	103.825(10)

γ/°	90
Volume/Å^3	1896.22(7)
Z	4
ρ_{calc}g/cm^3	1.241
μ/mm^{-1}	0.605
F(000)	752.0
Crystal size/mm^3	0.5× 0.3 × 0.1
Radiation	CuKα (λ = 1.54184)
2Θ range for data collection/°	5.558 to 155.072
Index ranges	-20 ≤ h ≤ 19, -11≤ k ≤ 13, -14 ≤ l ≤ 14
Reflections collected	14107
Independent reflections	3878 [R_{int} = 0.0264, R_{sigma} = 0.0257]
Data/restraints/parameters	3878/0/248
Goodness-of-fit on F^2	1.066
Final R indexes [I>=2σ (I)]	R_1 = 0.0592, wR_2 = 0.1581
Final R indexes [all data]	R_1 = 0.0667, wR_2 = 0.1637
Largest diff. peak/hole / e Å^{-3}	0.71/-0.40

Table S3 Crystallographic data of compound **19**.

Compound	**19**
CCDC deposition NO.	2478867
Empirical formula	$C_{25}H_{22}O_2$
Formula weight	354.42
Temperature/K	170.00 (3)
Crystal system	triclinic
Space group	P-1
a/Å	5.6358(3)
b/Å	12.7956(6)
c/Å	12.9671(5)
α/°	97.392(4)
β/°	93.846(4)

γ/°	97.333(4)
Volume/Å^3	916.43(8)
Z	2
ρ_{calc}g/cm^3	1.284
μ/mm^{-1}	0.626
F(000)	376.0
Crystal size/mm^3	0.25× 0.15 × 0.12
Radiation	CuKα (λ = 1.54184)
2Θ range for data collection/°	6.898 to 151.448
Index ranges	-7 ≤ h ≤ 7, -15≤ k ≤ 15, -16 ≤ l ≤ 12
Reflections collected	10280
Independent reflections	3649 [R_{int} = 0.0338, R_{sigma} = 0.0420]
Data/restraints/parameters	3649/0/248
Goodness-of-fit on F^2	1.089
Final R indexes [I>=2σ (I)]	R_1 = 0.0762, wR_2 = 0.2130
Final R indexes [all data]	R_1 = 0.0892, wR_2 = 0.2262
Largest diff. peak/hole / e Å^{-3}	0.42/-0.23

Table S4 Crystallographic data of compound **1**.

Compound	**1**
CCDC deposition NO.	2478868
Empirical formula	$C_{32}H_{30}O$
Formula weight	430.56
Temperature/K	170.00 (10)
Crystal system	triclinic
Space group	P-1
a/Å	9.8909(4)
b/Å	10.4826(5)
c/Å	13.2687(3)
α/°	108.996(3)
β/°	97.316(3)

$\gamma/°$	109.909(4)
Volume/$Å^3$	1178.35(9)
Z	2
$\rho_{calc}g/cm^3$	1.213
μ/mm^{-1}	0.543
F(000)	460.0
Crystal size/mm^3	0.1× 0.08 × 0.03
Radiation	CuKα (λ = 1.54184)
2Θ range for data collection/°	7.314 to 154.992
Index ranges	$-12 \leq h \leq 12$, $-12\leq k \leq 13$, $-16 \leq l \leq 16$
Reflections collected	14522
Independent reflections	4796 [R_{int} = 0.0460, R_{sigma} = 0.0462]
Data/restraints/parameters	4796/0/305
Goodness-of-fit on F^2	1.052
Final R indexes [I>=2σ (I)]	R_1 = 0.0700, wR_2 = 0.1989
Final R indexes [all data]	R_1 = 0.0870, wR_2 = 0.2173
Largest diff. peak/hole / e $Å^{-3}$	0.44/-0.24

Table S5 Crystallographic data of compound **2**.

Compound	**2**
CCDC deposition NO.	2478869
Empirical formula	$C_{72}H_{66}Cl_{12}O_2$
Formula weight	1388.64
Temperature/K	100.00 (10)
Crystal system	monoclinic
Space group	$P2_1/n$
a/Å	15.5670(4)
b/Å	13.0056(2)
c/Å	18.0182(4)
α/°	90
β/°	113.360(3)
γ/°	90
Volume/Å^3	3348.92(14)
Z	2
ρ_{calc}g/cm^3	1.377
μ/mm^{-1}	4.897
F(000)	1436.0
Crystal size/mm^3	0.2× 0.12 × 0.08
Radiation	CuKα (λ = 1.54184)
2Θ range for data collection/°	6.372 to 155.974
Index ranges	$-19 \leq h \leq 19$, $-9 \leq k \leq 16$, $-22 \leq l \leq 22$
Reflections collected	23526
Independent reflections	6916 [R_{int} = 0.0440, R_{sigma} = 0.0413]
Data/restraints/parameters	6916/0/395
Goodness-of-fit on F^2	1.063
Final R indexes [I>=2σ (I)]	R_1 = 0.0592, wR_2 = 0.1578
Final R indexes [all data]	R_1 = 0.0708, wR_2 = 0.1652
Largest diff. peak/hole / e Å^{-3}	1.36/-0.85

Table S6 Crystallographic data of compound **3**.

Compound	**3**
CCDC deposition NO.	2478870
Empirical formula	$C_{198}H_{168}O_6$
Formula weight	2643.31
Temperature/K	169.97 (12)
Crystal system	triclinic
Space group	P-1
a/Å	18.9545(5)
b/Å	20.8030(7)
c/Å	21.5827(6)
α/°	88.929(2)
β/°	65.138(3)
γ/°	80.456(3)
Volume/Å^3	7602.3(4)
Z	2
ρ_{calc}g/cm^3	1.155
μ/mm^{-1}	0.519
F(000)	2808.0
Crystal size/mm^3	0.2× 0.14 × 0.005
Radiation	CuKα (λ = 1.54184)
2Θ range for data collection/°	4.314 to 151.922
Index ranges	$-23 \le h \le 23$, $-25 \le k \le 26$, $-26 \le l \le 26$
Reflections collected	98493
Independent reflections	30211 [R_{int} = 0.1595, R_{sigma} = 0.1264]
Data/restraints/parameters	30211/0/1874
Goodness-of-fit on F^2	0.963
Final R indexes [I>=2σ (I)]	R_1 = 0.0746, wR_2 = 0.1791
Final R indexes [all data]	R_1 = 0.1599, wR_2 = 0.2221
Largest diff. peak/hole / e Å^{-3}	0.40/-0.24

5. Thermogravimetric analysis (TGA) analysis

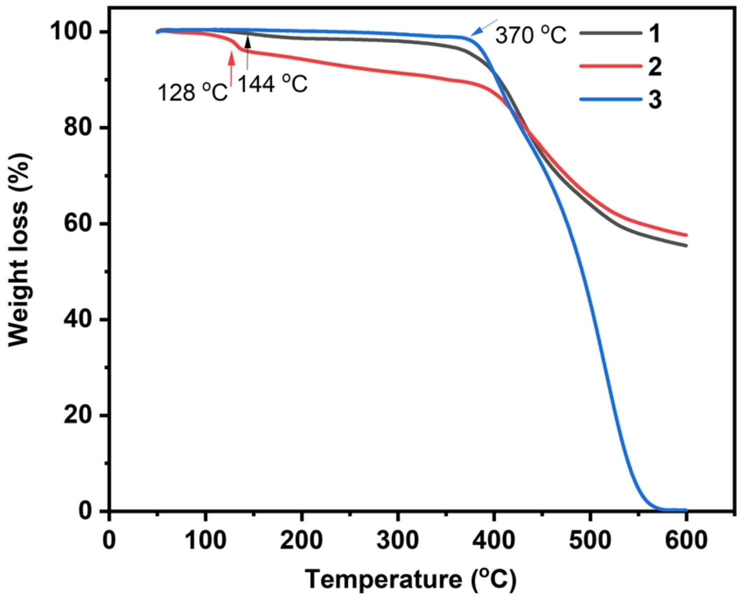

Figure S10 TGA curves of **1**, **2** and **3**; heating rate: 10 °C/min from 50 °C to 600 °C under nitrogen atmosphere.

6. Infrared spectra analysis

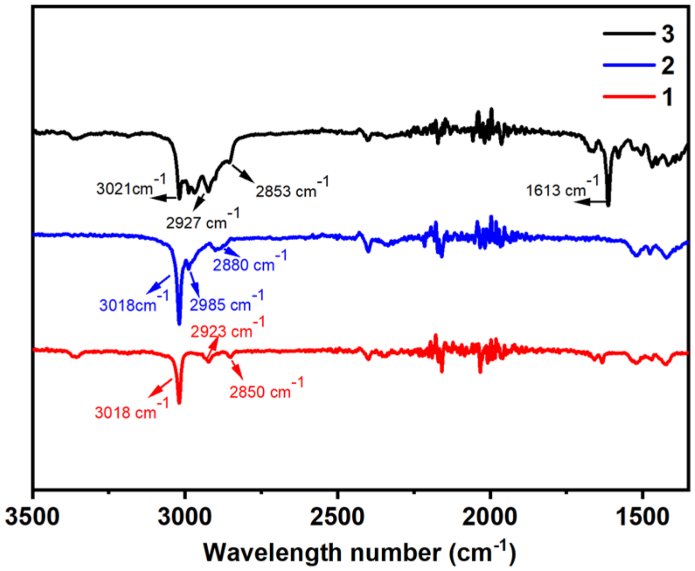

Figure S11 The IR spectra of compounds **1-3**

7. Calculations on electronic properties

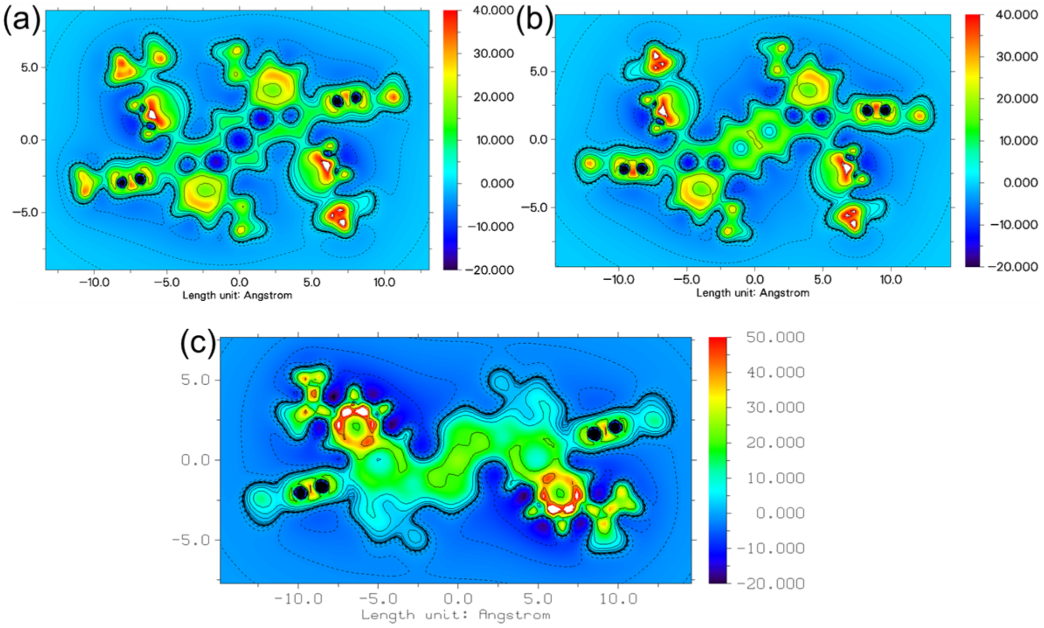

Figure S12 Isotropic Chemical Shielding Surface at 1 Å of Z axis (ICSS(1)$_{zz}$) for (a) **1**, (b) **2** and (c) **3**.

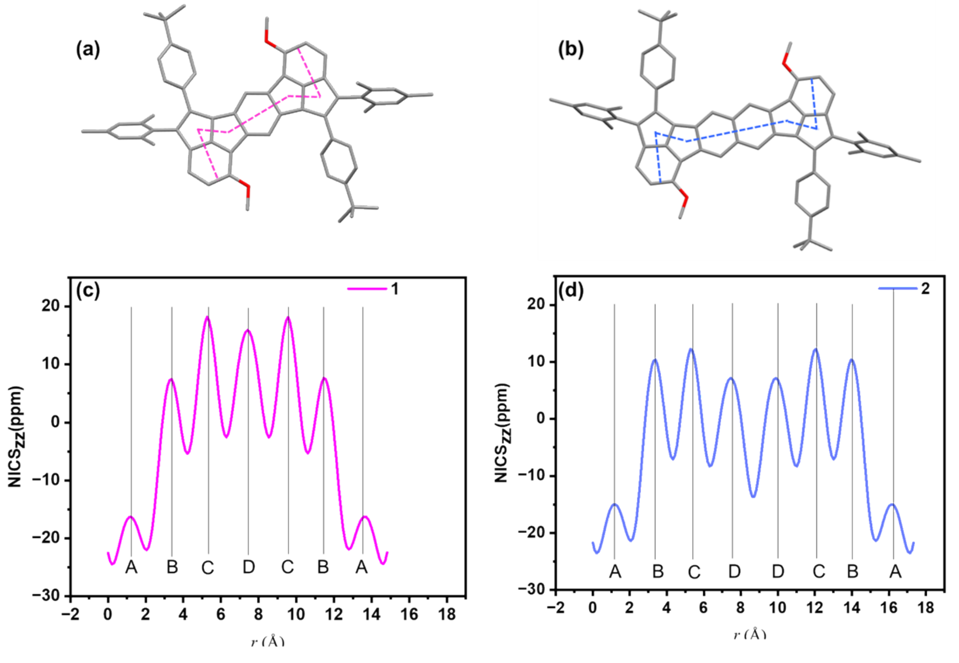

Figure S13 The NICS-XY scan lines of two antiaromatic compounds of (a) **1** and (b) **2**. The corresponding NICS values of (c) **1** and (d) **2** along the scan lines

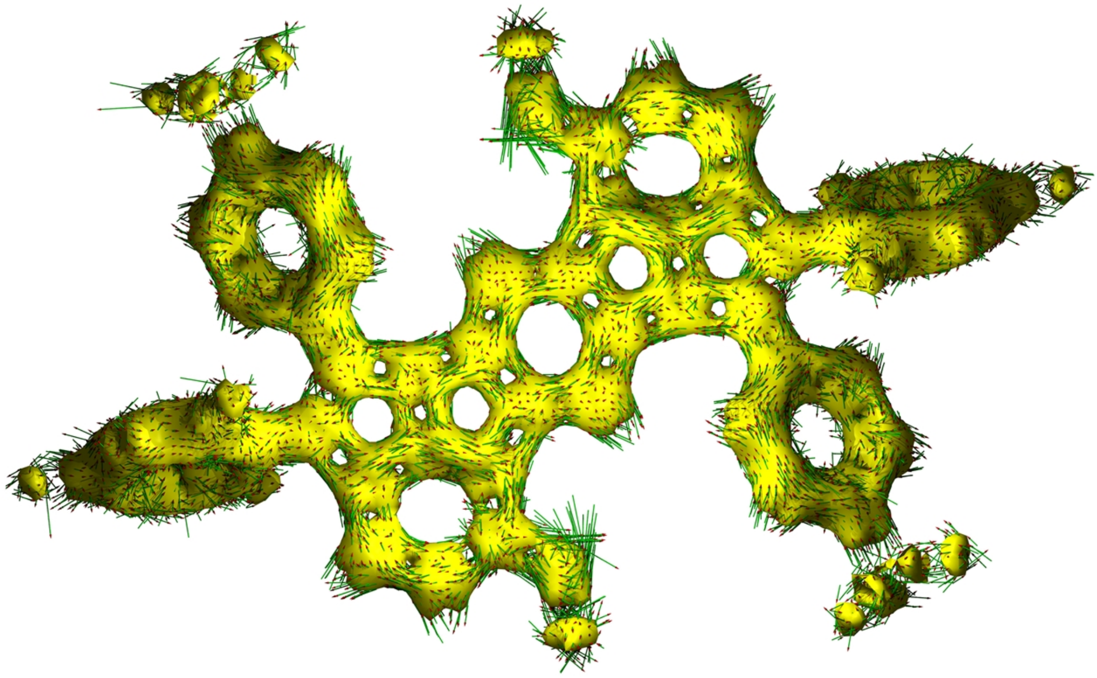

Figure S14 The calculated ACID plots of compound **1** (closed-shell state)

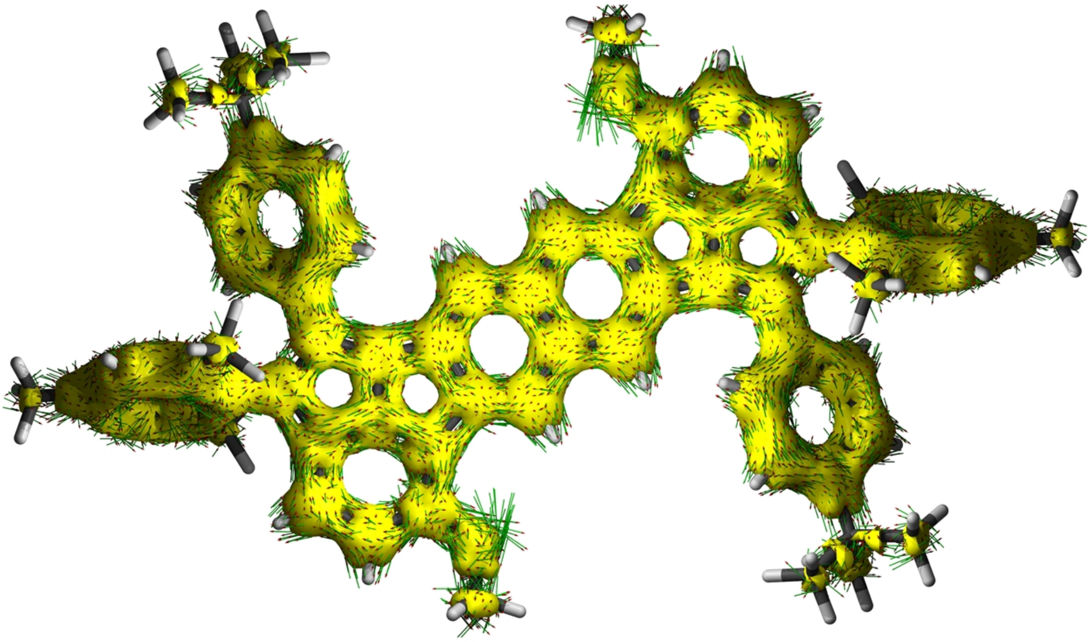

Figure S15 The calculated ACID plots of compound **2** (open-shell state)

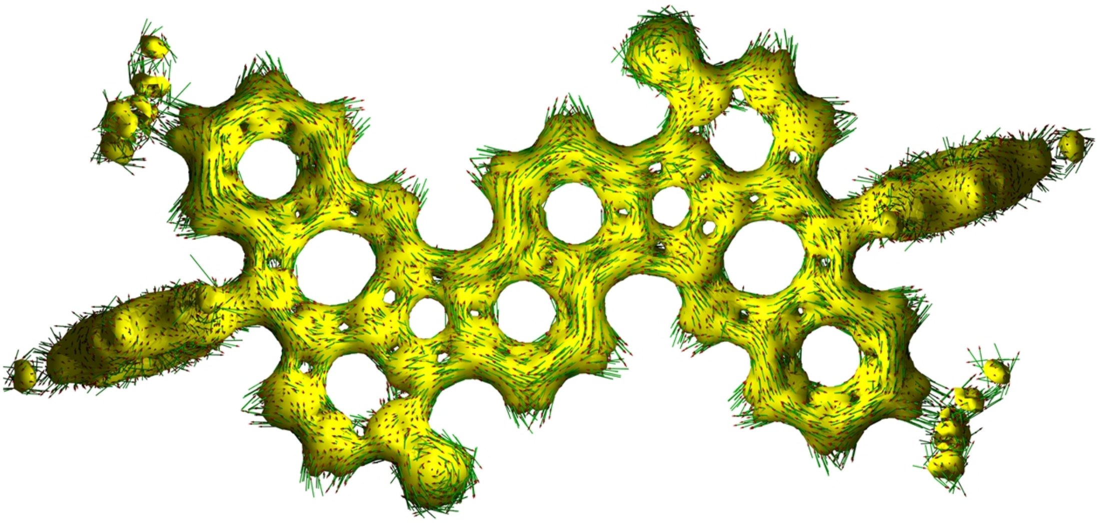

Figure S16 The calculated ACID plots of compound **3** (closed-shell state)

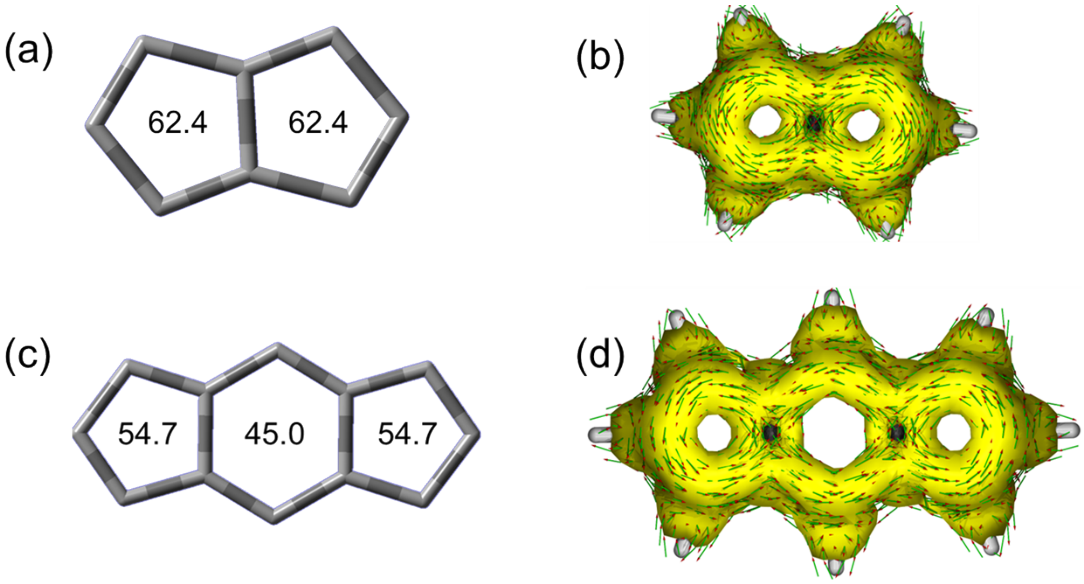

Figure 17 The calculated NICS (a, c) and ACID plots (b, d) of pristine pentalene (a, b) and s-indacene (c, d) units.

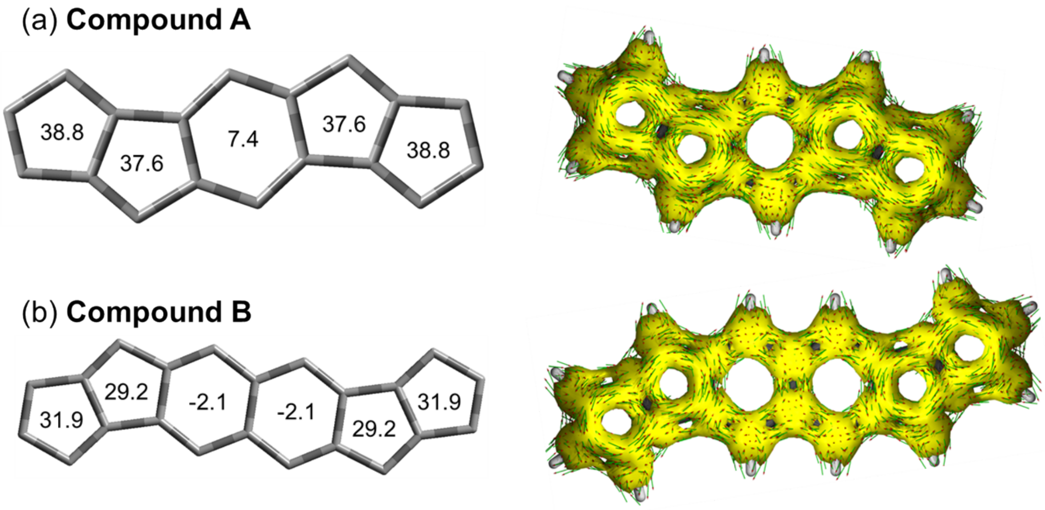

Figure S18 The model compounds of (a) bis(pentalene)-fused benzene or (b) naphthalene derivatives.

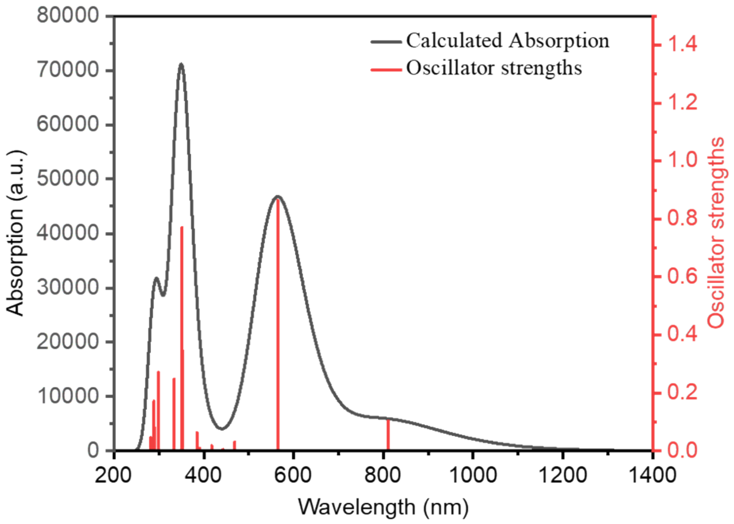

Figure S19 Calculated UV-vis absorption spectra of the **1** (closed-shell single state) based on TD-B3LYP-D3(BJ)/6-31G(d,p) level of theory

Table S7 Excitation energies, oscillator strengths and orbital contribution of the $S_0 \rightarrow S_n$ excitations of **1** calculated at the TD-B3LYP-D3(BJ)/6-31G(d,p) level of theory.

transition	energy [eV]	energy [nm]	oscillator strength	orbital contribution	
$S_0 \rightarrow S_1$	1.4772	839.32	0.00010	H-1 → L	95.9%
$S_0 \rightarrow S_2$	1.5298	810.46	0.10370	H → L	74.2%
				H-3 → L	25.3%
$S_0 \rightarrow S_3$	1.6801	737.96	0.00000	H-2 → L	96.0%
$S_0 \rightarrow S_4$	2.1946	564.95	0.86380	H-3 → L	70.8%
				H → L	23.4%
$S_0 \rightarrow S_5$	2.6274	471.89	0.00010	H-4 → L	97.9%
$S_0 \rightarrow S_6$	2.6429	469.12	0.03100	H-5 → L	96.1%
$S_0 \rightarrow S_7$	2.8008	442.67	0.00510	H-6 → L	98.0%
$S_0 \rightarrow S_8$	2.8057	441.90	0.00150	H-7 → L	98.4%

$S_0 \rightarrow S_9$	2.9678	417.76	0.01880	H-8 → L	92.5%
$S_0 \rightarrow S_{10}$	2.9818	415.80	0.00260	H-9 → L	87.6%
				H → L+1	7.1%

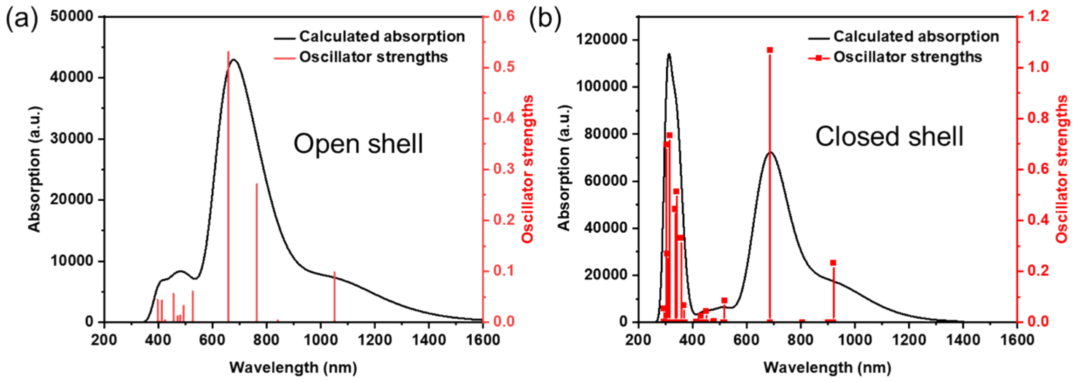

Figure S20 Calculated UV-vis absorption spectra of the compound **2** in (a) open-shell and (b) closed-shell states based on TD-(U)B3LYP-D3(BJ)/6-31G(d,p) level of theory.

Table S8 Excitation energies, oscillator strengths and orbital contribution of the $S_0 \rightarrow S_n$ excitations of **2** calculated at the TD-UB3LYP-D3(BJ)/6-31G(d,p) level of theory.

transition	energy [eV]	energy [nm]	oscillator strength	orbital contribution	
$S_0 \rightarrow S_1$	1.9697	629.46	0.01680	H → L	71.4%
				H→ L+2	20.9%
$S_0 \rightarrow S_2$	2.1561	575.04	0.00480	H → L+1	79.6%
				H → L+3	8.5%
$S_0 \rightarrow S_3$	2.1946	564.95	0.15430	H → L+2	68.8%
				H → L	20.8%
$S_0 \rightarrow S_4$	2.4113	514.18	0.00010	H → L+3	80.8%
				H → L+1	8.9%
$S_0 \rightarrow S_5$	2.5209	491.83	0.45790	H-1 → L	94.6%
$S_0 \rightarrow S_6$	2.6278	471.82	0.00630	H-1 → L+1	79.0%
$S_0 \rightarrow S_7$	2.6573	466.58	0.01670	H-3 → L	47.0%
				H-4 →L+1	22.9%
				H-3 → L+2	10.3%
				H-3 → L+1	6.9%
$S_0 \rightarrow S_8$	2.6674	464.81	0.00030	H-4 → L	33.5%
				H-3 → L+1	26.2%
				H-4 → L+1	22.7%
				H-1 → L+1	9.2%
$S_0 \rightarrow S_9$	2.7240	455.15	0.43700	H-1 → L+2	84.1%
				H-2 → L+1	5.1%
$S_0 \rightarrow S_{10}$	2.8298	438.14	0.00400	H-2 → L	75.4%
				H-2 → L+2	10.6%

Table S9 Excitation energies, oscillator strengths and orbital contribution of the $S_0 \rightarrow S_n$ excitations of **2** calculated at the TD-B3LYP-D3(BJ)/6-31G(d,p) level of theory.

transition	energy [eV]	energy [nm]	oscillator strength	orbital contribution	
$S_0 \rightarrow S_1$	1.3470	920.45	0.23230	H → L<br>H-2 → L	52.6%<br>48.1%
$S_0 \rightarrow S_2$	1.3761	900.98	0.00000	H-1 → L	99.0%
$S_0 \rightarrow S_3$	1.5398	805.20	0.00000	H-3 → L	98.9%
$S_0 \rightarrow S_4$	1.8073	686.02	1.06690	H-2 → L<br>H → L	50.4%<br>48.4%
$S_0 \rightarrow S_5$	2.3960	517.46	0.08580	H-5 → L	98.2%
$S_0 \rightarrow S_6$	2.4191	512.52	0.00000	H-4 → L	96.9%
$S_0 \rightarrow S_7$	2.5909	478.54	0.00360	H-6 → L	99.2%
$S_0 \rightarrow S_8$	2.5965	477.51	0.00110	H-7 → L	99.3%
$S_0 \rightarrow S_9$	2.7511	450.67	0.04360	H-8 → L	94.9%
$S_0 \rightarrow S_{10}$	2.7719	447.29	0.00000	H-9 → L<br>H → L+1	86.4%<br>9.1%

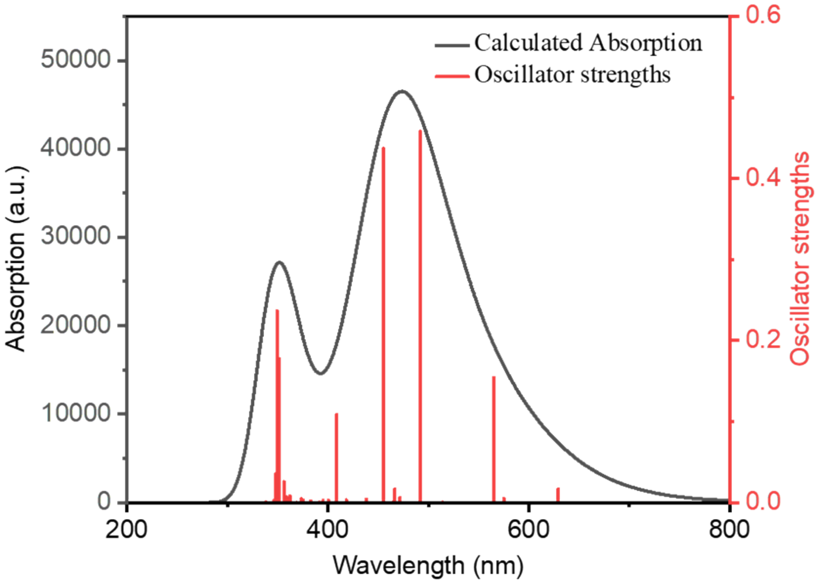

Figure S21 Calculated UV-vis absorption spectra of the **3** (closed-shell single state) based on TD-B3LYP-D3(BJ)/6-31G(d,p) level of theory

Table S10 Excitation energies, oscillator strengths and orbital contribution of the $S_0 \rightarrow S_n$ excitations of **3** calculated at the TD-B3LYP-D3(BJ)/6-31G(d,p) level of theory.

transition	energy [eV]	energy [nm]	oscillator strength	orbital contribution	
$S_0 \rightarrow S_1$	1.9697	629.46	0.01680	H → L	71.4%
				H→ L+2	20.9%
$S_0 \rightarrow S_2$	2.1561	575.04	0.00480	H → L+1	79.6%
				H → L+3	8.5%
$S_0 \rightarrow S_3$	2.1946	564.95	0.15430	H → L+2	68.8%
				H → L	20.8%
$S_0 \rightarrow S_4$	2.4113	514.18	0.00010	H → L+3	80.8%
				H → L+1	8.9%
$S_0 \rightarrow S_5$	2.5209	491.83	0.45790	H-1 → L	94.6%
$S_0 \rightarrow S_6$	2.6278	471.82	0.00630	H-1 → L+1	79.0%
$S_0 \rightarrow S_7$	2.6573	466.58	0.01670	H-3 → L	47.0%
				H-4 →L+1	22.9%
				H-3 → L+2	10.3%
				H-3 → L+1	6.9%
$S_0 \rightarrow S_8$	2.6674	464.81	0.00030	H-4 → L	33.5%
				H-3 → L+1	26.2%
				H-4 → L+1	22.7%
				H-1 → L+1	9.2%
$S_0 \rightarrow S_9$	2.7240	455.15	0.43700	H-1 → L+2	84.1%
				H-2 → L+1	5.1%
$S_0 \rightarrow S_{10}$	2.8298	438.14	0.00400	H-2 → L	75.4%
				H-2 → L+2	10.6%

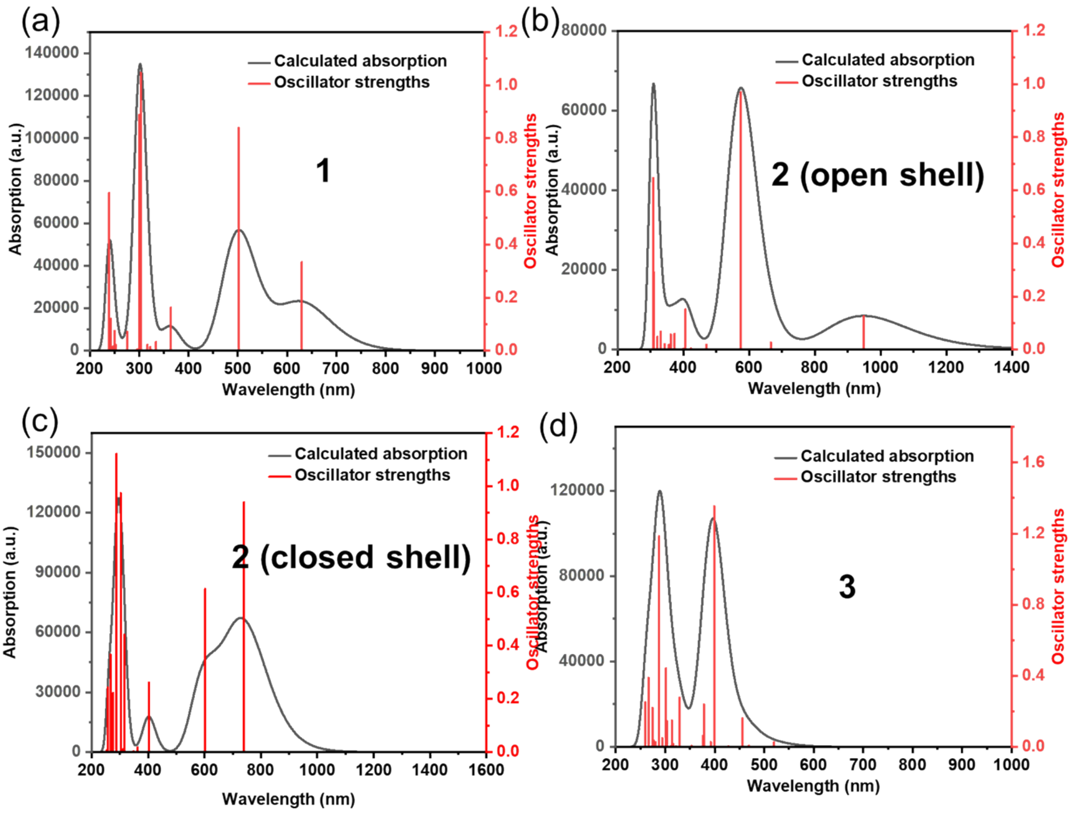

Figure S22 Calculated UV-vis absorption spectra of the (a) **1** with closed shell, (b) **2** with open shell, (c) **2** with closed shell and **3** with closed shell based on (U)M06-2X/6-311G(d,p) level of theory.

Table S11 Excitation energies, oscillator strengths and orbital contribution of the $S_0 \rightarrow S_n$ excitations of **1** calculated at the M06-2X/6-311G(d,p) level of theory.

transition	energy [eV]	energy [nm]	oscillator strength	orbital contribution	
$S_0 \rightarrow S_1$	1.8637	665.26	0.00000	H-1 → L	68.9%
				H-3 → L	29.1%
$S_0 \rightarrow S_2$	1.9678	630.07	0.33420	H → L	81.5%
				H-2 → L	14.8%
$S_0 \rightarrow S_3$	2.1220	584.28	0.00020	H-3 → L	66.4%
				H-1 → L	28.1%
$S_0 \rightarrow S_4$	2.4694	502.08	0.83930	H-2 → L	79.6%
				H → L	14.5%
$S_0 \rightarrow S_5$	3.4061	364.01	0.16300	H-5 → L	72.8%
				H-12 → L	9.4%
				H-8 → L	6.0%
$S_0 \rightarrow S_6$	3.4499	359.38	0.00020	H-4 → L	91.7%
$S_0 \rightarrow S_7$	3.6610	338.66	0.00040	H → L+1	86.1%
$S_0 \rightarrow S_8$	3.7122	333.99	0.03300	H-8 → L	36.2%
				H-5 → L	18.2%
				H-12 → L	15.3%
				H-6 → L	11.0%
				H-3 → L+1	6.7%
$S_0 \rightarrow S_9$	3.8237	324.25	0.00340	H-7 → L	73.6%
				H-9 → L	13.2%
$S_0 \rightarrow S_{10}$	3.8426	322.66	0.01370	H-6 → L	77.4%
				H-12 → L	5.5%

Table S12 Excitation energies, oscillator strengths and orbital contribution of the $S_0 \rightarrow S_n$ excitations of **2** calculated at the UM06-2X/6-311G(d,p) level of theory.

transition	energy [eV]	energy [nm]	oscillator strength	orbital contribution	
$S_0 \rightarrow S_1$	1.2439	996.74	0.00000	α-H → α-L	28.3%
				β-L → β-L	28.3%
				α-H-1 → α-L	11.4%
				β-H-1 → β-L	11.4%
				α-H-2 → α-L	5.1%
				β-H-2 → β-L	5.1%
$S_0 \rightarrow S_2$	1.3065	948.98	0.12550	β-H-1 → β-L	20.1%
				α-H-1 → α-L	20.1%
				β-H → β-L	12.4%
				α-H → α-L	12.4%
				β-H-2 → β-L	8.0%
				α-H-2 → α-L	8.0%
				β-H-3 → β-L	5.3%
				α-H-3 → α-L	5.3%
$S_0 \rightarrow S_3$	1.7102	724.97	0.00000	β-H → β-L	14.1%
				α-H → α-L	14.1%
				β-H-3 → β-L	13.7%
				α-H-3 → α-L	13.7%
				α-H-2 → α-L	11.4%
				β-H-2 → β-L	11.4%
$S_0 \rightarrow S_4$	1.8611	666.19	0.02690	β-H-3 → β-L	37.5%
				α-H-3 → α-L	37.5%
$S_0 \rightarrow S_5$	1.9744	627.96	0.00000	β-H-3 → β-L	27.2%
				α-H-3 → α-L	27.2%
				β-H-2 → β-L	8.8%
				α-H-2 → α-L	8.8%
				β-H-1 → β-L	5.5%
				α-H-1 → α-L	5.5%
$S_0 \rightarrow S_6$	2.1565	574.93	0.96950	β-H → β-L	29.9%
				α-H → α-L	29.9%
				α-H-2 → α-L	8.3%
				β-H-2 → β-L	8.3%
$S_0 \rightarrow S_7$	2.6375	470.08	0.01790	β-H-2 → β-L	14.2%
				α-H-2 → α-L	14.2%
				β-H-1 → β-L	9.5%
				α-H-1 → α-L	9.5%
				α-H → α-L+1	5.1%

				β-H → β-L+1	5.1%
$S_0 \rightarrow S_8$	2.7134	456.93	0.00000	β-H-1 → β-L	22.1%
				α-H-1 → α-L	22.1%
				α-H-2 → α-L	13.8%
				β-H-2 → β-L	13.8%
				α-H-1 → α-L+1	5.5%
				β-H-1 → β-L+1	5.5%
$S_0 \rightarrow S_9$	2.9308	423.04	0.00410	β-H-4 → β-L	29.6%
				α-H-4 → α-L	29.6%
				β-H-2 → β-L	7.1%
				α-H-2 → α-L	7.1%
$S_0 \rightarrow S_{10}$	2.9478	420.60	0.00000	α-H-4 → α-L	39.4%
				β-H-4 → β-L	39.4%

Table S13 Excitation energies, oscillator strengths and orbital contribution of the $S_0 \rightarrow S_n$ excitations of **2** calculated at the M06-2X/6-311G(d,p) level of theory.

transition	energy [eV]	energy [nm]	oscillator strength	orbital contribution	
$S_0 \rightarrow S_1$	1.6757	739.89	0.94060	H → L	83.8%
				H-2 → L	14.1%
$S_0 \rightarrow S_2$	1.7949	690.76	0.00000	H-3 → L	53.7%
				H-1 → L	44.2%
$S_0 \rightarrow S_3$	1.8729	661.99	0.00000	H-1 → L	52.1%
				H-3 → L	42.9%
$S_0 \rightarrow S_4$	2.0580	602.45	0.61380	H-2 → L	81.6%
				H → L	14.3%
$S_0 \rightarrow S_5$	3.0774	402.89	0.26120	H-5 → L	71.9%
				H-8 → L	14.7%
				H-10 → L	5.4%
$S_0 \rightarrow S_6$	3.1351	395.47	0.00000	H-4 → L	85.7%
$S_0 \rightarrow S_7$	3.3081	374.79	0.00010	H → L+1	74.9%
				H → L+2	17.1%
$S_0 \rightarrow S_8$	3.4112	363.46	0.01770	H-8 → L	49.9%
				H-5 → L	21.7%
				H-6 → L	11.0%
				H-10 → L	9.6%
$S_0 \rightarrow S_9$	3.4694	357.36	0.00000	H-9 → L	59.7%
				H-11 → L	12.6%
				H → L+2	12.6%
				H-4 → L	6.5%
$S_0 \rightarrow S_{10}$	3.5608	348.19	0.00260	H-7 → L	89.8%
				H-11 → L	5.4%

Table S14 Excitation energies, oscillator strengths and orbital contribution of the $S_0 \rightarrow S_n$ excitations of **3** calculated at the M06-2X/6-311G(d,p) level of theory.

transition	energy [eV]	energy [nm]	oscillator strength	orbital contribution	
$S_0 \rightarrow S_1$	2.3855	519.74	0.02520	H → L	49.1%
				H → L+2	27.3%
				H → L+1	10.6%
				H-2 → L+3	6.7%
$S_0 \rightarrow S_2$	2.6444	468.86	0.00600	H → L+3	29.1%
				H → L+2	23.5%
				H → L+1	20.2%
				H-2 → L	14.2%
$S_0 \rightarrow S_3$	2.7179	456.18	0.15980	H → L	26.7%
				H → L+1	25.4%
				H → L+2	20.8%
				H-1 → L	7.7%
$S_0 \rightarrow S_4$	2.9013	427.34	0.00310	H → L+3	42.3%
				H → L+1	21.5%
				H → L+2	9.1%
				H-2 → L+2	6.7%
$S_0 \rightarrow S_5$	3.1056	399.23	1.35390	H-1 → L	64.7%
				H → L	7.9%
				H-1 → L+2	6.7%
				H → L+2	5.8%
$S_0 \rightarrow S_6$	3.1596	392.40	0.01210	H-12 → L+1	26.0%
				H-12 → L	13.6%
				H-11 → L+1	10.6%
				H-6 → L+1	9.4%

				H-11 → L	12.3%
				H-11 → L+2	9.9%
				H-12 → L+2	9.8%
$S_0 \rightarrow S_7$	3.1637	391.90	0.02690	H-12 → L	7.4%
				H-5 → L	6.5%
				H-12 → L+1	5.6%
				H-5 → L+2	5.5%
				H-1 → L+1	48.5%
				H-1 → L+2	21.4%
$S_0 \rightarrow S_8$	3.2787	378.15	0.23860	H-1 → L	7.2%
				H-3 → L+1	5.1%
				H-1 → L+2	46.8%
				H-1 → L+1	20.1%
$S_0 \rightarrow S_9$	3.2991	375.81	0.06100	H-2 → L	6.2%
				H-3 → L	5.8%
				H-1 → L+3	35.0%
				H-2 → L	19.5%
$S_0 \rightarrow S_{10}$	3.5106	353.17	0.00470	H-4 → L+1	7.3%
				H-2 → L+2	6.2%
				H → L+1	5.6%

Table S15 Summary of the absorption and electrochemistry parameters

Compounds	λ_{abs}^{peak}(nm)[a]	ε ($10^5M^{-1}cm^{-1}$)[a]	λ_{abs}^{onset} (nm)[a]	E_g^{opt} (eV)[b]	E^{re1} (eV)[c]	E^{re2} (eV)[c]	E^{ox1} (eV)[c]	E^{ox1} (eV)[c]	HOMO (eV)[d]	LUMO (eV)[e]	E_g^{el} (eV)[f]	E_g^{cal} (eV)[g]
**1**	553.00	0.85	907.00	1.36	-0.47	-0.93	1.16	1.39	-5.48	-3.85	1.63	1.92
	521.00	0.74										
	331.00	1.240										
**2**	644.00	0.74	1071.00	1.15	-0.43	-0.72	0.98	1.22	-5.30	-3.89	1.41	1.28
	330.00	0.93										
**3**	537.00	0.26	600.00	2.06	-0.92	-1.45	1.24	1.53	5.56	-3.40	2.16	2.49
	441.00	1.00										
	339.00	1.95										

a. Absorption maxima, molar extinction coefficient and onset absorption in 10^{-5} mol/L dichloromethane solution.

b. Based on the onset absorption ($E_g^{opt} = 1240/\lambda_{abs}^{onset}$).

c. Oxidative and reductive potential based on cyclic voltammograms (CV) with ferrocene as an external standard.

d. Calculated the HOMO and LUMO energy levels based on the reference of Fc/Fc^+.

f. Estimated using the equation: E_g^{el} = LUMO-HOMO.

g. DFT calculation at (U)B3LYP-D3(BJ)/6-31G(d, p) level.

8. The stabilities of 1, 2 and 3

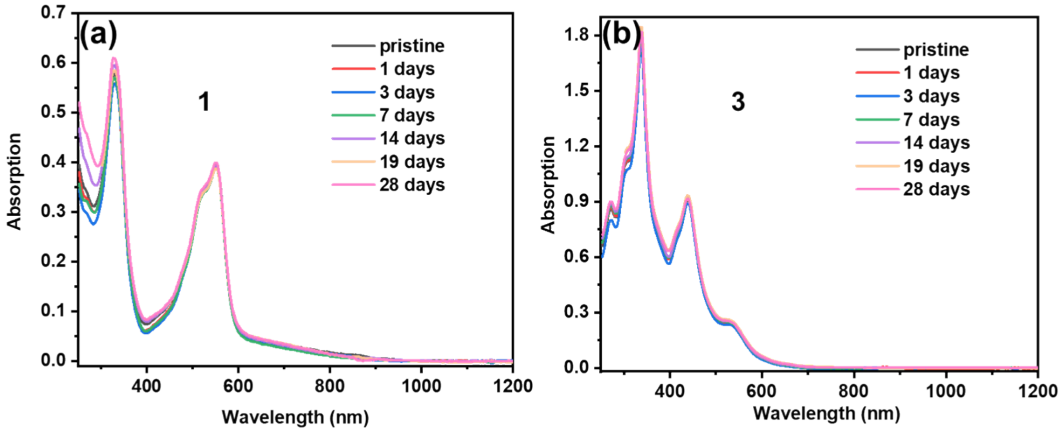

Figure S23 Time-dependent UV-vis measurements were performed under ambient conditions for compound (a) **1** and (b) **3**. Single-crystal samples of **1** and **3** were dissolved in HPLC-grade CH_2Cl_2 open to the atmosphere. The cuvette was sealed with a Teflon cap and kept on the bench top.

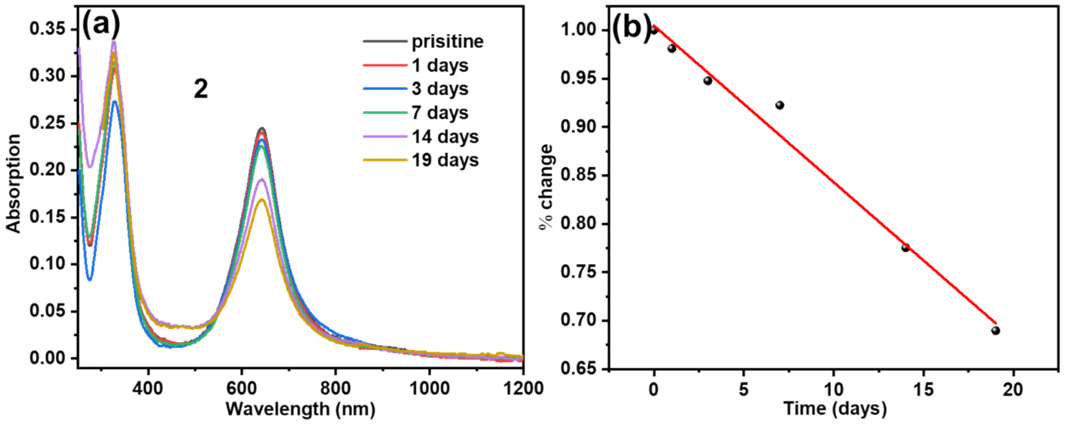

Figure S24 (a) Time-dependent UV−vis measurements were performed under ambient conditions for compound **2**. (b) Time-dependent UV-vis absorbance of **2** in CH_2Cl_2 solution. Linear regression and extrapolation to % change = 0.50 gives a half-life of ca. 32 days.

9. The photoluminescence and photothermal conversion of compound 1 and 2

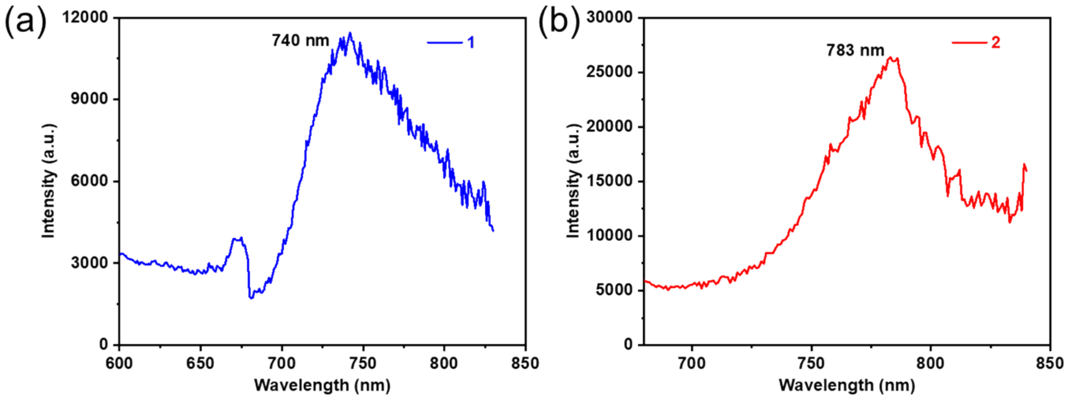

Figure S25 The photoluminescence spectra of (a) **1** and (b) **2**

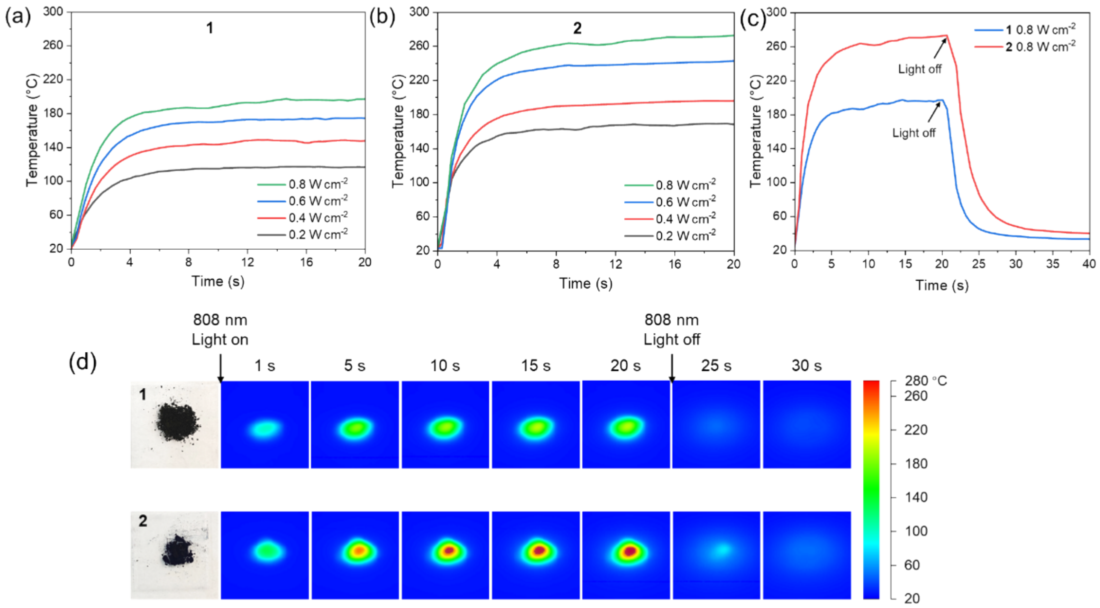

Figure S26 The photothermal conversion of compounds **1** and **2**

10. The oxidation titration experiments

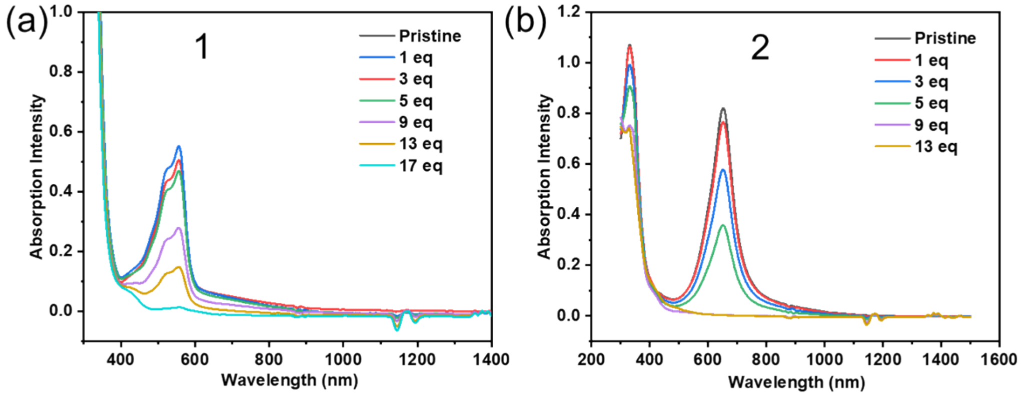

Figure S27 UV-vis-NIR absorption spectra of (a) **1** and (b) **2** by adding NO·SbF_6

11. The variable-temperature ^{1}H NMR

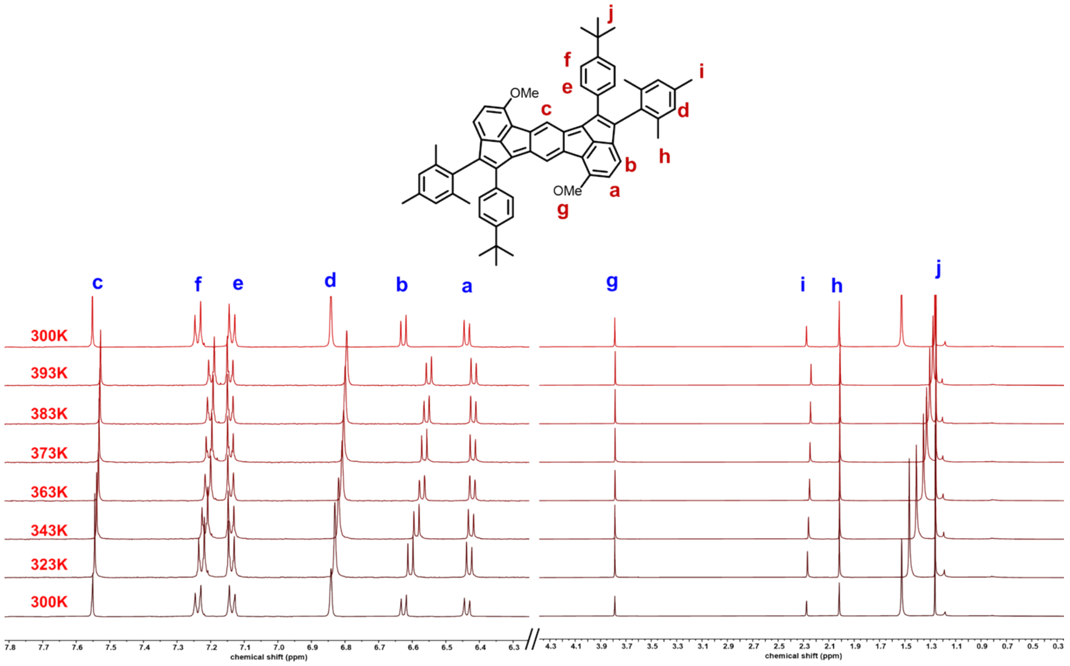

Figure S28 The VT ^{1}H NMR of **1** from 300 K to 393 K and then cooling to 300 K in $C_2D_2Cl_4$ (500 MHz).

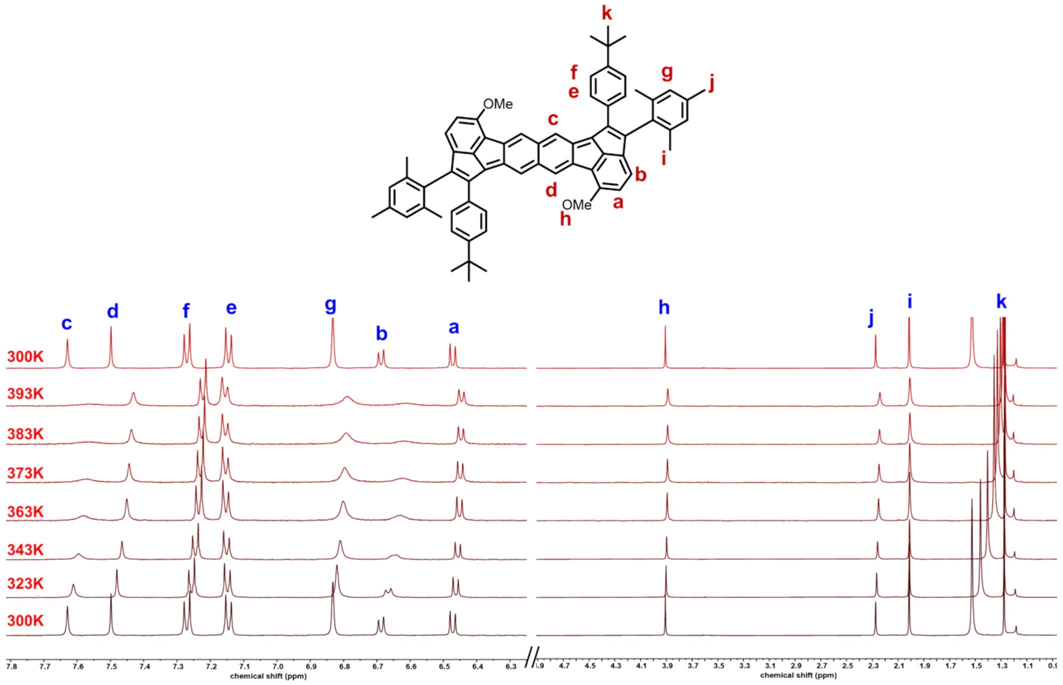

Figure S29 The VT ^{1}H NMR of **2** from 300 K to 393 K and then cooling to 300 K in $C_2D_2Cl_4$ (500 MHz).

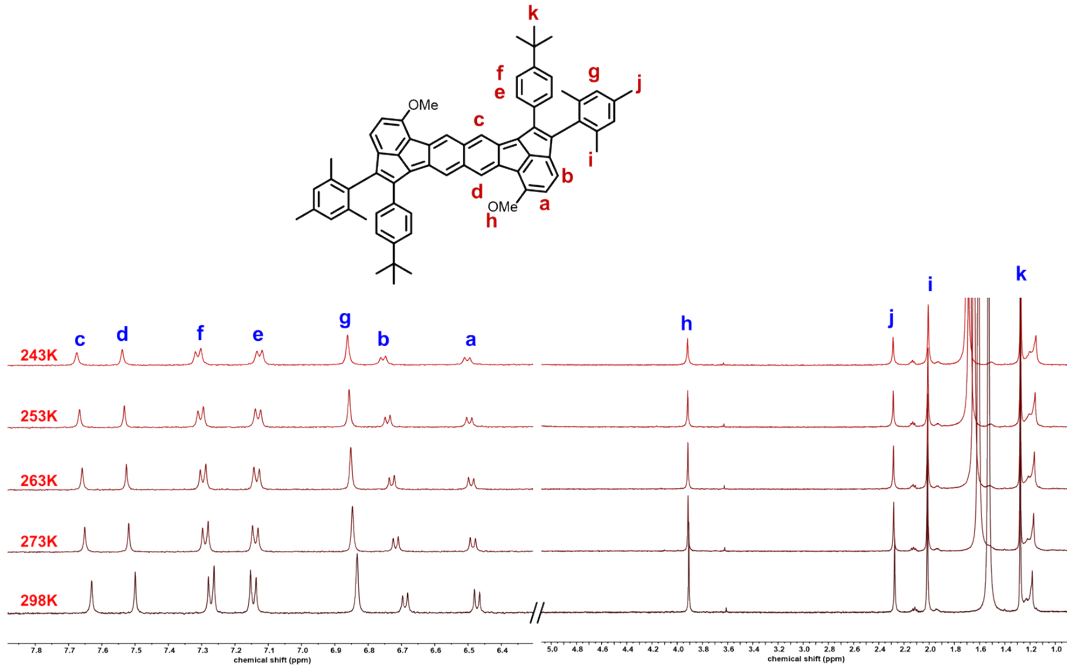

Figure S30 The VT ^1H NMR of **2** from 298 K to 243 K in $C_2D_2Cl_4$ (500 MHz).

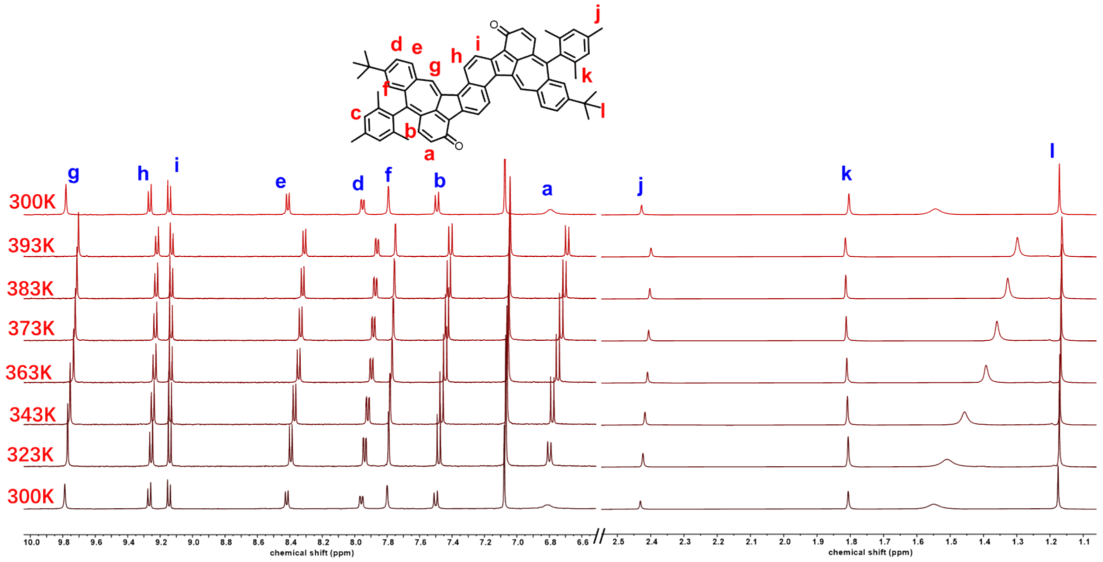

Figure S31 The VT ^1H NMR of **3** from 300 K to 393 K and then cooling to 300 K in $C_2D_2Cl_4$ (500 MHz).

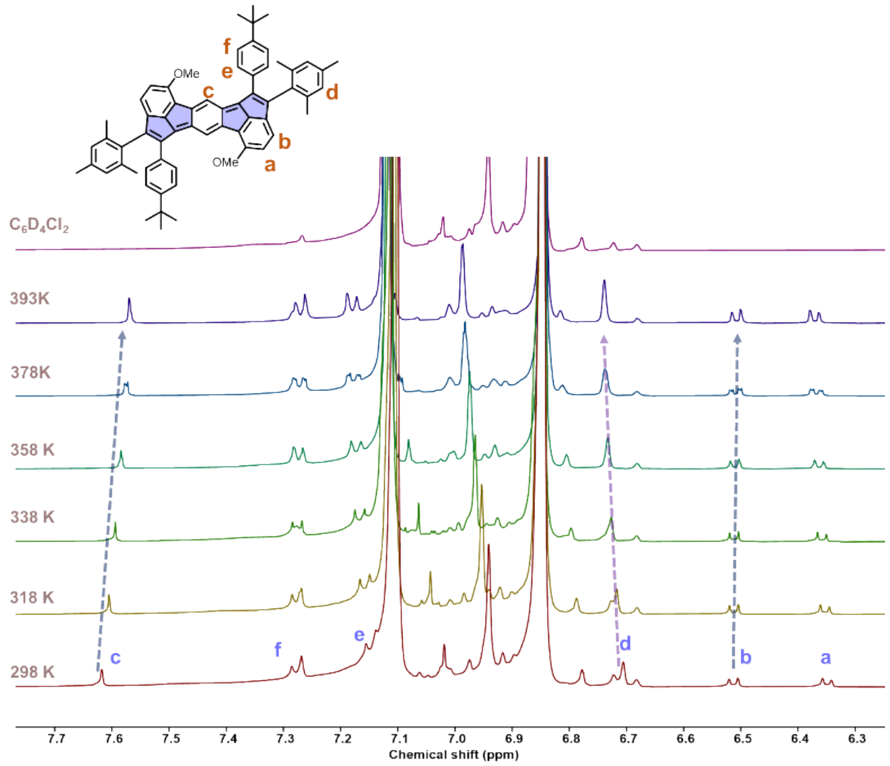

Figure S32 The VT ^{1}H NMR of **1** from 298 to 393 K in $C_6D_4Cl_2$

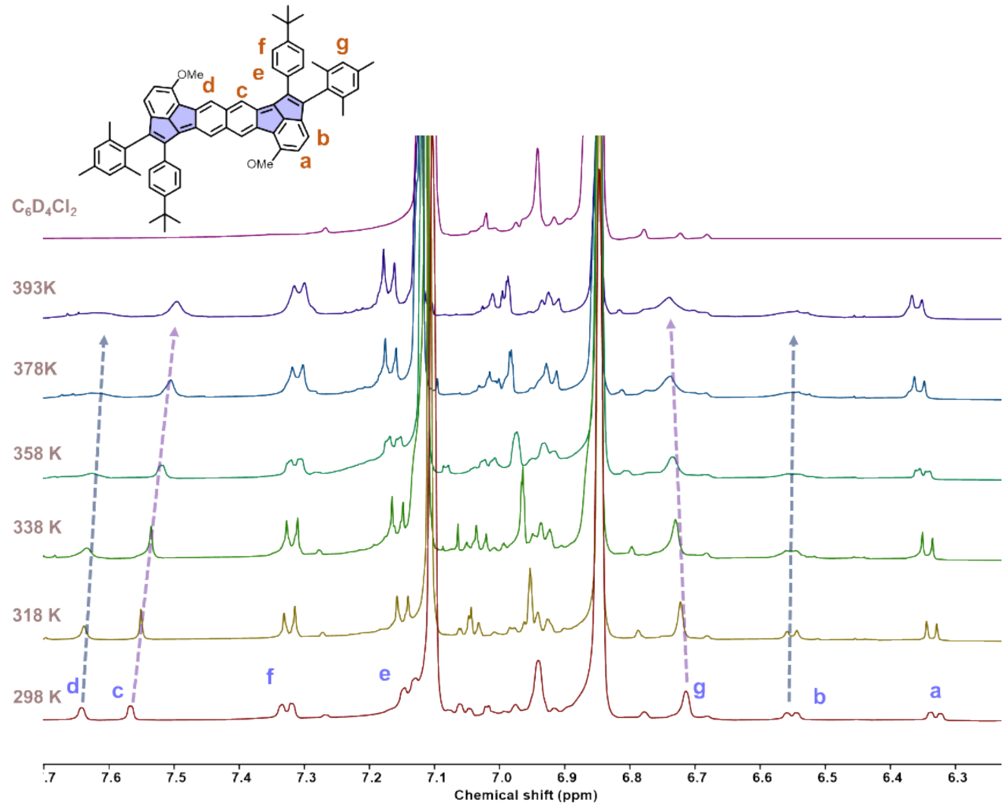

Figure S33 The VT ^{1}H NMR of **2** from 298 to 393 K in $C_6D_4Cl_2$

12. The magnetic properties

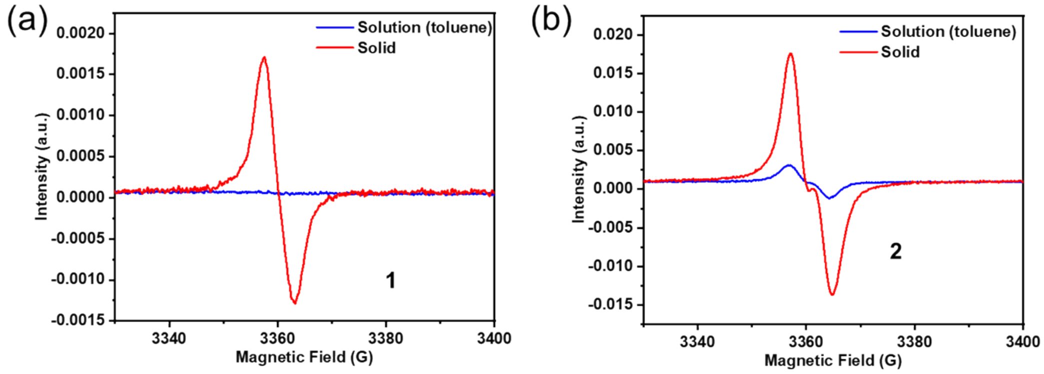

Figure S34 The electron-spin resonance (ESR) spectra of (a) **1** and (b) **2** in toluene solution and powder at room temperature.

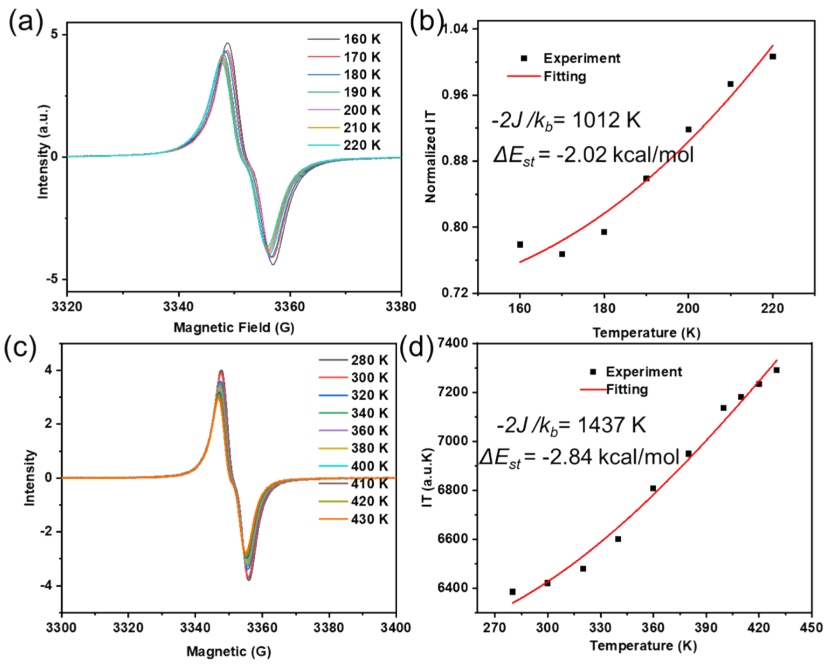

Figure S35 (a, c) Temperature dependence of the ESR signals of **2** in powder state, 160-220 K for (a) and 280-430 K for (c). (b, d) The corresponding integrated signal multiplied by temperature and fitted with the Bleaney-Bowers equation of **2**, 160-220 K for (b) and 280-430 K for (d).

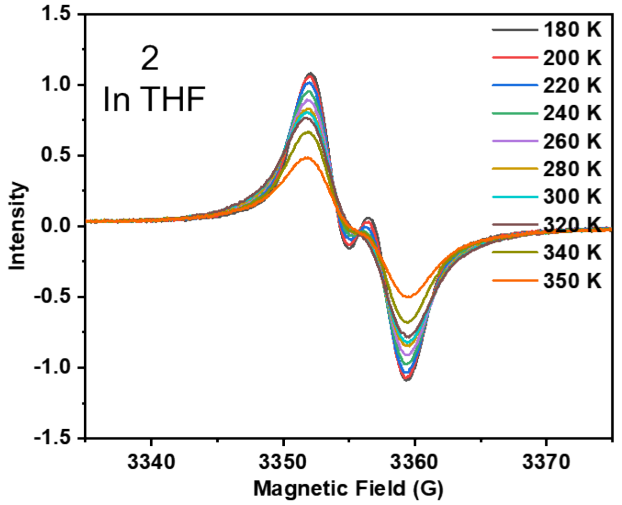

Figure S36 Temperature dependence of the ESR signals of **2** in THF

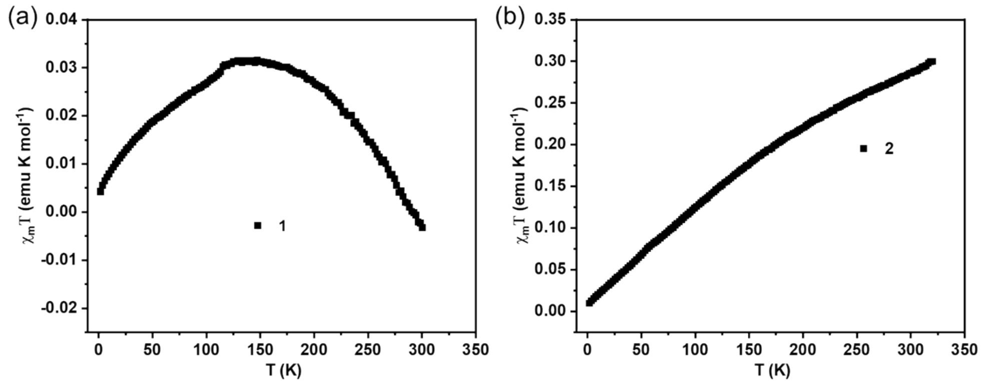

Figure S37 SQUID measurements (black square) of the magnetic susceptibility of (a) **1** and (b) **2** from 2 to 300 K

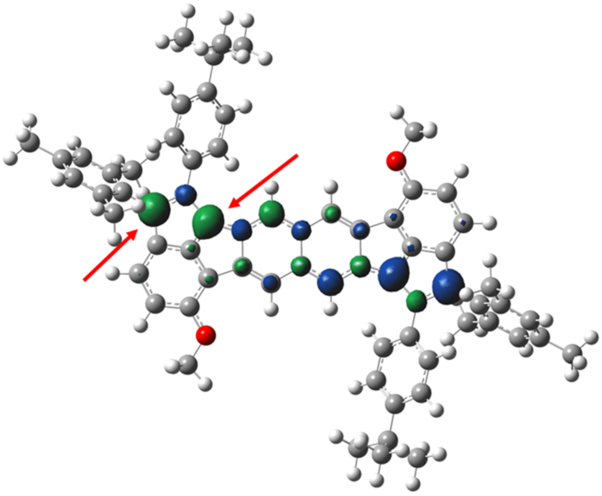

Figure S38 Spin density surfaces calculated by DFT UB3LYP-D3(BJ)/6-31G(d,p) in the open-shell singlet state. The isovalue is 0.005.

Table S16 The ΔE_{st} and y_0 value were calculated based on (U)B3LYP-D3(BJ)/6-31G** and UCAM-B3LYP/6-31G* level, respectively.

Compounds	E_s (Hartree) (Close-Shell)	E_s (Hartree) (Open-Shell)	E_t (Hartree)	y_0 (UCAM-B3LYP/6-31G*)	ΔE_{st} (kcal mol^{-1})
**1**	-2625.795129		-2625.778545		
**2**	-2779.453351	-2779.455595	-2779.447994	0.38	-7.22
**3**	-2699.687238		-2699.636498		

13. OFETs fabrication and characterization

A heavily doped n-type Si wafer with a dry oxidized SiO_2 layer of 300 nm was employed as the gate electrode and dielectric layer. The drain-source (D-S) gold contacts were fabricated by photo-lithography. The substrates were first cleaned by sonication in acetone and water for 5 min, and immersed in Piranha solution (7:3 mixture of sulfuric acid and 30% hydrogen peroxide) for 30 min. The substrates were further rinsed with deionized water and isopropyl alcohol for three times and they were blow-dried with nitrogen. Then, the substrate was processed by UV ozone for about 10 min. After that, the substrates were placed into a petri dish, and one drop of *n*-octadecyltrichlorosilane (OTS) was dropped into the middle of petri dish. The system was stored under vacuum at 120 °C for 4 h to form an OTS self-assembled monolayer. After the substrate surfaces were modified with OTS, they were washed with *n*-hexane, $CHCl_3$ and isopropyl alcohol sequentially. Next, thin films of **2** were prepared by drop-coating of their toluene solutions (8.0 mg/mL, 10 μL) onto the modified substrates, and the substrate was pre-heated at 100 °C. After the solvent dried, the devices can be measured directly under an air atmosphere. The transfer and output curves of FETs were measured at room temperature in an air atmosphere using a Keithley 4200 SCS semiconductor parameter analyzer. The charge mobility of the OFETs in the saturation region was extracted from the following equation:

$$I_{\mathrm{DS}}= \mu C_i(V_{\mathrm{GS}}-V_{\mathrm{Th}})^2\ W/2L$$

Where I_{DS} is the current collected by the drain electrode; L and W are the channel length and width, respectively; μ is the mobility of the device; C_i is the capacitance per unit area of the gate dielectric layer (11 nF/cm^2); V_{GS} is the gate voltage, and V_{Th} is the threshold voltage, respectively. The V_{Th} of the device was obtained by extrapolating the $(I_{\mathrm{DS}},\mathrm{sat})^{1/2}$ vs. V_{GS} plot to $I_{\mathrm{DS}} = 0$.

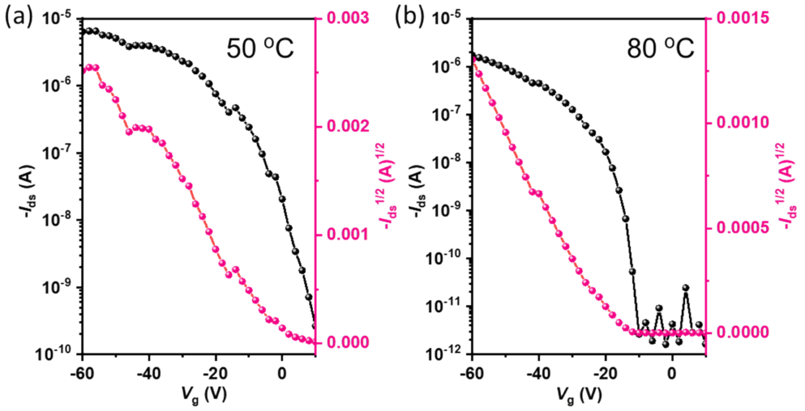

Figure S39 Transfer characteristics of OFETs based on **2** at different thermal annealing temperatures of (a) at 50 °C and (b) at 80 °C

Table S17 The FETs performance of BGBC devices based on the **2** thin films. The width (W) and length (L) of the conductive channel are 1440 μm, and 5 μm, respectively.

$T_{\text{annealing}}$	μ_{max}(cm^{2}V^{-1}s^{-1})[a]	μ_{ave}(cm^{2}V^{-1}s^{-1})[b]	V_{th}(V)[c]	I_{on}/I_{off}[d]
**rt**	0.083	0.064	0.6-3.3	10^3-10^4
**50 °C**	0.0025	0.0021	5.2-8.6	10^3-10^4
**80 °C**	0.000064	0.000058	14.2-18.6	10^5-10^6

[a] maximum hole mobility; [b] average hole mobility; [c] threshold voltage; [d] current on/off ratio. The average mobility was obtained based on 13 FET devices.

14. Atomic Cartesian coordinates of the optimized geometry

Table S13 Cartesian coordinates of the optimized geometry of **1** at the B3LYP-D3(BJ)/6-31G(d,p) level of theory.

C	-0.93951700	1.11391500	-0.06078100
C	0.40351100	1.33770100	-0.05963500
C	1.38557300	0.20680300	-0.07108600
C	0.90832700	-1.14394900	-0.06351900
C	-0.43509700	-1.36819600	-0.06557600
C	-1.41569900	-0.23627300	-0.07391100
C	-1.21312500	-2.62607800	-0.02918100
C	-2.51940400	-2.20320500	-0.02057400
C	-2.69986600	-0.77886300	-0.06295700
C	1.17783000	2.59631900	-0.01879400
C	2.48474700	2.17655600	-0.01273400
C	2.66944900	0.75225600	-0.05853000
C	-3.70309500	-2.90935700	0.06022800
C	-4.74925300	-1.85424200	0.06078700
C	-4.14422700	-0.58728800	-0.01752700
C	3.66537000	2.88739900	0.06820200
C	4.71516500	1.83707700	0.06341000
C	4.11575500	0.56686600	-0.01523400
C	-1.05480500	-4.02179600	0.02366800
C	-2.22311300	-4.82255500	0.08384800
C	-3.53733800	-4.29716600	0.10457700
C	1.01386200	3.99101600	0.04038600
C	2.17902300	4.79591300	0.10178400
C	3.49496300	4.27449500	0.11776900
O	0.21968000	-4.50731900	0.01351900
O	-0.26303500	4.47025300	0.03526200
C	4.80136200	-0.72957200	-0.05758900
C	6.16241500	2.12682900	0.10586400
C	-4.81772100	0.71568000	-0.05595600
C	-6.19830700	-2.13340500	0.10815500
C	-6.75844200	-2.69676600	1.27568400
C	-8.12774100	-2.96441800	1.31148700
C	-8.95836700	-2.69944600	0.21944200
C	-8.38229200	-2.15486400	-0.92989900
C	-7.01869400	-1.86193100	-1.00699500
C	-4.29648500	1.74630400	-0.85823000
C	-4.90008700	2.99595700	-0.90253200
C	-6.05258900	3.28263900	-0.15370600
C	-6.57515300	2.24909000	0.63351700
C	-5.97441700	0.99243700	0.68564300

C	4.28360000	-1.77155300	-0.84092300
C	4.90945400	-3.01535500	-0.89810000
C	6.08323500	-3.27347300	-0.18100500
C	6.60152000	-2.22381200	0.59577700
C	5.98099000	-0.98333800	0.66347800
C	6.98126900	1.86245800	-1.01174300
C	8.34458100	2.15768500	-0.93536700
C	8.92059700	2.70231900	0.21372700
C	8.09234200	2.95415400	1.31099800
C	6.72362100	2.68435700	1.27594600
C	-6.44923000	-1.27233600	-2.27336600
C	-5.90294000	-2.96721700	2.49000500
C	-10.44072800	-2.96924900	0.29143700
C	-6.67818400	4.68107200	-0.22365200
C	-7.10790900	4.97541800	-1.67730500
C	-7.91380600	4.81307300	0.68096400
C	-5.63634000	5.72912500	0.22535200
C	6.41269000	1.26910800	-2.27672000
C	5.87112300	2.93675800	2.49622700
C	10.39081000	3.03631200	0.26250700
C	6.80292900	-4.62729700	-0.21451500
C	6.08063600	-5.64837900	-1.10792200
C	8.23316700	-4.43046300	-0.76228000
C	6.87629400	-5.20300300	1.21633400
C	-0.45898600	5.87286300	0.11469500
C	0.40736400	-5.91186000	0.07626600
H	-1.63853000	1.94200800	-0.03632900
H	1.60717600	-1.97221000	-0.04028400
H	-2.11521500	-5.89967700	0.12138400
H	-4.37750500	-4.98355000	0.15533800
H	2.06730800	5.87251500	0.14375100
H	4.33317200	4.96319900	0.16949700
H	-8.55706300	-3.38712700	2.21687700
H	-9.01035600	-1.95505100	-1.79490300
H	-3.41994500	1.55036800	-1.46676900
H	-4.46418900	3.76037500	-1.53827700
H	-7.46250700	2.41755400	1.23182700
H	-6.40181700	0.22173300	1.31631500
H	3.39101400	-1.59544400	-1.43189200
H	4.46799300	-3.78429400	-1.52068300
H	7.50850700	-2.37884600	1.17179200
H	6.40689600	-0.20384700	1.28384700
H	8.97281100	1.95654900	-1.79987700
H	8.52382500	3.36518500	2.22075400

H	-7.14936300	-1.39150100	-3.10431700
H	-6.24624400	-0.20271200	-2.15423800
H	-5.50279200	-1.74719300	-2.54660400
H	-5.31475900	-2.08505300	2.76363100
H	-6.52092800	-3.24782100	3.34656200
H	-5.18483800	-3.77337000	2.31004800
H	-10.66047300	-3.82555300	0.93591700
H	-10.98003200	-2.10661500	0.70218000
H	-10.85816400	-3.17187500	-0.69905500
H	-7.55291400	5.97392500	-1.74937000
H	-6.25895500	4.93613800	-2.36545400
H	-7.84846400	4.24500500	-2.01751200
H	-8.32304500	5.82491700	0.60145100
H	-7.66606900	4.63598000	1.73217700
H	-8.70334000	4.11245100	0.39202400
H	-4.74862600	5.71562400	-0.41355600
H	-6.06516600	6.73626600	0.18202100
H	-5.31247200	5.53799600	1.25316000
H	5.46205800	1.73731900	-2.54675300
H	7.10946200	1.39404900	-3.10963900
H	6.21782200	0.19796600	-2.15765800
H	6.49001500	3.21846200	3.35176500
H	5.14337400	3.73609500	2.32483000
H	5.29385100	2.04639500	2.76668800
H	10.96457200	2.41578700	-0.43169900
H	10.56800700	4.08355400	-0.01215200
H	10.79977300	2.89174600	1.26701000
H	6.62824300	-6.59583600	-1.10231800
H	5.06483400	-5.84930100	-0.75263400
H	6.01909300	-5.30648900	-2.14574200
H	8.76701600	-5.38670600	-0.78887300
H	8.81122700	-3.74059600	-0.14150000
H	8.20662600	-4.02436300	-1.77822200
H	5.87263100	-5.34640500	1.62848800
H	7.38762400	-6.17194600	1.21172400
H	7.42314700	-4.53951000	1.89179000
H	-1.53896100	6.02615700	0.10903000
H	-0.03844100	6.28562800	1.04002000
H	-0.01320600	6.39041600	-0.74387300
H	1.48616900	-6.07194700	0.06134600
H	-0.00956900	-6.33238100	0.99974900
H	-0.04716300	-6.41711200	-0.78500400

Table S14 Cartesian coordinates of the optimized geometry of **2** at the UB3LYP-D3(BJ)/6-31G(d,p) level of theory.

C	-1.71686500	-1.87235500	0.02868600
C	-2.47221400	-0.59730700	0.01217600
C	-1.76904900	0.60954100	0.01740100
C	-0.36640000	0.62435400	0.02320700
C	0.36639800	-0.62434300	0.02320200
C	-0.35302600	-1.86418400	0.03196200
C	0.35302400	1.86419500	0.03197400
C	1.71686300	1.87236600	0.02869800
C	2.47221200	0.59731900	0.01218000
C	1.76904700	-0.60952900	0.01739600
C	3.86600600	0.91467000	0.00987600
C	-3.86600800	-0.91465900	0.00987200
C	-2.69761600	-2.97671700	0.05523300
C	2.69761400	2.97672800	0.05525100
C	5.23833300	0.48600100	0.02424900
C	-5.23833600	-0.48599000	0.02424400
C	2.77424800	4.38323500	0.09544800
C	4.05655900	4.98155200	0.12578200
C	5.26780500	4.24604000	0.12622800
C	5.20475200	2.85122800	0.09846100
C	3.91688400	2.34273700	0.05170900
C	-2.77425100	-4.38322500	0.09541400
C	-4.05656200	-4.98154200	0.12573900
C	-5.26780800	-4.24602900	0.12618800
C	-5.20475500	-2.85121700	0.09843300
C	-3.91688600	-2.34272600	0.05169400
C	-6.06245100	-1.64789100	0.07627400
C	6.06244800	1.64790000	0.07630300
C	-5.70351900	0.90472900	-0.02201100
C	5.70351600	-0.90471700	-0.02202300
C	-6.84077900	1.34101000	0.67926000
C	-7.25985300	2.66272400	0.60126600
C	-6.57313700	3.61621000	-0.16844800
C	-5.43914200	3.17781800	-0.86207600
C	-5.01416900	1.85225100	-0.79298900
C	5.01417800	-1.85222500	-0.79302800
C	5.43915300	-3.17779000	-0.86213500
C	6.57314100	-3.61619400	-0.16850000
C	7.25984500	-2.66272100	0.60124000
C	6.84076800	-1.34101000	0.67925400
C	-7.07936400	5.06285100	-0.22273300

C	-7.08843700	5.65567400	1.20300700
C	-6.19730600	5.95606900	-1.10981300
C	-8.51399300	5.07885900	-0.79383400
C	7.07937100	-5.06283300	-0.22280800
C	6.19732900	-5.95603400	-1.10992000
C	8.51400900	-5.07882500	-0.79388800
C	7.08842400	-5.65568500	1.20292000
O	-1.59781700	-5.07133800	0.10156500
C	-1.64761600	-6.48895800	0.14638500
O	1.59781400	5.07134800	0.10160300
C	1.64761200	6.48896600	0.14648400
C	-7.53722100	-1.68039000	0.06470400
C	-8.25604100	-1.25342700	-1.07255400
C	-9.65151800	-1.30368500	-1.04857800
C	-10.35694700	-1.75981200	0.06667600
C	-9.62719900	-2.17572700	1.18342800
C	-8.23180800	-2.15181400	1.20083100
C	-7.48527500	-2.57313500	2.44398900
C	-7.54711300	-0.75032100	-2.30531800
C	-11.86369000	-1.82895300	0.05646200
C	7.53721800	1.68038900	0.06474000
C	8.25604000	1.25340700	-1.07251000
C	9.65151800	1.30363900	-1.04852200
C	10.35694700	1.75975500	0.06673500
C	9.62719600	2.17568900	1.18348100
C	8.23180600	2.15180300	1.20087300
C	7.48527300	2.57312400	2.44403100
C	7.54711900	0.75029500	-2.30527600
C	11.86369100	1.82886900	0.05653400
H	-2.30765500	1.55187500	0.02493300
H	0.20244000	-2.79692500	0.04457000
H	-0.20244200	2.79693700	0.04458800
H	2.30765300	-1.55186400	0.02492200
H	4.12823600	6.06181700	0.15260400
H	6.20973600	4.78646500	0.14862700
H	-4.12823900	-6.06180700	0.15255300
H	-6.20973900	-4.78645400	0.14857800
H	-7.39437300	0.63851100	1.29077100
H	-8.14202500	2.95838000	1.16064400
H	-4.87516900	3.86805800	-1.47797700
H	-4.14978400	1.53614400	-1.36802500
H	4.14980100	-1.53610900	-1.36807000
H	4.87519000	-3.86801900	-1.47805600
H	8.14201000	-2.95838700	1.16062400

H	7.39435300	-0.63852100	1.29078500
H	-7.45291700	6.68860600	1.18414400
H	-7.73553100	5.08459400	1.87426100
H	-6.08058100	5.65554700	1.62962600
H	-6.59480400	6.97562900	-1.11673300
H	-6.17405800	5.60196400	-2.14514000
H	-5.16823300	6.00317100	-0.73997900
H	-8.53277700	4.66418000	-1.80649400
H	-9.19990400	4.48987700	-0.17889200
H	-8.89675700	6.10449400	-0.83591500
H	6.59482900	-6.97559300	-1.11685500
H	5.16825000	-6.00314600	-0.74010300
H	6.17409600	-5.60190900	-2.14524000
H	8.89677600	-6.10445800	-0.83598400
H	9.19990900	-4.48985500	-0.17892400
H	8.53280700	-4.66412600	-1.80653800
H	6.08056200	-5.65557000	1.62952400
H	7.45290700	-6.68861700	1.18404100
H	7.73550700	-5.08461900	1.87419600
H	-0.61085700	-6.82707600	0.14743200
H	-2.14713000	-6.84394300	1.05617200
H	-2.16360100	-6.90003200	-0.73001300
H	0.61085200	6.82708300	0.14755800
H	2.14713700	6.84391200	1.05627900
H	2.16358500	6.90007900	-0.72990400
H	-10.20180600	-0.97837900	-1.92833600
H	-10.15782800	-2.52098000	2.06768700
H	-8.17656300	-2.74460800	3.27291600
H	-6.91311900	-3.49222400	2.28374900
H	-6.76301700	-1.80918500	2.75011300
H	-6.67659500	-1.36660100	-2.54647200
H	-8.22122200	-0.75075000	-3.16583900
H	-7.18364500	0.27273100	-2.16222200
H	-12.29405400	-1.06587600	-0.59852400
H	-12.21249300	-2.80391300	-0.30607900
H	-12.27664600	-1.68950000	1.05988500
H	10.20180600	0.97831900	-1.92827500
H	10.15782500	2.52093400	2.06774300
H	8.17656800	2.74466600	3.27293700
H	6.91305600	3.49217000	2.28377200
H	6.76306900	1.80913800	2.75019700
H	7.18376600	-0.27280400	-2.16221700
H	6.67652800	1.36649400	-2.54636800
H	8.22119800	0.75083700	-3.16582000

H	12.21251500	2.80381700	-0.30601700
H	12.27663500	1.68942000	1.05996300
H	12.29404600	1.06577500	-0.59843800

Table S15 Cartesian coordinates of the optimized geometry of **3** at the B3LYP-D3(BJ)/6-31G(d,p) level of theory.

O	-2.62381700	-4.76384800	-0.68004000
O	2.61480100	4.74845100	-0.70276000
C	-3.98691200	-1.41450500	-0.25219600
C	-3.24366400	-0.15465400	-0.10856500
C	-3.06402900	-2.43866900	-0.40566000
C	3.23339600	0.14234000	-0.10923400
C	-1.81953500	-0.47784100	-0.26928100
C	1.80854300	0.46591200	-0.27113300
C	1.71727600	1.86391000	-0.40883200
C	0.63406400	-0.34436000	-0.32749700
C	0.66216800	-1.76254100	-0.46679900
H	1.61346900	-2.25718900	-0.60518100
C	3.75470500	-1.05628000	0.27240700
H	3.03896200	-1.83613800	0.49947000
C	3.97684500	1.40129900	-0.25935100
C	-0.64494600	0.33276600	-0.32895200
C	-1.72777700	-1.87671800	-0.40080300
C	5.11461500	-1.48403700	0.42714800
C	5.39458600	1.64366300	-0.23466600
C	6.31298800	-0.71798900	0.22171700
C	-5.40391300	-1.65598400	-0.22589700
C	-4.88850300	-4.06530900	-0.55101000
H	-5.21598800	-5.09359100	-0.66898200
C	-3.76676100	1.04564000	0.26725700
H	-3.05151100	1.82701800	0.49069300
C	3.05450500	2.42474200	-0.41716400
C	-5.78166800	-3.06163800	-0.40569900
H	-6.83800700	-3.29548400	-0.40780300
C	-5.12637700	1.47222400	0.41918000
C	-6.42333500	-0.72036800	-0.06265800
C	6.41305400	0.71032000	-0.06518400
C	-3.43458100	-3.83680300	-0.55577900
C	0.47393800	2.50835300	-0.53784400
H	0.44713200	3.58056000	-0.68678100
C	7.82501600	1.22666100	-0.16190300
C	-7.83376200	-1.24193500	-0.15827800
C	-0.67287200	1.75007100	-0.47369600
H	-1.62401900	2.24460200	-0.61357200
C	-6.32329900	0.70784300	0.21626200
C	7.66076200	-2.76000900	0.68726100
C	-0.48412100	-2.52141900	-0.52748100

H	-0.45727000	-3.59423000	-0.67200600
C	7.53848700	-1.41908800	0.34951200
H	8.44610300	-0.86223700	0.17855800
C	3.42585800	3.82237100	-0.57475400
C	-5.26526200	2.83960400	0.77462200
H	-4.35745200	3.41187300	0.93946700
C	5.25421600	-2.84663800	0.78632300
H	4.34692400	-3.41981000	0.95108100
C	8.50980400	1.62716800	1.00055300
C	-7.55534200	1.41006200	0.33734900
H	-8.45692100	0.84864400	0.16297000
C	8.47152200	1.24792600	-1.41079100
C	-7.67471700	2.74826600	0.66883300
C	5.77263300	3.04819000	-0.42304300
H	6.82929000	3.28108600	-0.42749900
C	-8.47666800	-1.27885200	-1.40860200
C	9.80422500	1.66453000	-1.47525600
H	10.30515200	1.67411400	-2.44013300
C	-6.48384200	3.46559400	0.90250300
H	-6.51564600	4.51527100	1.17474700
C	9.06061400	-3.37546100	0.79645200
C	6.47539900	-3.47670200	0.91974900
H	6.50170600	-4.52379200	1.19502100
C	4.87972500	4.05116200	-0.57315400
H	5.20690200	5.07877000	-0.69748600
C	-8.51701100	-1.64300700	1.00480700
C	-9.84171700	-2.07257900	0.89614600
H	-10.36967800	-2.37998300	1.79561300
C	9.84017400	2.03957200	0.89287400
H	10.36946900	2.34601100	1.79184400
C	10.50633800	2.06438600	-0.33577100
C	-9.80385500	-1.71224200	-1.47398800
H	-10.30198400	-1.73393400	-2.44013400
C	-7.75019600	-0.84673000	-2.65804800
H	-8.41268400	-0.87663300	-3.52619200
H	-6.89202100	-1.49555400	-2.86246500
H	-7.36064400	0.17206700	-2.55980200
C	-10.50395400	-2.11396900	-0.33404200
C	7.74385800	0.81466100	-2.65915400
H	6.89081600	1.46867400	-2.86822800
H	7.34697900	-0.20080200	-2.55605600
H	8.40798600	0.83497600	-3.52630000
C	-7.83282700	-1.60144600	2.34822100
H	-7.50303200	-0.58631000	2.59399500

H	-6.94067700	-2.23622800	2.35947000
H	-8.50372600	-1.94270300	3.13997600
C	9.85905800	-2.62451200	1.88542900
H	9.95926300	-1.56068500	1.65277000
H	10.86600100	-3.04602300	1.97305500
H	9.36638700	-2.71153700	2.85892500
C	7.82259500	1.59972300	2.34278800
H	8.49390800	1.94297100	3.13329000
H	7.48739400	0.58807400	2.59542900
H	6.93388600	2.23927500	2.34732100
C	-9.03464900	3.44227900	0.78831200
C	9.78517200	-3.23116500	-0.56010300
H	9.23368200	-3.74632400	-1.35273700
H	10.78762100	-3.66813100	-0.50105400
H	9.89355200	-2.18278800	-0.85166300
C	9.00848700	-4.86571900	1.16731500
H	8.52701700	-5.02554500	2.13720900
H	10.02511200	-5.26428900	1.23331000
H	8.47101000	-5.45194500	0.41523600
C	11.93542700	2.53741500	-0.43124400
H	12.44370800	2.09999600	-1.29509000
H	12.50319800	2.27717100	0.46688500
H	11.98315700	3.62764500	-0.54123800
C	-11.92679100	-2.60534800	-0.43029000
H	-11.96098700	-3.69684600	-0.53256300
H	-12.43781500	-2.18022900	-1.29864200
H	-12.50036800	-2.34550100	0.46426900
C	-10.20491200	2.48683900	0.50080300
H	-10.15956400	2.08094900	-0.51450100
H	-11.15064800	3.02763900	0.60069200
H	-10.22644500	1.64552900	1.20038600
C	-9.19453900	3.99339900	2.22183000
H	-9.15622100	3.18307600	2.95636200
H	-10.15761200	4.50410300	2.32522500
H	-8.40810000	4.71110300	2.47157300
C	-9.09536400	4.60842400	-0.22213100
H	-8.30765300	5.34514600	-0.04086500
H	-10.05855200	5.12381400	-0.14721400
H	-8.98253700	4.24070700	-1.24659300

15. ^{1}H NMR and ^{13}C NMR spectra

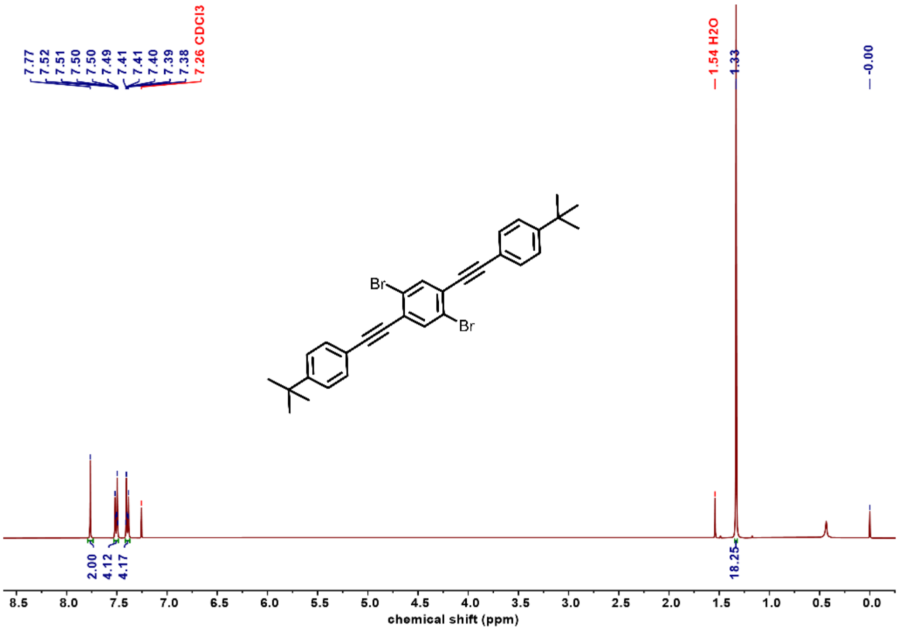

Figure S40 ^{1}H NMR spectrum of compound **5** (400 MHz, $CDCl_3$, 298 K)

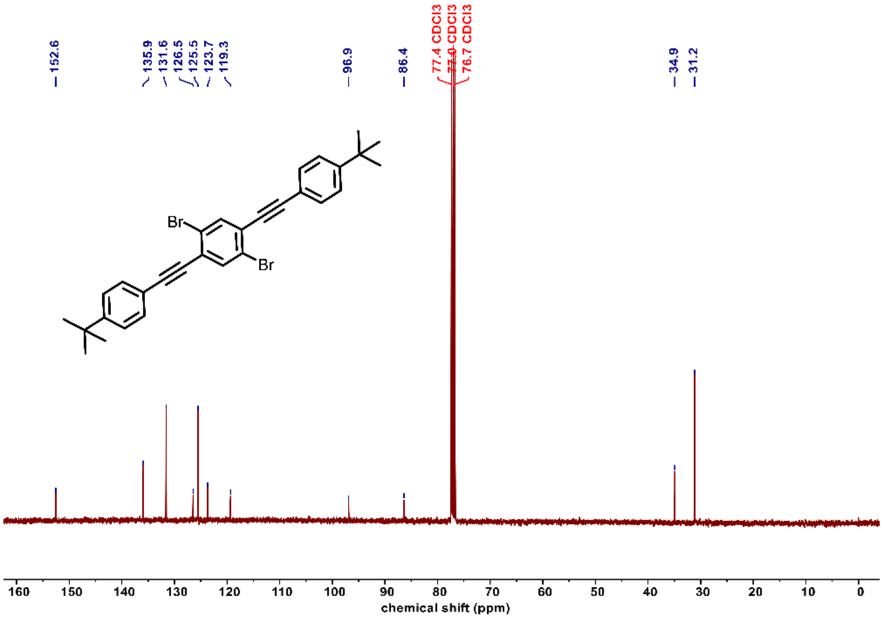

Figure S41 ^{13}C NMR spectrum of compound **5** (101 MHz, $CDCl_3$, 298 K)

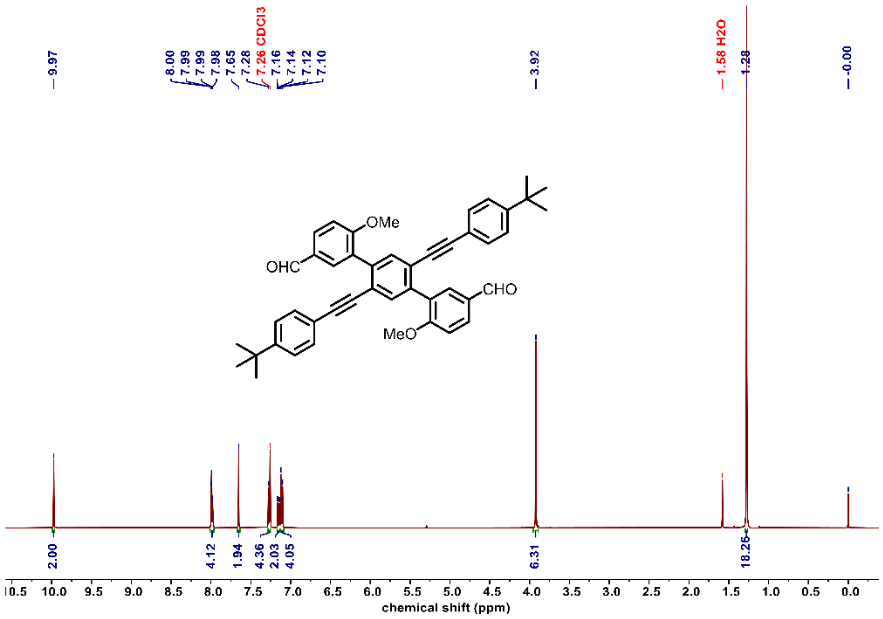

Figure S42 ^{1}H NMR spectrum of compound **6** (400 MHz, $CDCl_3$, 298 K)

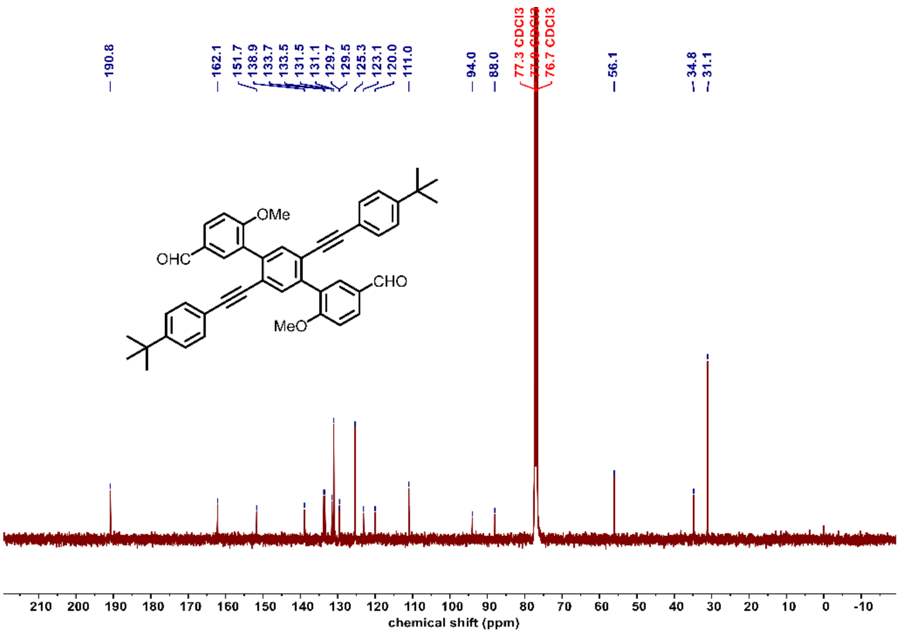

Figure S43 ^{13}C NMR spectrum of compound **6** (101 MHz, $CDCl_3$, 298 K)

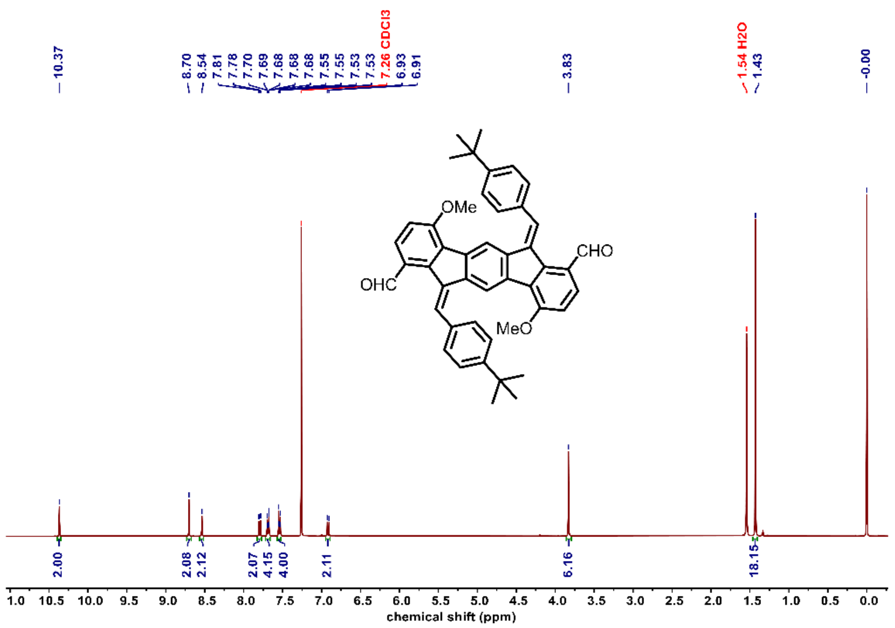

Figure S44 ^{1}H NMR spectrum of compound **7** (400 MHz, $CDCl_3$, 298 K)

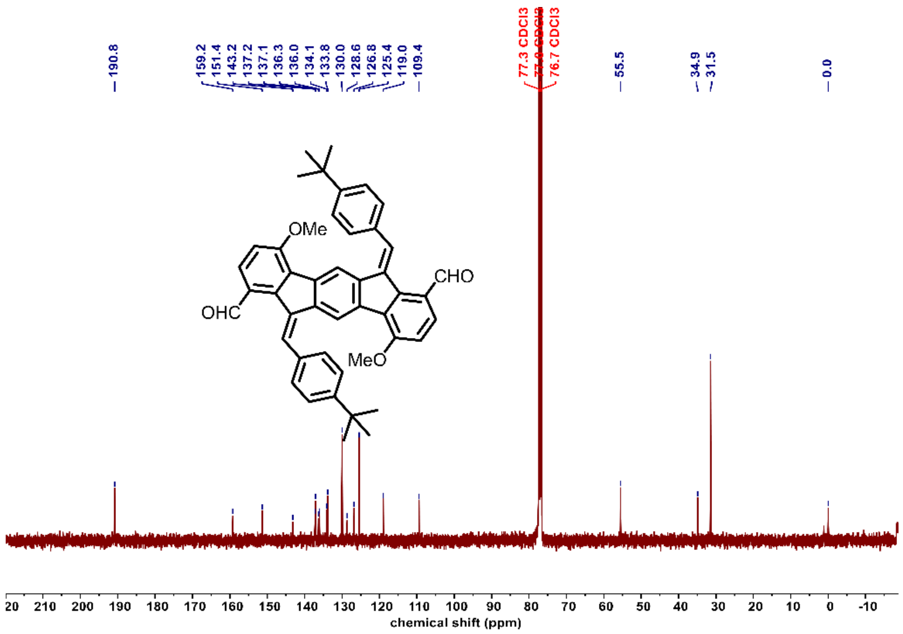

Figure S45 ^{13}C NMR spectrum of compound **7** (101 MHz, $CDCl_3$, 298 K)

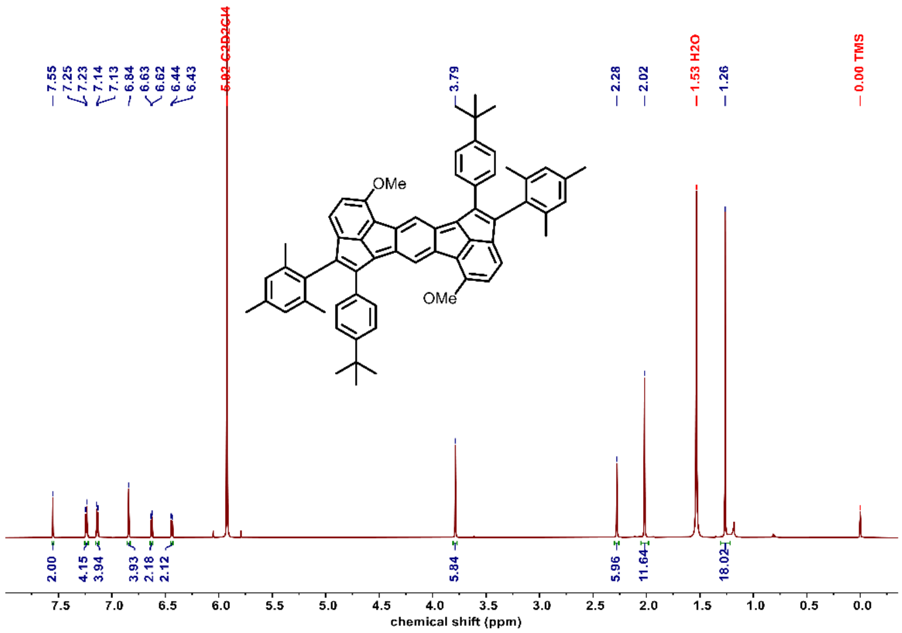

Figure S46 ^{1}H NMR spectrum of compound **1** (700 MHz, $C_2D_2Cl_4$, 298 K)

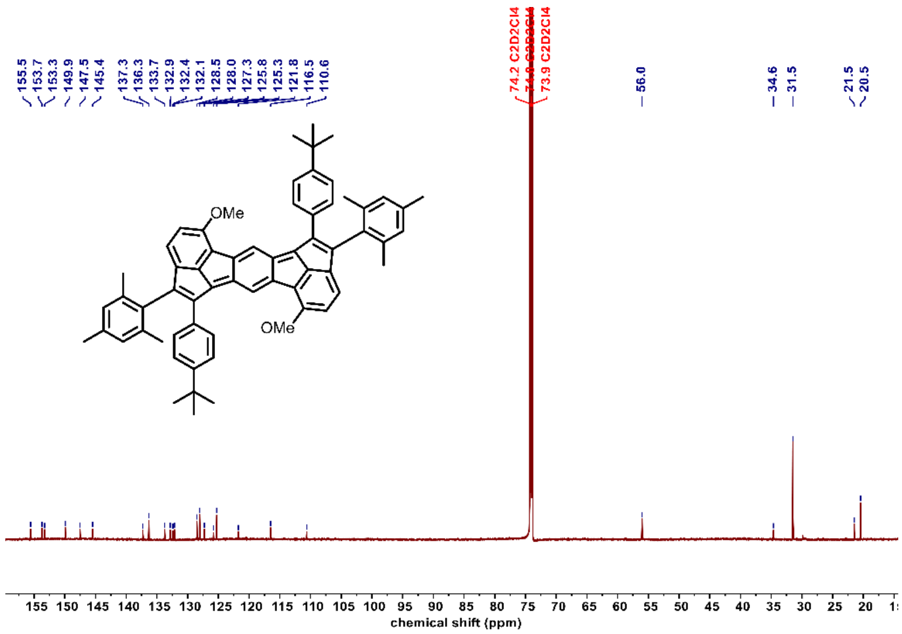

Figure S47 ^{13}C NMR spectrum of compound **1** (176 MHz, $CDCl_3$, 298 K)

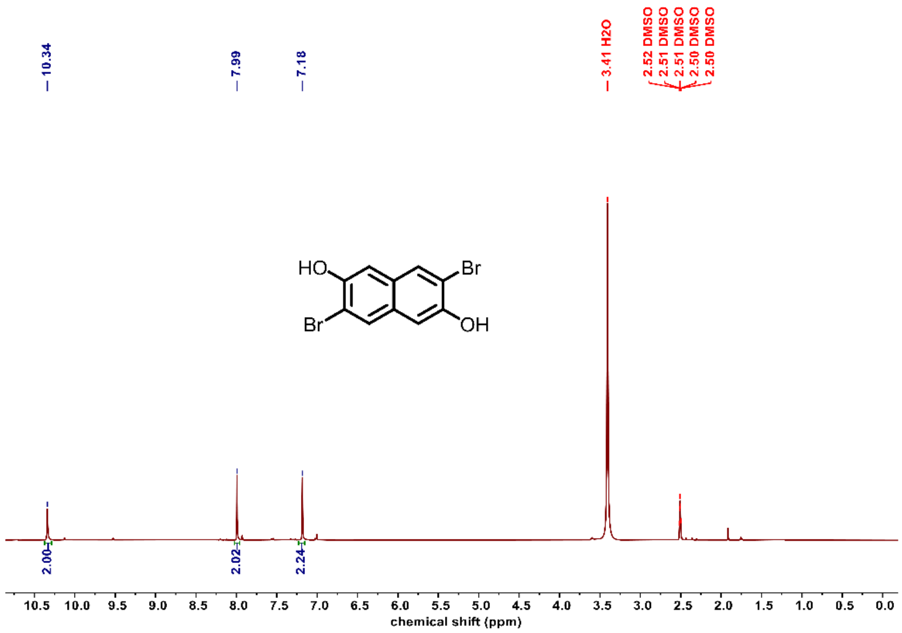

Figure S48 ^{1}H NMR spectrum of compound **Na-2Br-2OH** (400 MHz, DMSO-d_6, 298 K)

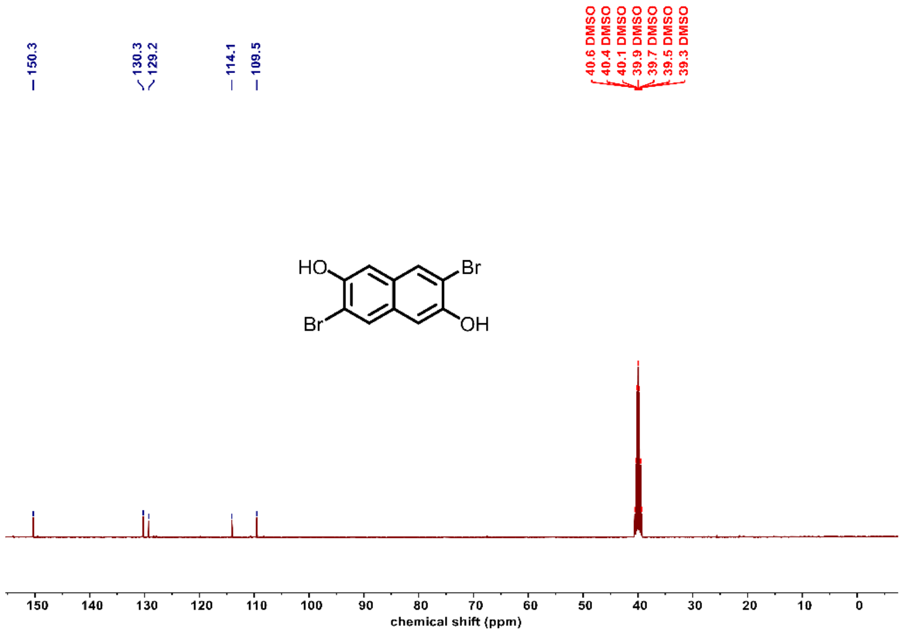

Figure S49 ^{13}C NMR spectrum of compound **Na-2Br-2OH** (101 MHz, DMSO-d_6, 298 K)

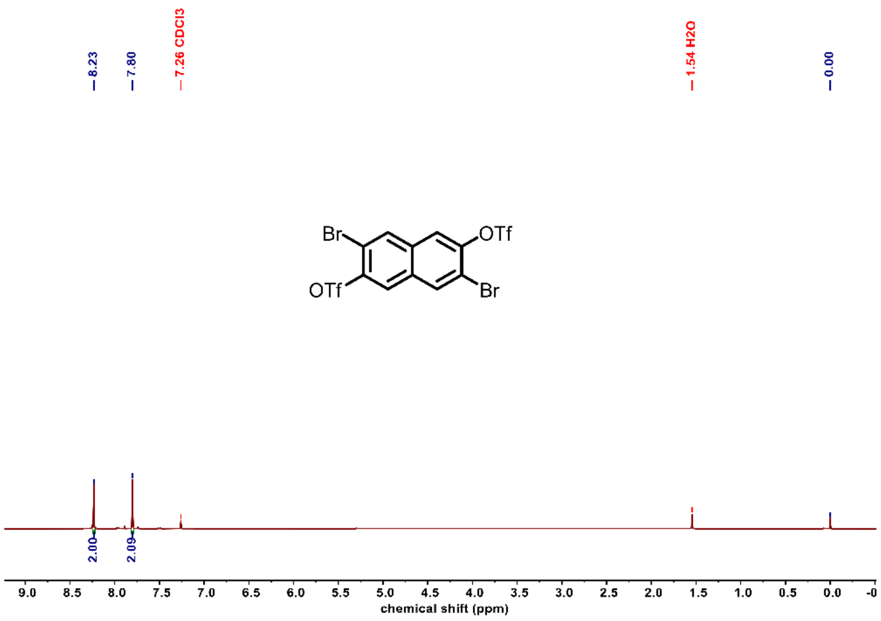

Figure S50 ^{1}H NMR spectrum of compound **10** (400 MHz, $CDCl_3$, 298 K)

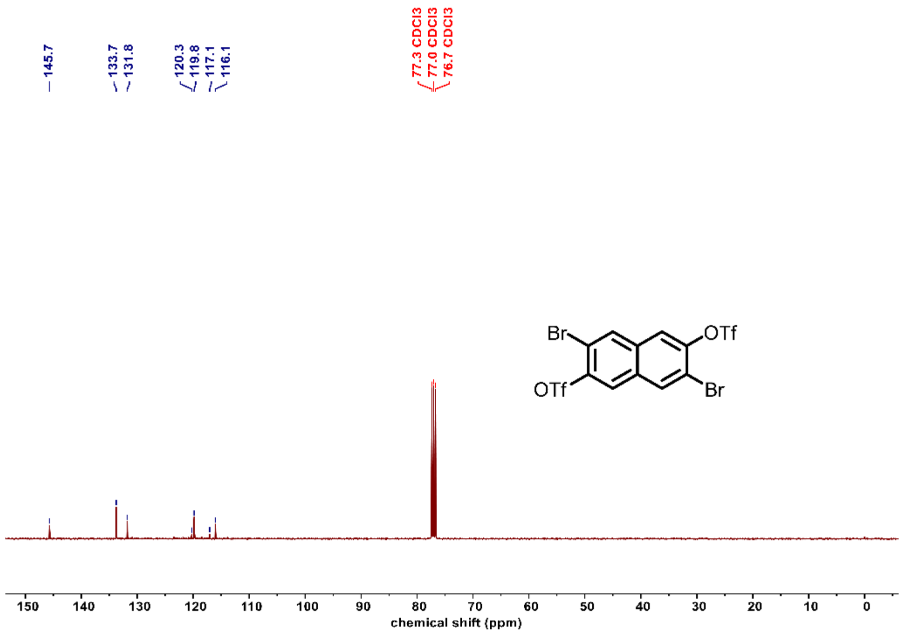

Figure S51 ^{13}C NMR spectrum of compound **10** (101 MHz, $CDCl_3$, 298 K)

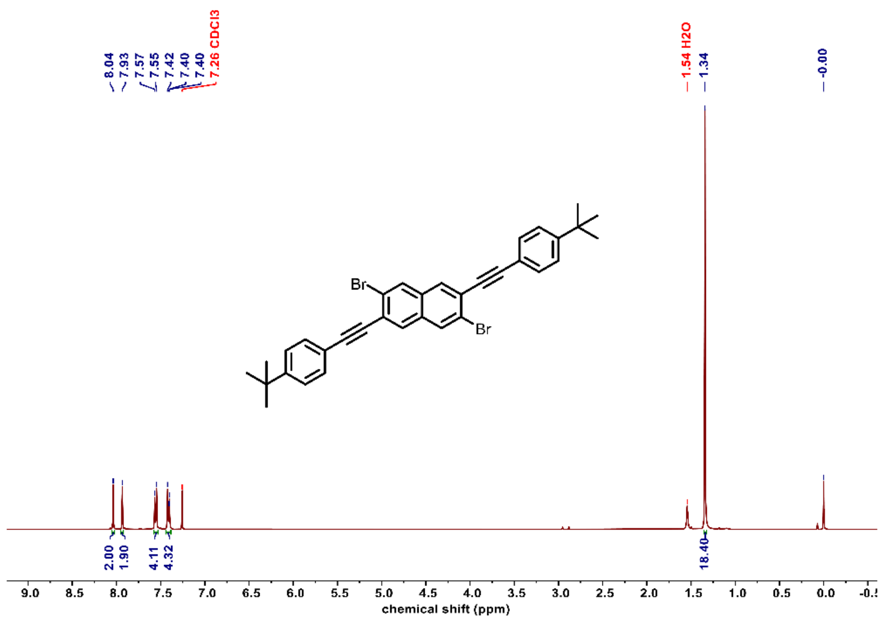

Figure S52 ^{1}H NMR spectrum of compound **11** (400 MHz, $CDCl_3$, 298 K)

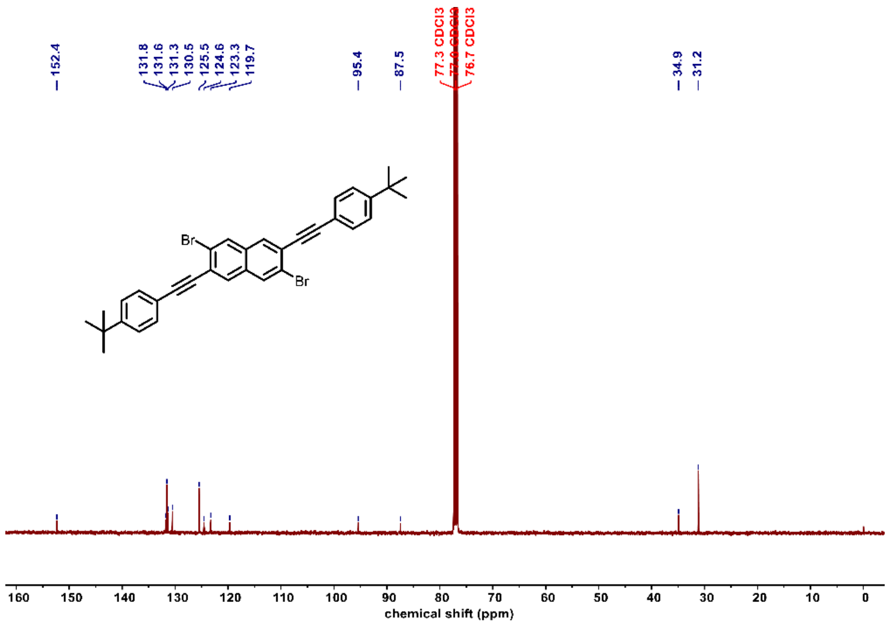

Figure S53 ^{13}C NMR spectrum of compound **11** (101 MHz, $CDCl_3$, 298 K)

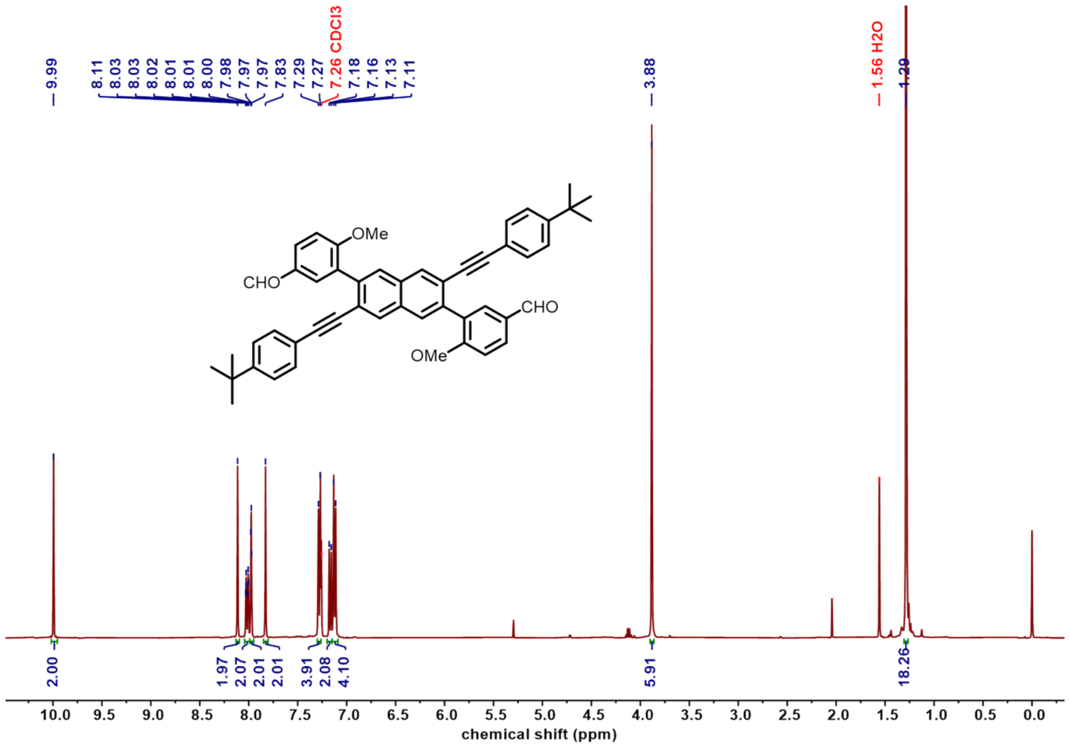

Figure S54 ^{1}H NMR spectrum of compound **12** (400 MHz, $CDCl_3$, 298 K)

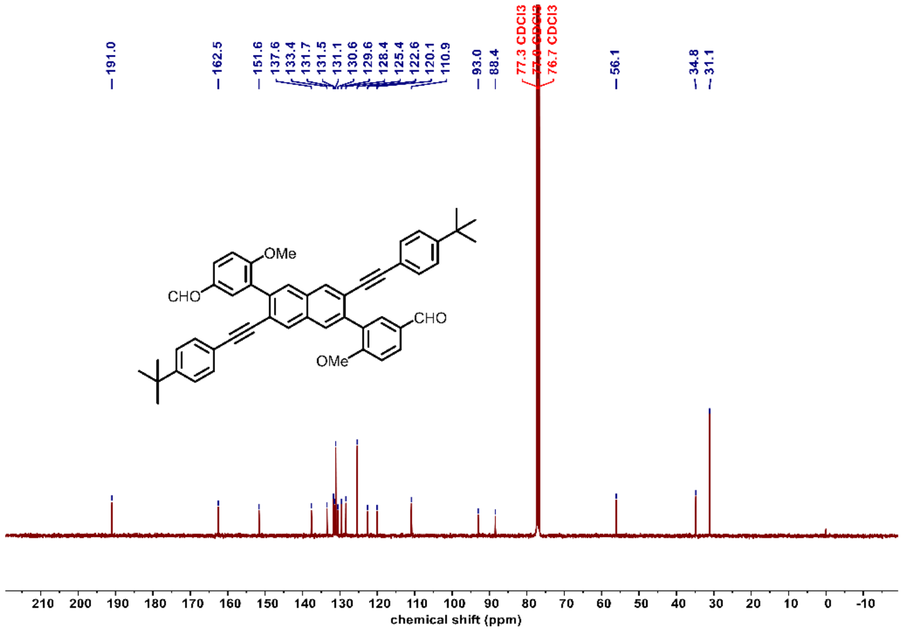

Figure S55 ^{13}C NMR spectrum of compound **12** (101 MHz, $CDCl_3$, 298 K)

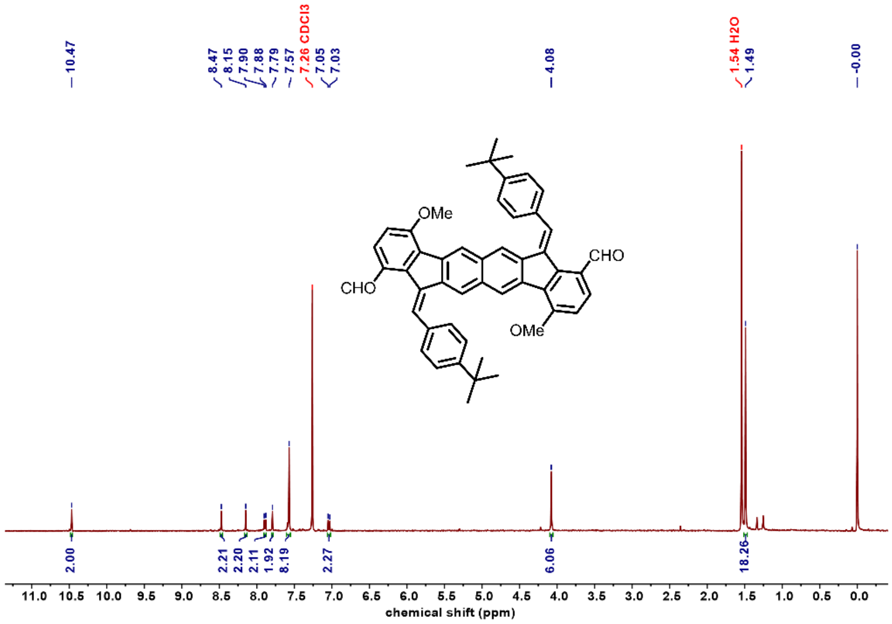

Figure S56 ^{1}H NMR spectrum of compound **13** (400 MHz, $CDCl_3$, 298 K)

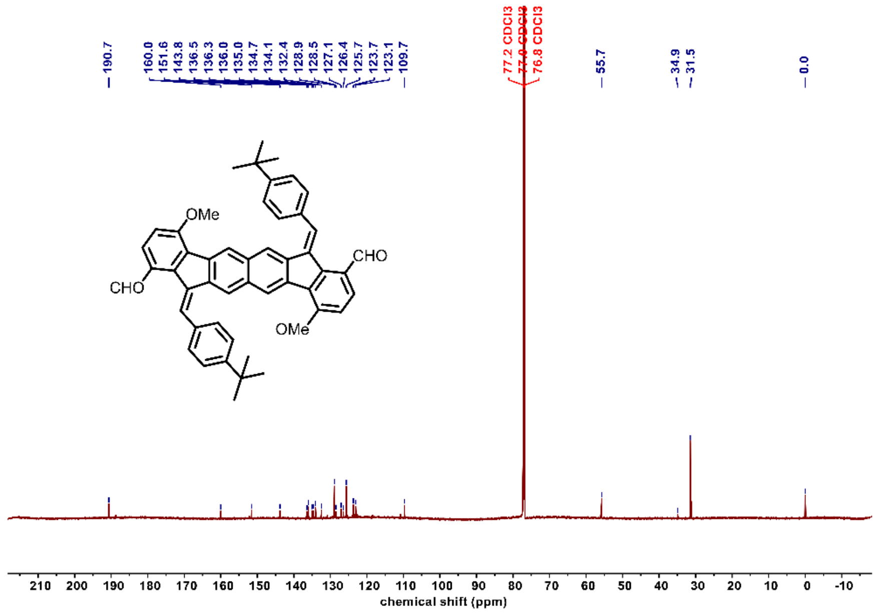

Figure S57 ^{13}C NMR spectrum of compound **13** (101 MHz, $CDCl_3$, 298 K)

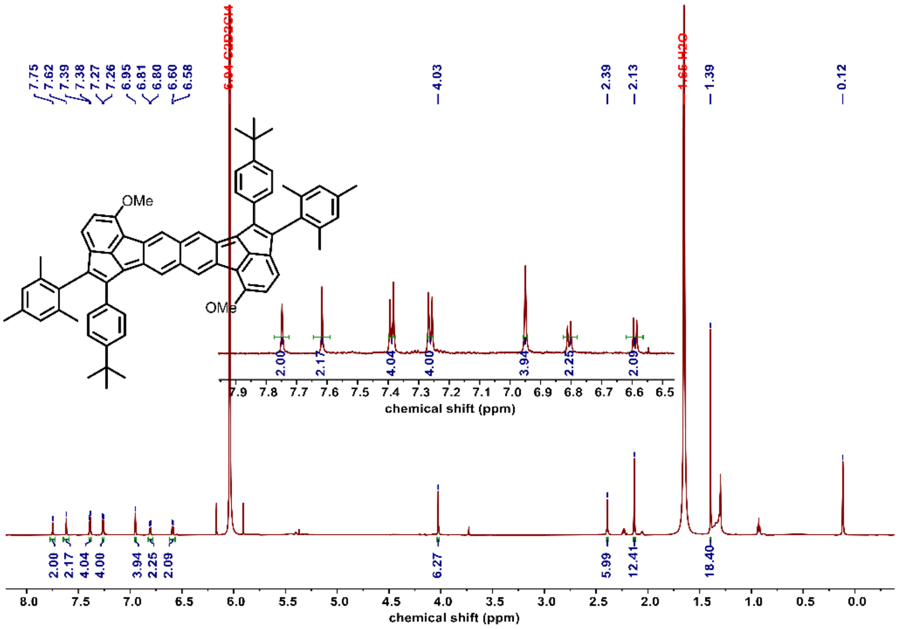

Figure S58 ^{1}H NMR spectrum of compound **2** (700 MHz, $C_2D_2Cl_4$, 298 K)

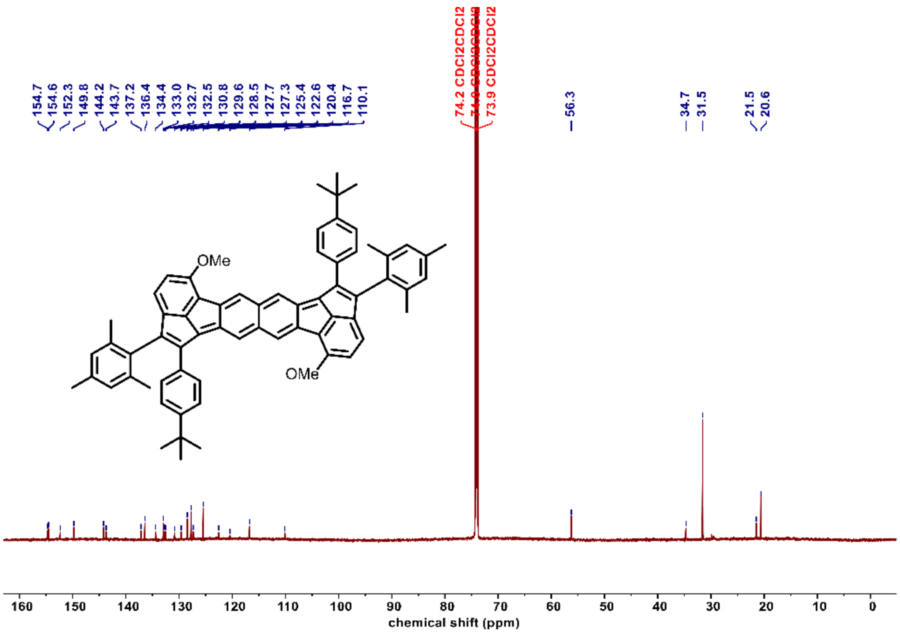

Figure S59 ^{13}C NMR spectrum of compound **2** (176 MHz, $C_2D_2Cl_4$, 298 K)

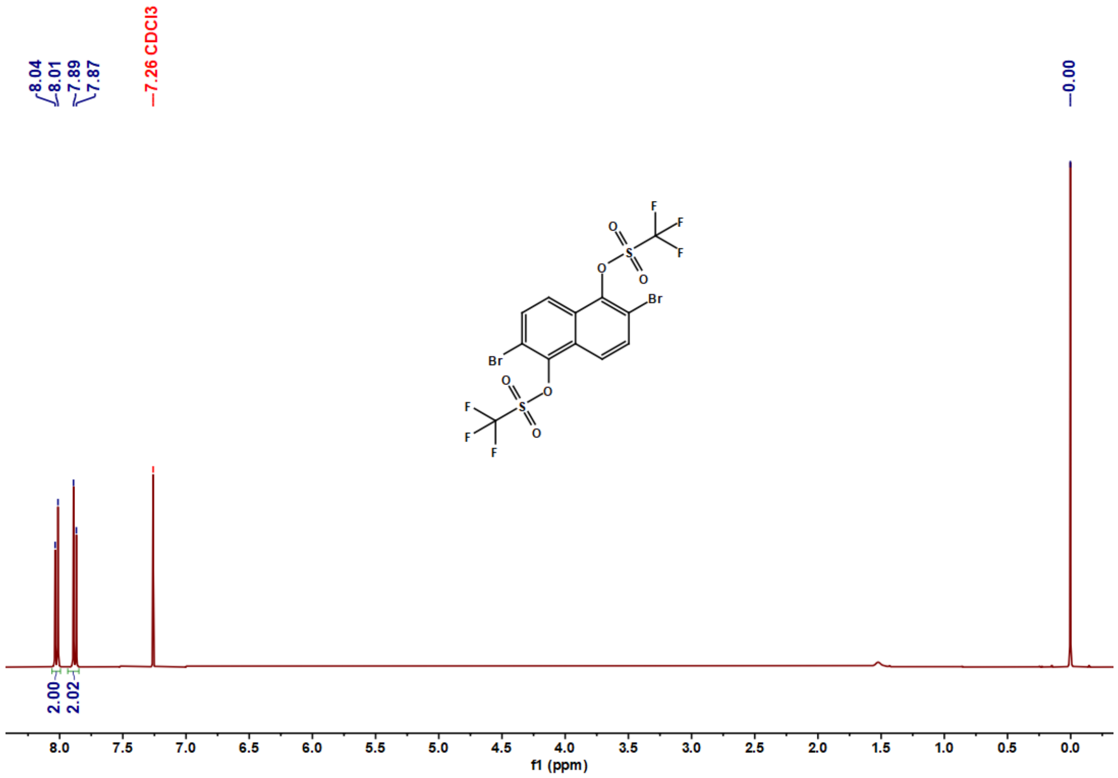

Figure S60 ^{1}H NMR spectrum of compound **16** (400 MHz, $CDCl_3$, 298 K)

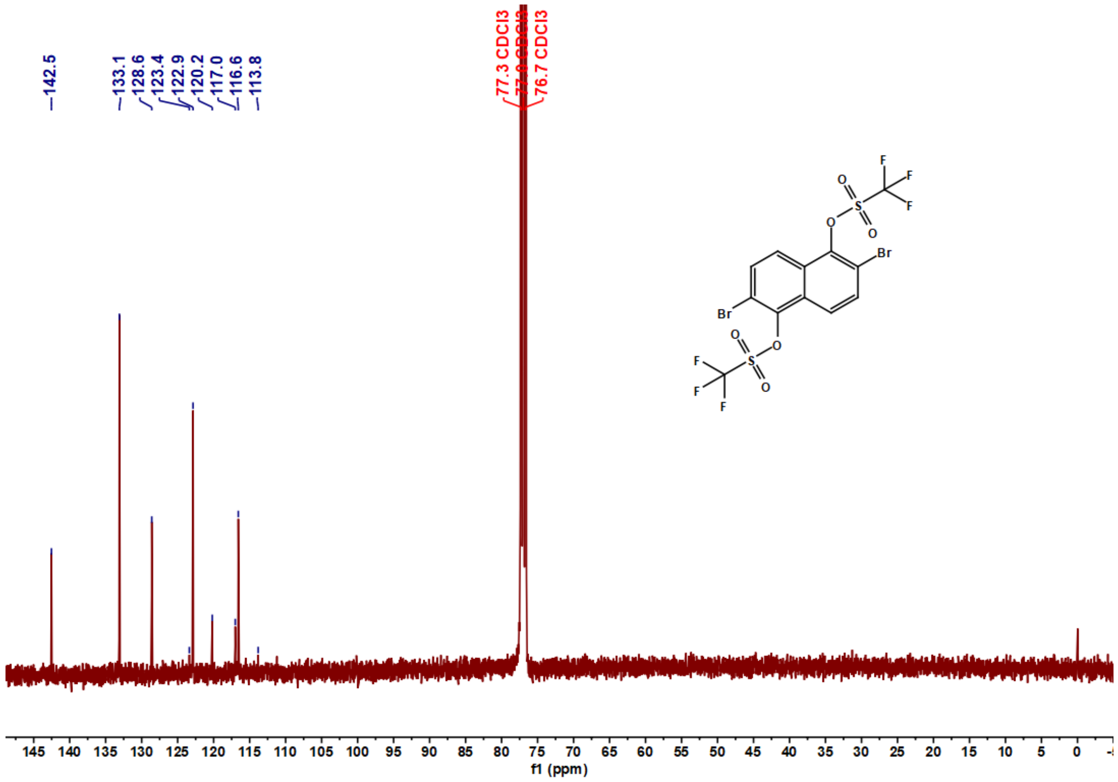

Figure S61 ^{13}C NMR spectrum of compound **16** (101 MHz, $CDCl_3$, 298 K)

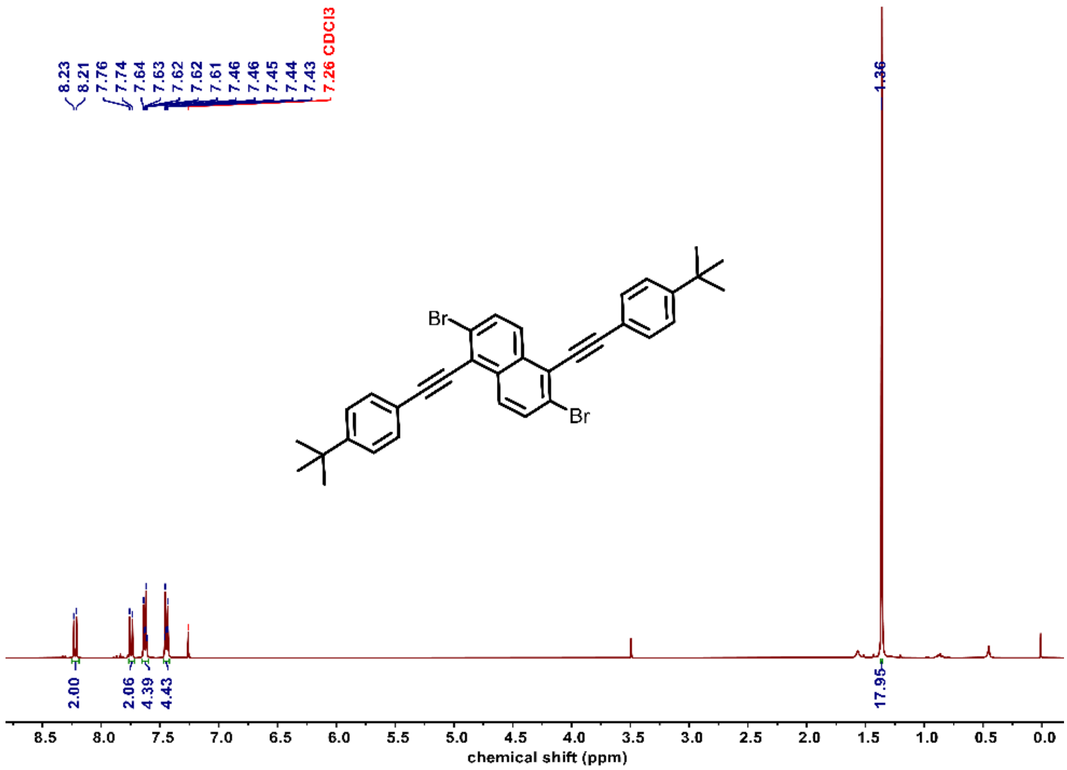

Figure S62 ^{1}H NMR spectrum of compound **17** (400 MHz, $CDCl_3$, 298 K)

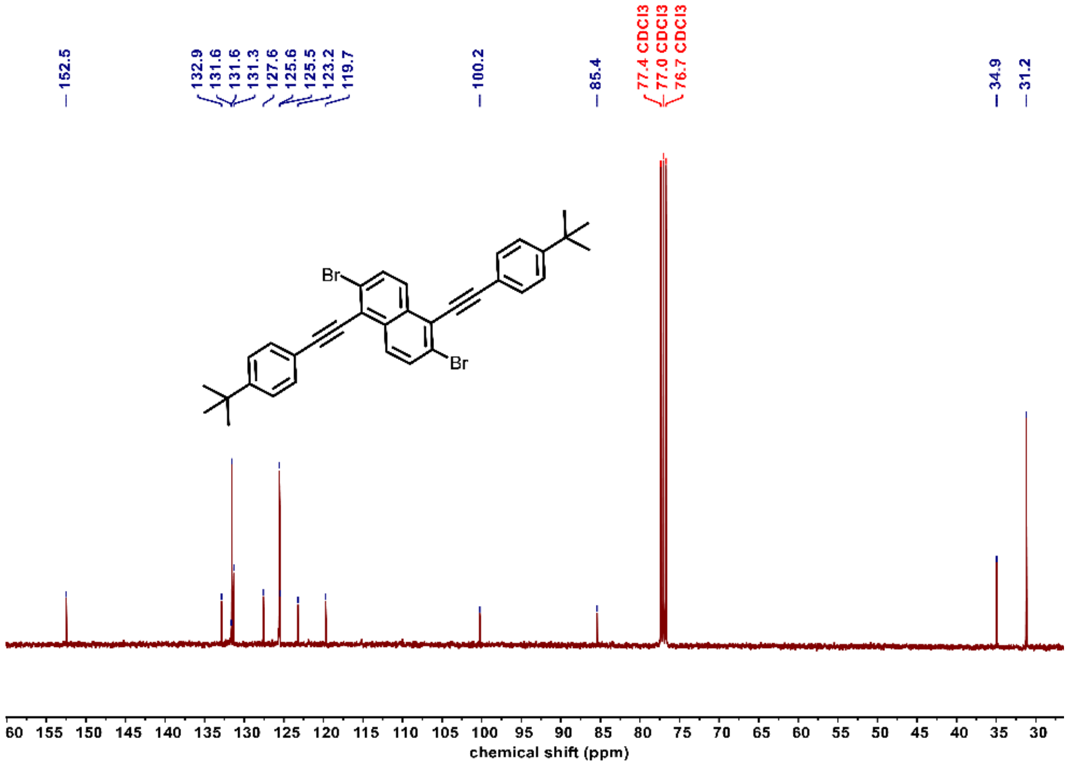

Figure S63 ^{13}C NMR spectrum of compound **17** (101 MHz, $CDCl_3$, 298 K)

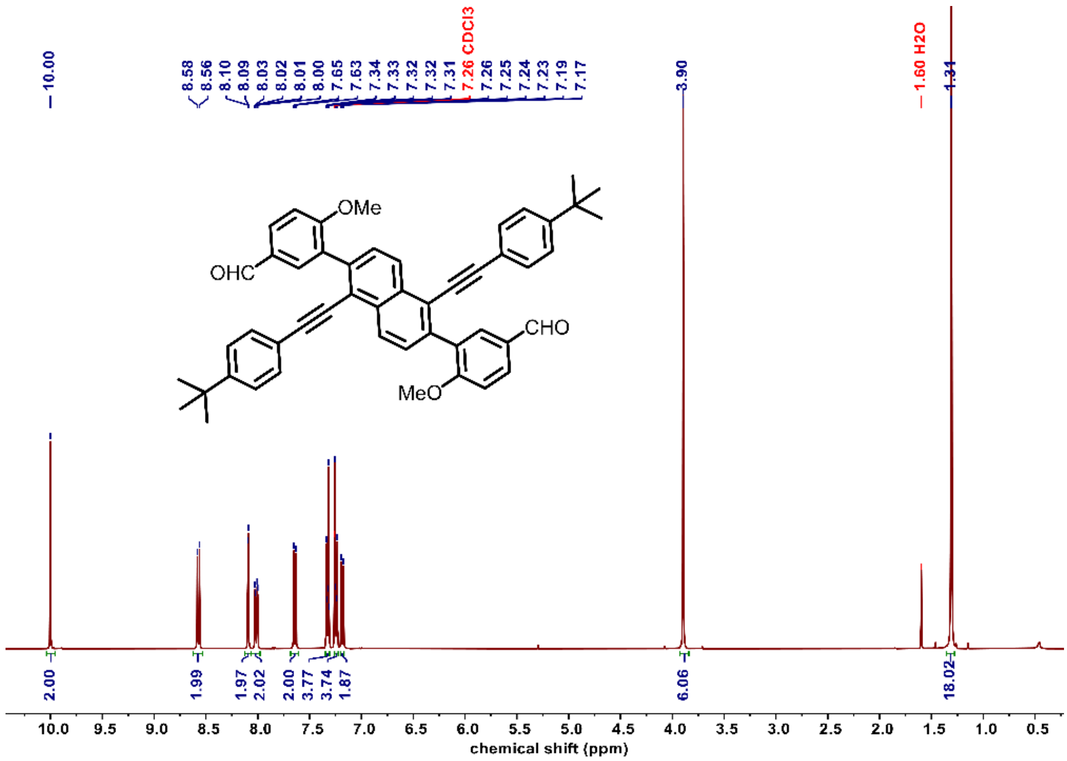

Figure S64 ^{1}H NMR spectrum of compound **18** (400 MHz, $CDCl_3$, 298 K)

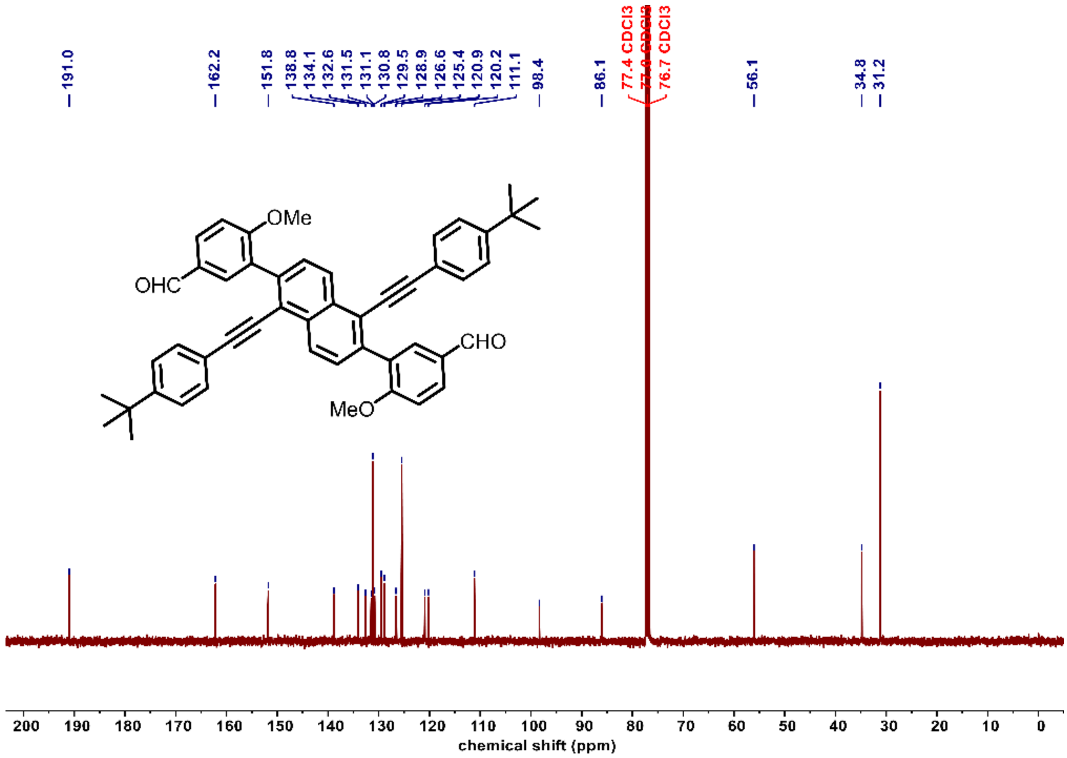

Figure S65 ^{13}C NMR spectrum of compound **18** (101 MHz, $CDCl_3$, 298 K)

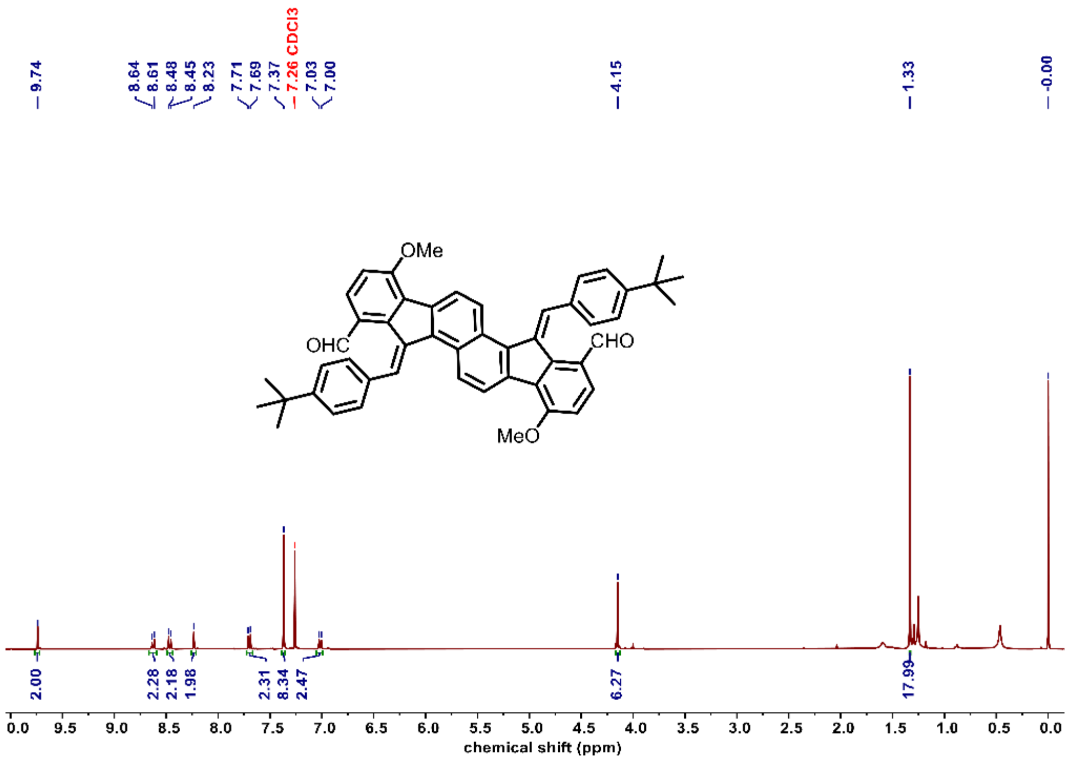

Figure S66 ^{1}H NMR spectrum of compound **19** (400 MHz, $CDCl_3$, 298 K)

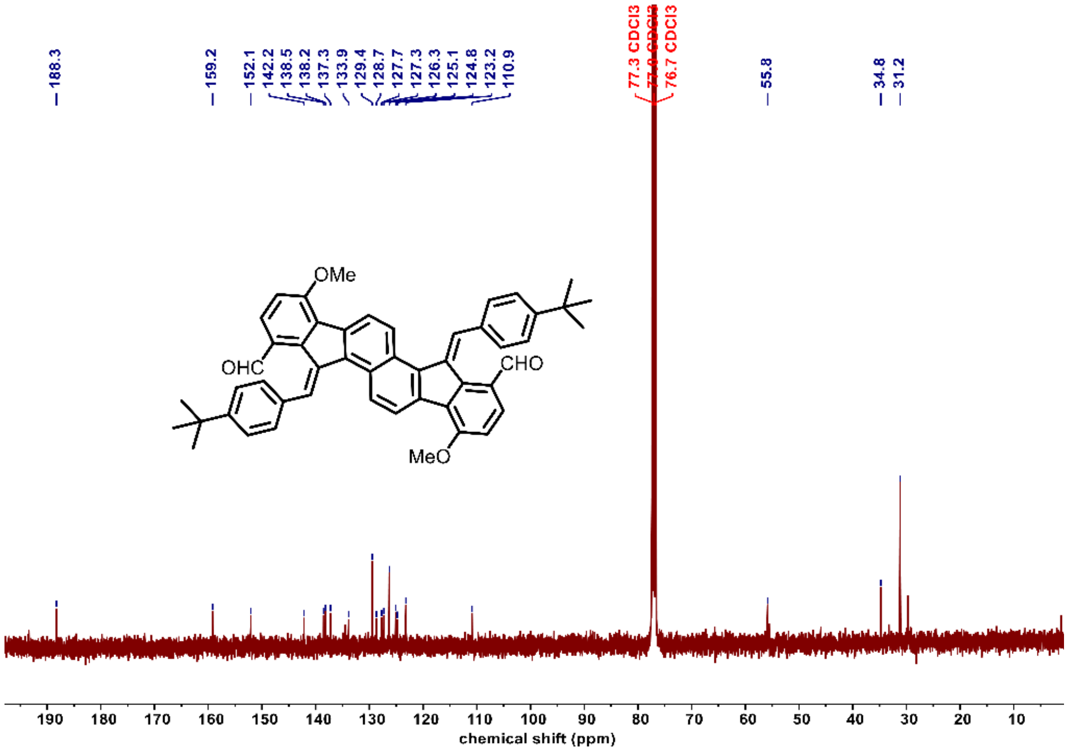

Figure S67 ^{13}C NMR spectrum of compound **19** (101 MHz, $CDCl_3$, 298 K)

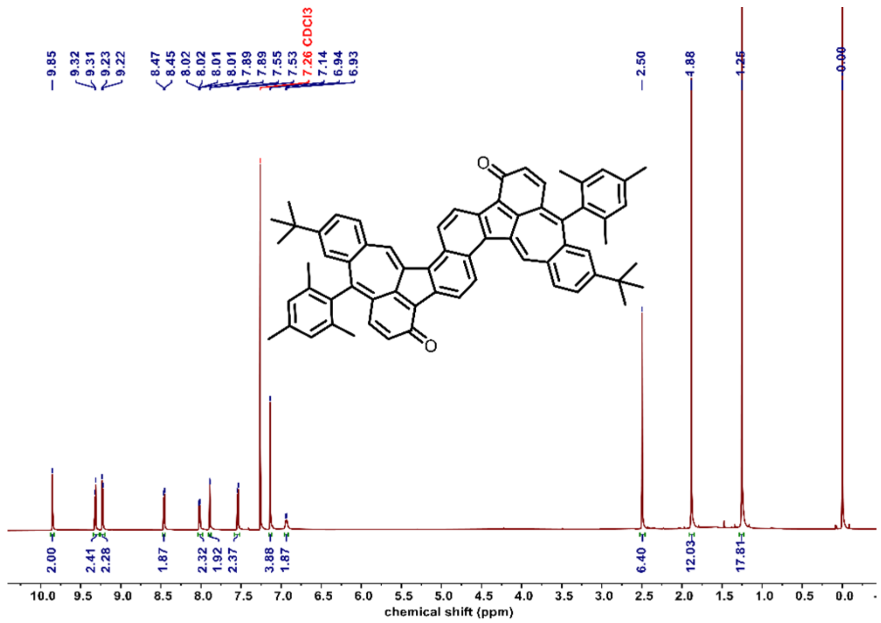

Figure S68 ^{1}H NMR spectrum of compound **3** (400 MHz, $CDCl_3$, 298 K)

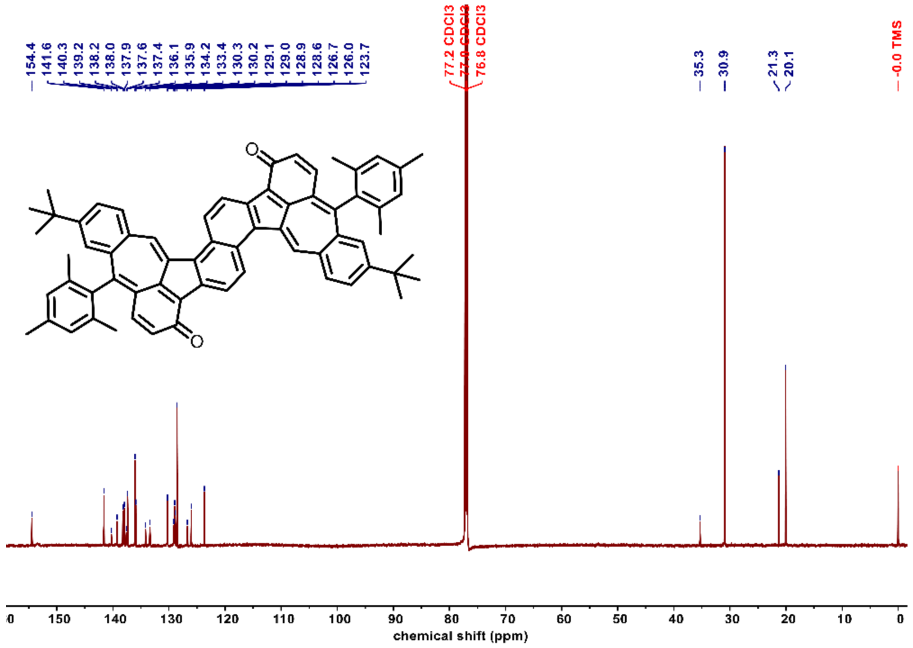

Figure S69 ^{13}C NMR spectrum of compound **3** (101 MHz, $CDCl_3$, 298 K)

16. HRMS spectra

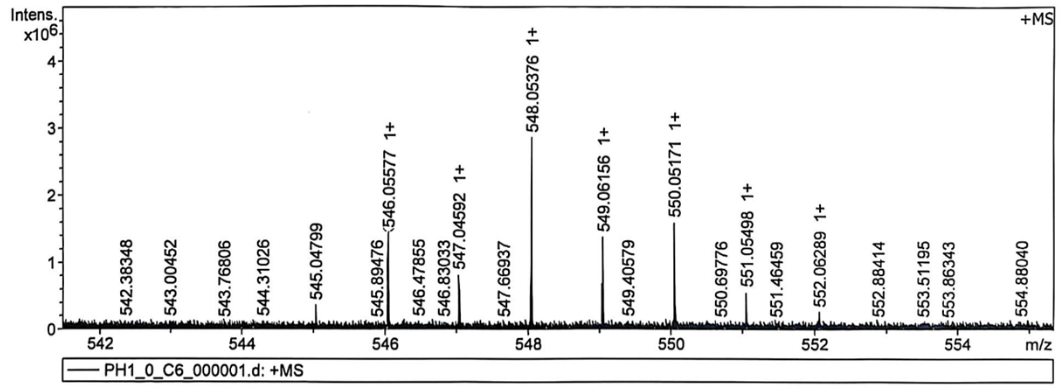

Meas. m/z	#	Ion Formula	Score	m/z	err [ppm]	Mean err [ppm]	mSigma	rdb	e⁻ Conf	N-Rule
546.055769	1	C30H28Br2	100.00	546.055227	-1.0	-0.5	25.0	16.0	odd	ok

Figure S70 HRMS spectrum of compound **5**

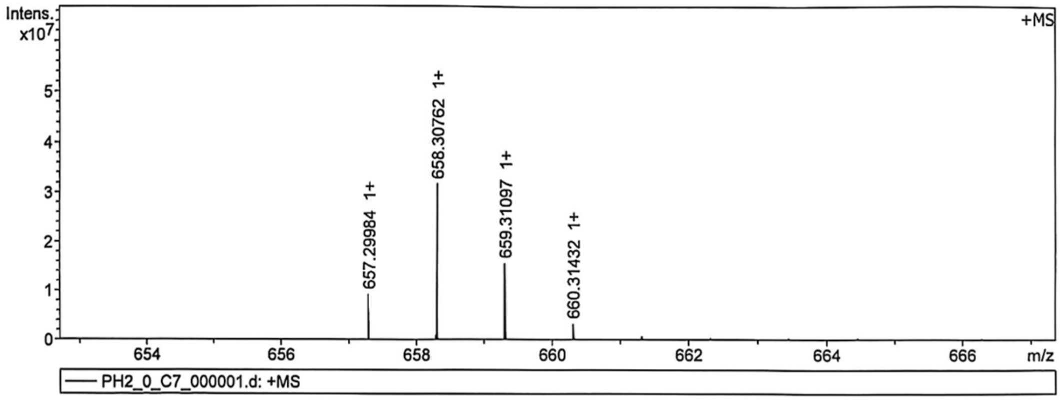

Meas. m/z	#	Ion Formula	Score	m/z	err [ppm]	Mean err [ppm]	mSigma	rdb	e⁻ Conf	N-Rule
658.307623	1	C46H42O4	100.00	658.307761	-0.2	0.2	16.8	26.0	odd	ok

Figure S71 HRMS spectrum of compound **6**

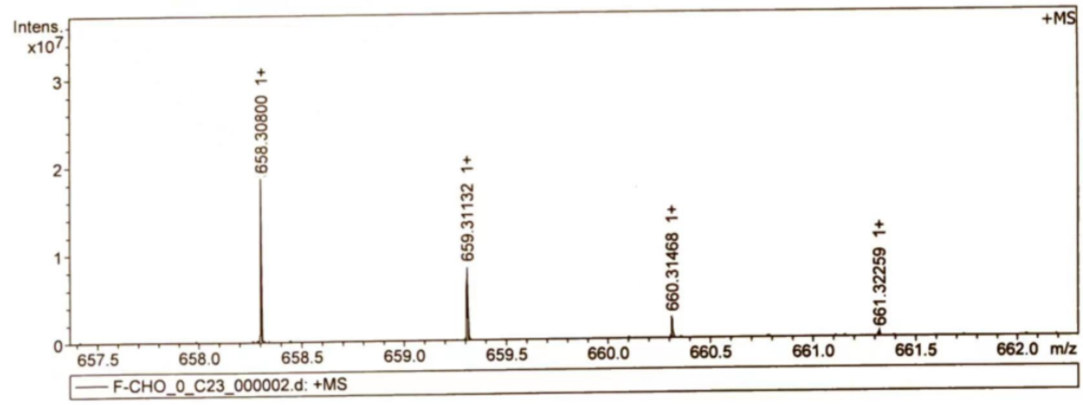

Meas. m/z	#	Ion Formula	Score	m/z	err [ppm]	Mean err [ppm]	mSigma	rdb	e⁻ Conf	N-Rule
658.308004	1	C46H42O4	100.00	658.307761	0.4	-0.3	31.1	26.0	odd	ok

Figure S72 HRMS spectrum of compound **7**

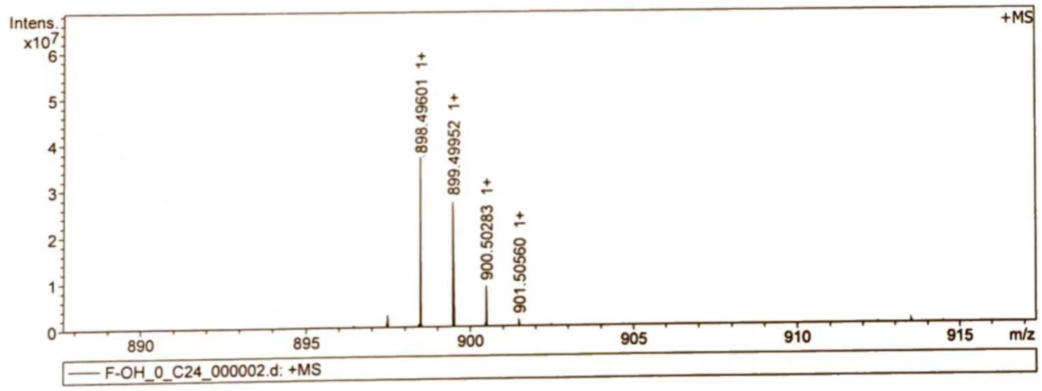

Meas. m/z	#	Ion Formula	Score	m/z	err [ppm]	Mean err [ppm]	mSigma	rdb	e⁻ Conf	N-Rule
898.496008	1	C64H66O4	100.00	898.495562	-0.5	-0.6	22.6	32.0	odd	ok

Figure S73 HRMS spectrum of compound **8**

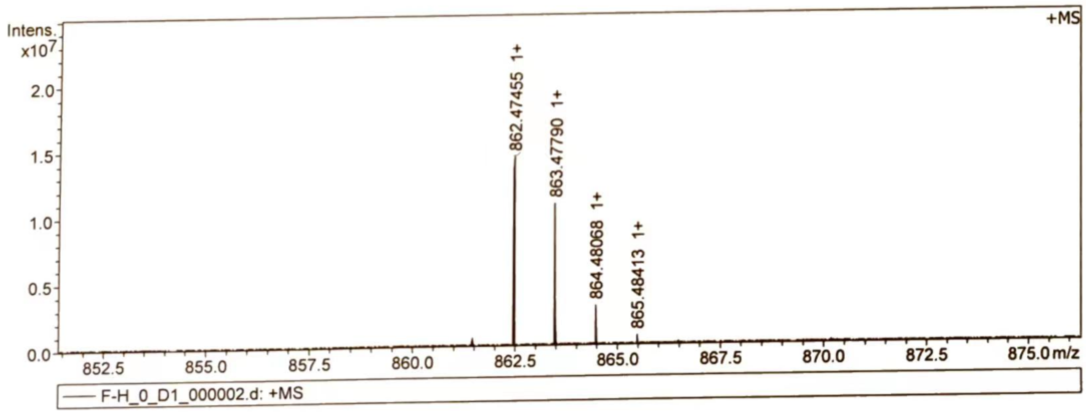

Meas. m/z	#	Ion Formula	Score	m/z	err [ppm]	Mean err [ppm]	mSigma	rdb	e⁻ Conf	N-Rule
862.474553	1	C64H62O2	100.00	862.474433	0.1	-0.0	28.1	34.0	odd	ok

Figure S74 HRMS spectrum of compound **9**

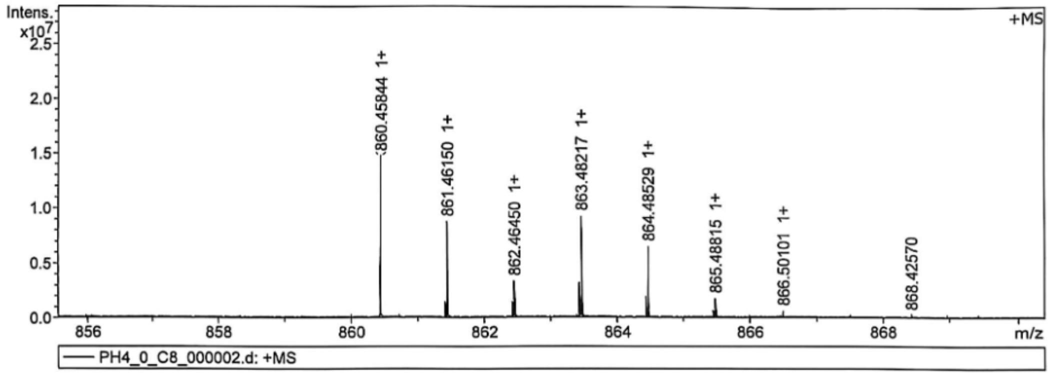

Meas. m/z	#	Ion Formula	Score	m/z	err [ppm]	Mean err [ppm]	mSigma	rdb	e⁻ Conf	N-Rule
860.458436	1	C64H60O2	100.00	860.458783	-0.4	0.6	46.6	35.0	odd	ok

Figure S75 HRMS spectrum of compound **1**

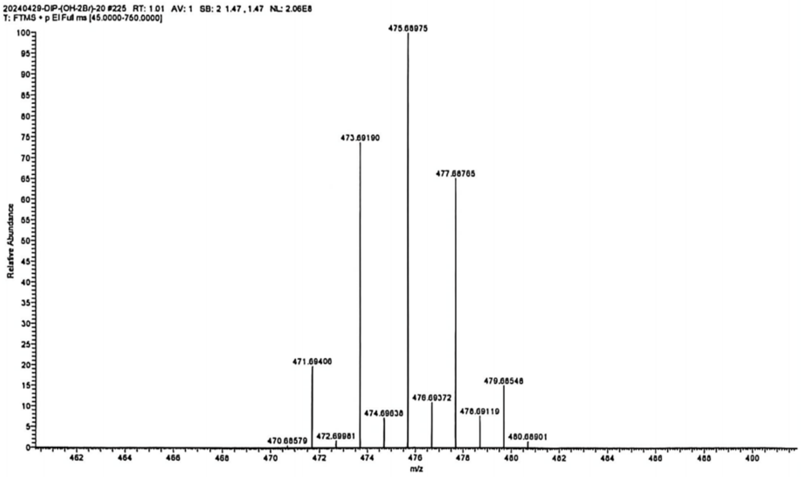

C10 H4 O2 Br4 7.0 0.273PPM

Figure S76 HRMS spectrum of compound **Na-4Br-2OH**

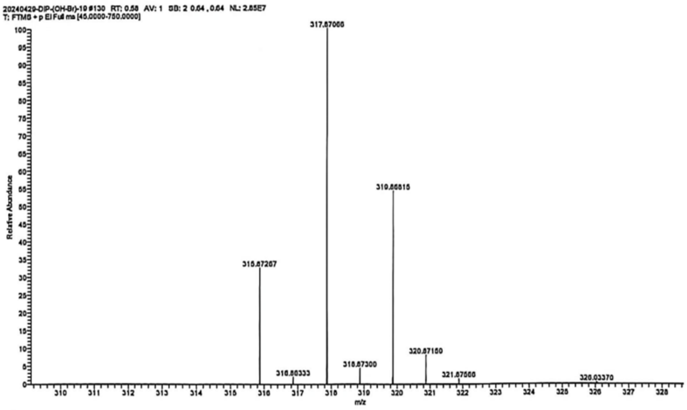

C10 H6 O2 Br2 7.0 -0.748PPM

Figure S77 HRMS spectrum of compound **Na-2Br-2OH**

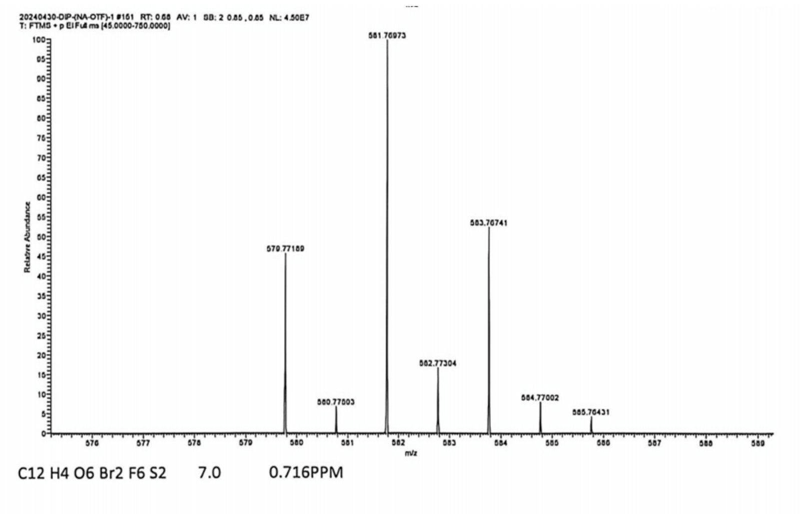

Figure S78 HRMS spectrum of compound **10**

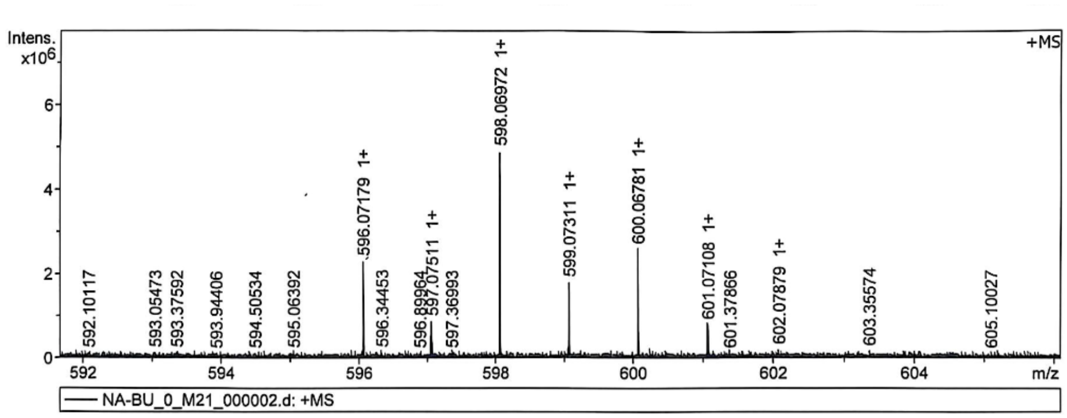

Meas. m/z	#	Ion Formula	Score	m/z	err [ppm]	Mean err [ppm]	mSigma	rdb	e⁻ Conf	N-Rule
596.071786	1	C34H30Br2	100.00	596.070877	-1.5	-0.9	11.8	19.0	odd	ok

Figure S79 HRMS spectrum of compound **11**

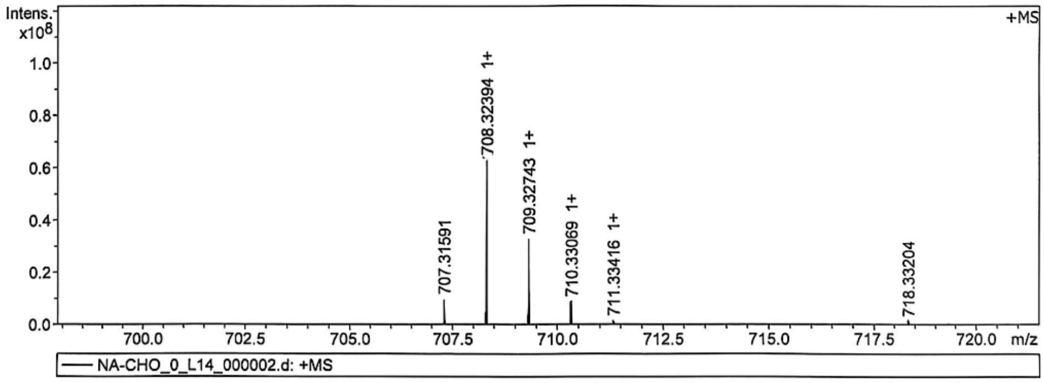

Meas. m/z	#	Ion Formula	Score	m/z	err [ppm]	Mean err [ppm]	mSigma	rdb	e⁻ Conf	N-Rule
708.323937	1	C50H44O4	100.00	708.323411	0.7	-0.8	15.2	29.0	odd	ok

Figure S80 HRMS spectrum of compound **12**

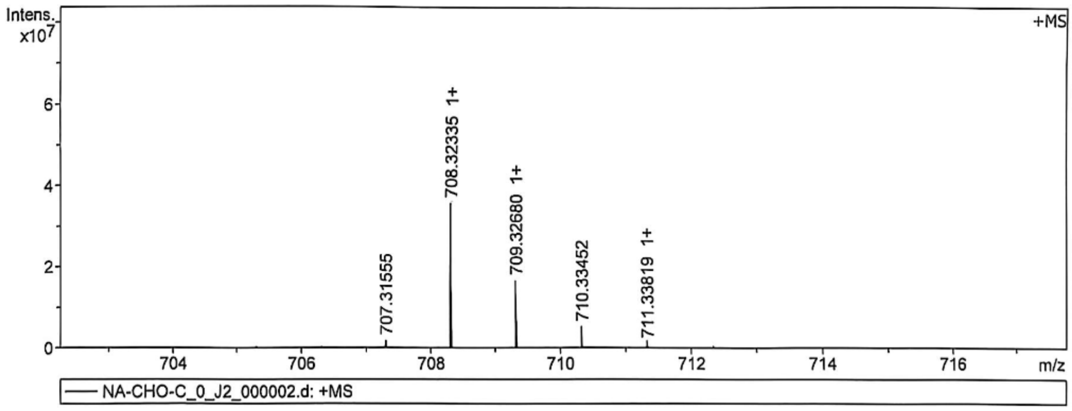

Meas. m/z	#	Ion Formula	Score	m/z	err [ppm]	Mean err [ppm]	mSigma	rdb	e⁻ Conf	N-Rule
708.323350	1	C50H44O4	100.00	708.323411	-0.1	0.0	42.4	29.0	odd	ok

Figure S81 HRMS spectrum of compound **13**

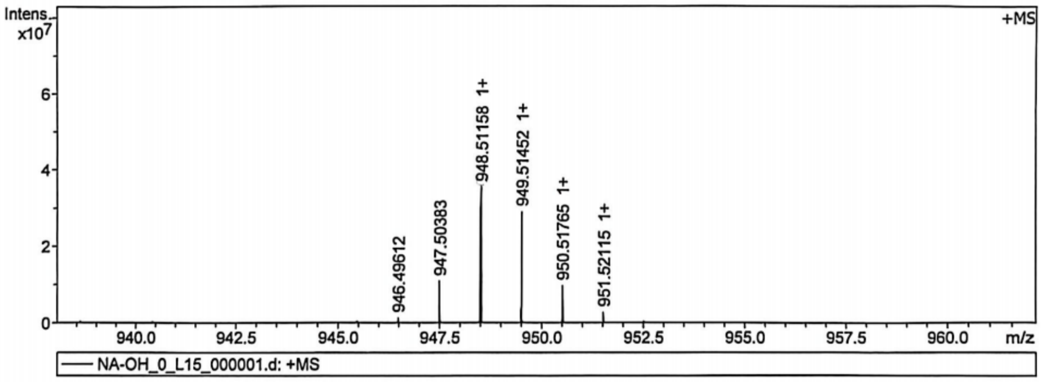

Meas. m/z	#	Ion Formula	Score	m/z	err [ppm]	Mean err [ppm]	mSigma	rdb	e⁻ Conf	N-Rule
948.511578	1	C68H68O4	100.00	948.511212	-0.4	-0.1	33.9	35.0	odd	ok

Figure S82 HRMS spectrum of compound **14**

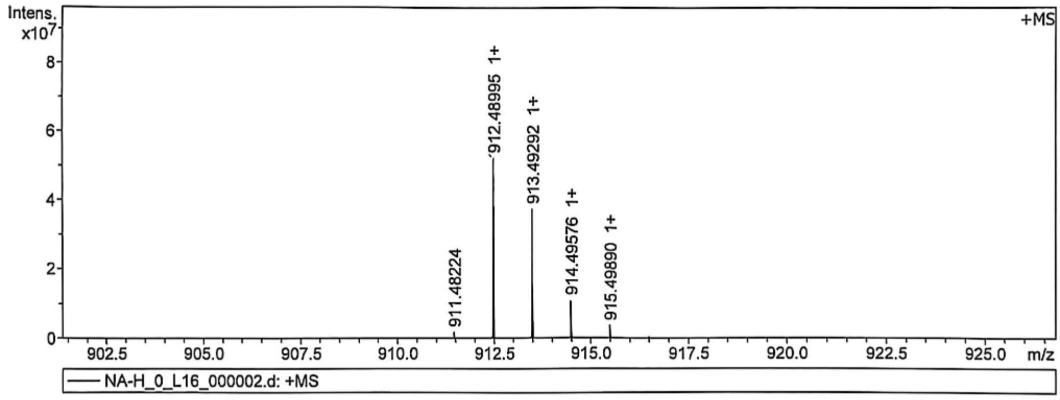

Meas. m/z	#	Ion Formula	Score	m/z	err [ppm]	Mean err [ppm]	mSigma	rdb	e⁻ Conf	N-Rule
912.489955	1	C68H64O2	100.00	912.490083	-0.1	0.5	32.7	37.0	odd	ok

Figure S83 HRMS spectrum of compound **15**

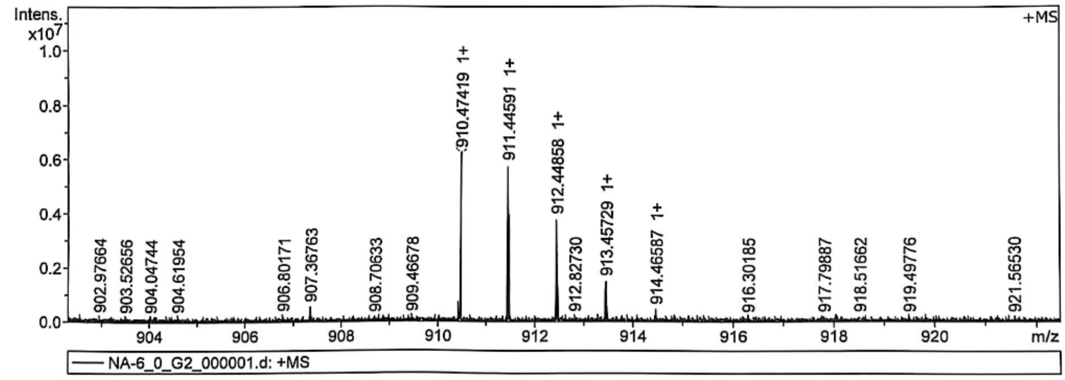

Meas. m/z	#	Ion Formula	Score	m/z	err [ppm]	Mean err [ppm]	mSigma	rdb	e⁻ Conf	N-Rule
910.474189	1	C68H62O2	100.00	910.474433	-0.3	0.6	68.5	38.0	odd	ok

Figure S84 HRMS spectrum of compound **2**

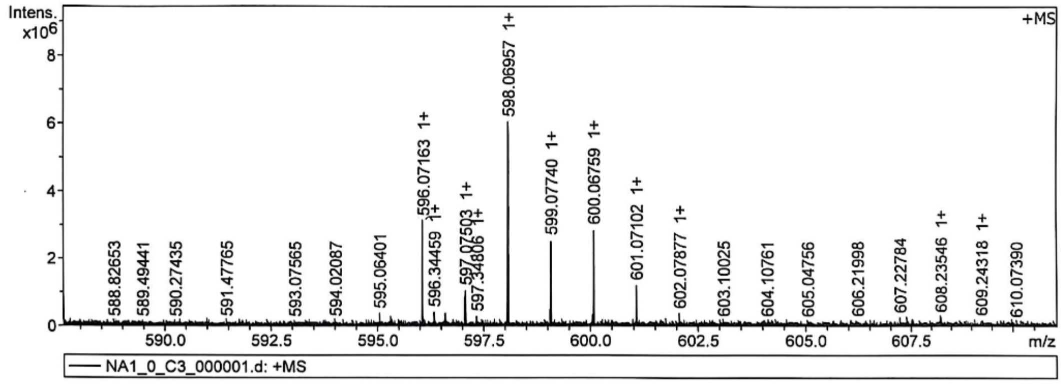

Meas. m/z	#	Ion Formula	Score	m/z	err [ppm]	Mean err [ppm]	mSigma	rdb	e⁻ Conf	N-Rule
596.071627	1	C34H30Br2	100.00	596.070877	1.3	-0.7	26.9	19.0	odd	ok

Figure S85 HRMS spectrum of compound **17**

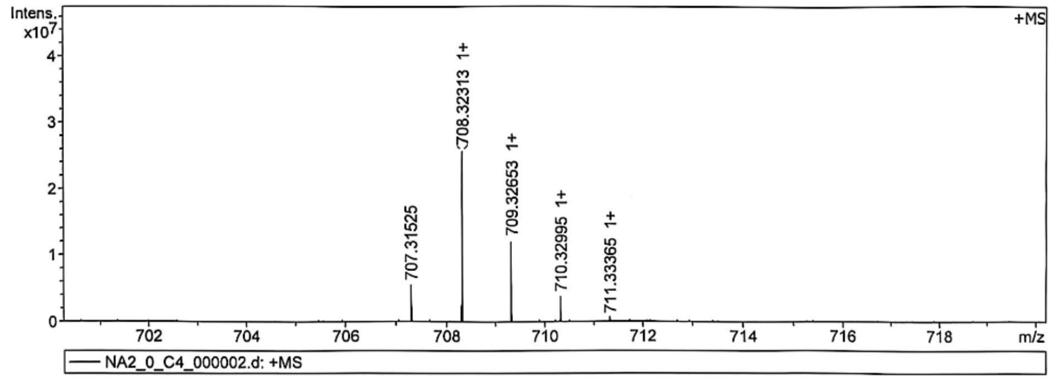

Meas. m/z	#	Ion Formula	Score	m/z	err [ppm]	Mean err [ppm]	mSigma	rdb	e⁻ Conf	N-Rule
708.323135	1	C50H44O4	100.00	708.323411	-0.4	0.4	31.7	29.0	odd	ok

Figure S86 HRMS spectrum of compound **18**

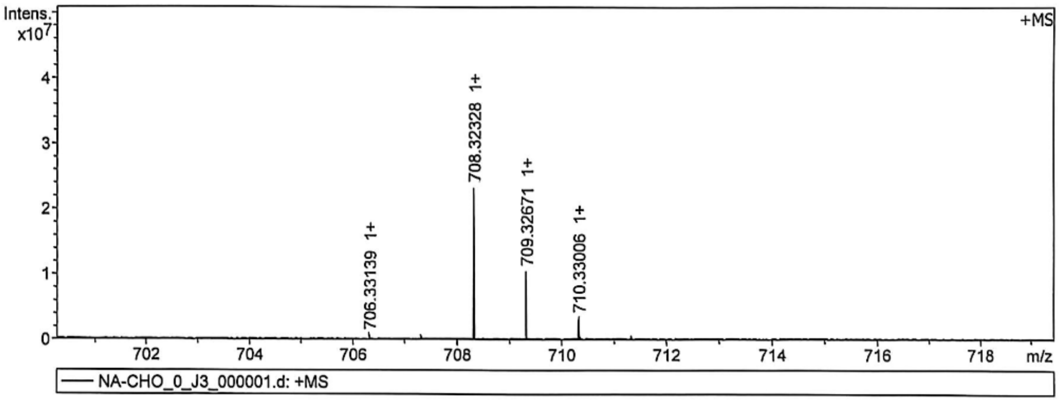

Meas. m/z	#	Ion Formula	Score	m/z	err [ppm]	Mean err [ppm]	mSigma	rdb	e⁻ Conf	N-Rule
708.323280	1	C50H44O4	100.00	708.323411	-0.2	0.1	45.8	29.0	odd	ok

Figure S87 HRMS spectrum of compound **19**

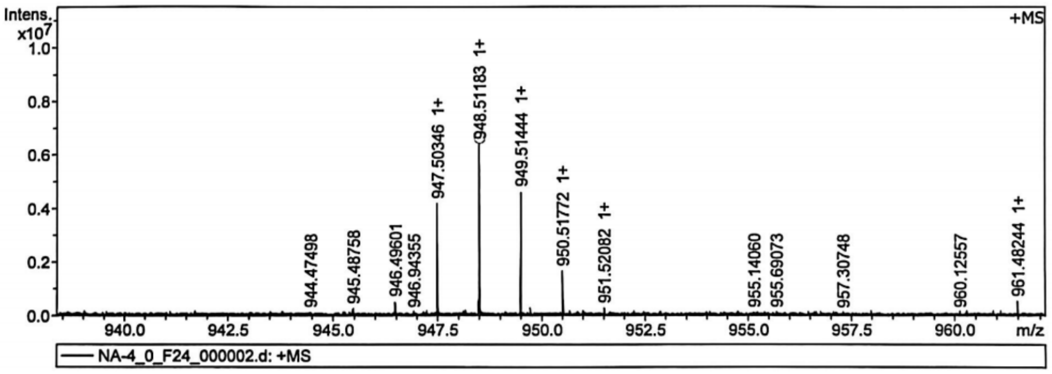

Meas. m/z	#	Ion Formula	Score	m/z	err [ppm]	Mean err [ppm]	mSigma	rdb	e⁻ Conf	N-Rule
948.511830	1	C68H68O4	100.00	948.511212	0.7	-0.2	19.4	35.0	odd	ok

Figure S88 HRMS spectrum of compound **20**

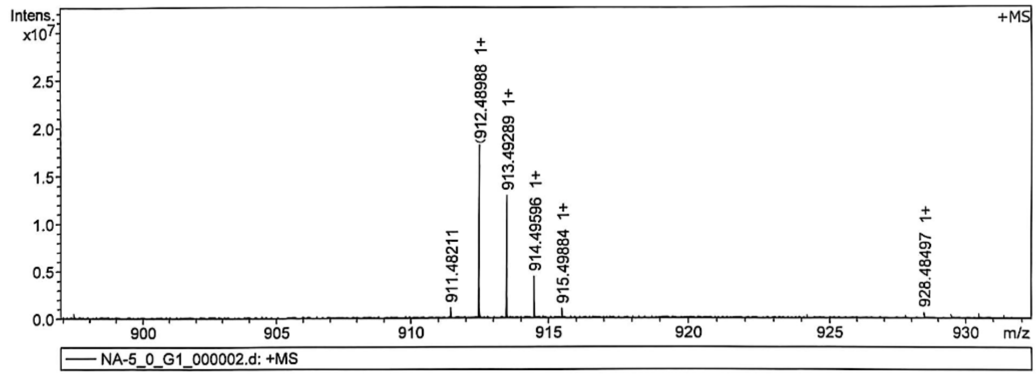

Meas. m/z	#	Ion Formula	Score	m/z	err [ppm]	Mean err [ppm]	mSigma	rdb	e⁻ Conf	N-Rule
912.489877	1	C68H64O2	100.00	912.490083	-0.2	0.5	24.5	37.0	odd	ok

Figure S89 HRMS spectrum of compound **21**

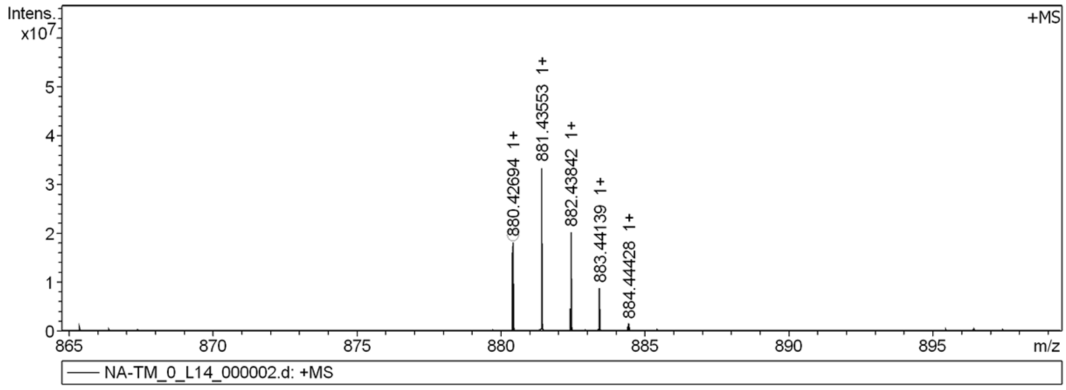

Meas. m/z	#	Ion Formula	Score	m/z	err [ppm]	Mean err [ppm]	mSigma	rdb	e⁻ Conf	N-Rule
880.426943	1	C66H56O2	100.00	880.427482	0.6	1.4	15.3	39.0	odd	ok

Figure S90 HRMS spectrum of compound **3**

17. Reference

[S1] M. J. Frisch, G. W. Trucks, H. B. Schlegel, G. E. Scuseria, M. A. Robb, J. R. Cheeseman, G. Scalmani, V. Barone, G. A. Petersson, H. Nakatsuji, X. Li, M. Caricato, A. V. Marenich, J. Bloino, B. G. Janesko, R. Gomperts, B. Mennucci, H. P. Hratchian, J. V. Ortiz, A. F. Izmaylov, J. L. Sonnenberg, D. Williams-Young, F. Ding, F. Lipparini, F. Egidi, J. Goings, B. Peng, A. Petrone, T. Henderson, D. Ranasinghe, V. G. Zakrzewski, J. Gao, N. Rega, G. Zheng, W. Liang, M. Hada, M. Ehara, K. Toyota, R. Fukuda, J. Hasegawa, M. Ishida, T. Nakajima, Y. Honda, O. Kitao, H. Nakai, T. Vreven, K. Throssell, J. A. Montgomery Jr, J. E. Peralta, F. Ogliaro, M. J. Bearpark, J. J. Heyd, E. N. Brothers, K. N. Kudin, V. N. Staroverov, T. A. Keith, R. Kobayashi, J. Normand, K. Raghavachari, A. P. Rendell, J. C. Burant, S. S. Iyengar, J. Tomasi, M. Cossi, J. M. Millam, M. Klene, C. Adamo, R. Cammi, J. W. Ochterski, R. L. Martin, K. Morokuma, O. Farkas, J. B. Foresman, D. J. Fox, *Gaussian 09, Revision E.01*, Gaussian, Inc., Wallingford CT, **2013**.

[S2] P. J. Stephens, F. J. Devlin, C. F. Chabalowski, M. J. Frisch, *J. Phys. Chem.,* **1994**, *98*, 11623-11627.

[S3] S. Grimme, J. Antony, S. Ehrlich, H. Krieg, *J. Chem. Phys.,* **2010**, *132*, 154104.

[S4] P. C. Hariharan, J. A. Pople, *Theor. Chim. Acta,* **1973**, *28*, 213-222.

[S5] F. Furche, R. Ahlrichs, *J. Chem. Phys.,* **2002**, *117*, 7433-7447.

[S6] Z. Chen, C. S. Wannere, C. Corminboeuf, R. Puchta, P. V. R. Schleyer, *Chem. Rev.*, **2005**, *105*, 3842-3888.

[S7] D. Geuenich, K. Hess, F. Köhler, R. Herges, *Chem. Rev.*, **2005**, *105*, 3758-3772.

[S8] Z. Liu, T. Lu, Q. Chen, *Carbon*, **2020**, *165*, 468-475.

[S9] T. Lu, F. Chen, *J. Comput. Chem.*, **2012**, *33*, 580-592.

[S10] K. Kamada, K. Ohta, A. Shimizu, T. Kubo, R. Kishi, H. Takahashi, E. Botek, B. Champagne, M. Nakano, *J. Phys. Chem. Lett.* **2010**, *1*, 937-940.

[11] J. K. Mahey, C. B. Pawara, S. B. Kamble, *New J. Chem.*, **2023**, *47*, 18419-18429.
